# Supplementary material for: Composition of Triterpene Glycosides of the Far Eastern Sea Cucumber Cucumaria conicospermium Levin et Stepanov; Structure Elucidation of Five Minor Conicospermiumosides A3-1, A3-2, A3-3, A7-1, and A7-2; Cytotoxicity of the Glycosides Against Human Breast Cancer Cell Lines; Structure–Activity Relationships
Source: Mar Drugs. 2024 Dec 16;22(12):560. doi: 10.3390/md22120560 (PMC11676834; doi:10.3390/md22120560)
Supplement: Supplementary file 1 [file marinedrugs-22-00560-s001.zip › marinedrugs-3342379-supplementary.pdf]

Supplementary data content page

Title: Composition of Triterpene Glycosides of the Far Eastern Sea Cucumber *Cucumaria conicospermium* Levin et Stepanov; structure elucidation of five minor conicospermiumosides A_3-1, A_3-2, A_3-3, A_7-1, and A_7-2; Cytotoxicity of the glycosides against Human Breast Cancer Cell Lines; Structure-Activity Relationships

Authors: **Alexandra S. Silchenko [1,*], Ekaterina A. Chingizova [1], Ekaterina S. Menchinskaya [1], Elena A. Zelepuga [1], Anatoly I. Kalinovsky [1], Sergey A. Avilov [1], Kseniya M. Tabakmakher [1], Roman S. Popov [1], Pavel. S. Dmitrenok [1], Salim Sh. Dautov [2], Vladimir I. Kalinin [1,*]**

Address: [1]*G.B. Elyakov Pacific Institute of Bioorganic Chemistry, Far Eastern Branch of Russian Academy of Sciences, Pr. 100-let Vladivostoka 159, 690022 Vladivostok, Russia*
[2] *A.V. Zhirmunsky National Scientific Center of Marine Biology, Far Eastern Branch, Russian Academy of Sciences, Palchevskogo str, 17, 690022 Vladivostok, Russia*
Correspondence: silchenko_als@piboc.dvo.ru; kalininv@piboc.dvo.ru, Tel.: +7-423-231-1168

Content:

Figure S1. Assigned ^{13}C NMR spectrum of djakonovioside A (**6**) isolated from *C. conicospermium*
Figure S2. Assigned ^{13}C NMR spectrum of djakonovioside B_2 (**13**) isolated from *C. conicospermium*
Figure S3. Assigned ^{13}C NMR spectrum of okhotoside A_1-1 (**7**) isolated from *C. conicospermium*
Figure S4. Assigned ^{13}C NMR spectrum of frondoside A (**10**) isolated from *C. conicospermium*
Figure S5. Assigned ^{13}C NMR spectrum of okhotoside B_1 (**8**) isolated from *C. conicospermium*
Figure S6. Assigned ^{13}C NMR spectrum of okhotoside A_2-1 (**11**) isolated from *C. conicospermium*
Figure S7. Assigned ^{13}C NMR spectrum of okhotoside B_2 (**9**) isolated from *C. conicospermium*
Figure S8. Assigned ^{13}C NMR spectrum of cucumarioside A_2-5 (**12**) isolated from *C. conicospermium*
Figure S9. Assigned ^{13}C NMR spectrum of koreoside A isolated from (**16**) *C. conicospermium*
Figure S10. Assigned ^{13}C NMR spectrum of isokoreoside A (**17**) isolated from *C. conicospermium*
Figure S11. Assigned ^{13}C NMR spectrum of frondoside A_7-4 (**14**) isolated from *C. conicospermium*
Figure S12. Assigned ^{13}C NMR spectrum of djakonovioside F_1 (**15**) isolated from *C. conicospermium*
Figure S13. The ^{13}C NMR (176.04 MHz) spectrum of conicospermiumoside A_3-1 (**1**) in C_5D_5N/D_2O (4/1)
Figure S14. The ^{1}H NMR (700.13 MHz) spectrum of conicospermiumoside A_3-1 (**1**) in C_5D_5N/D_2O (4/1)
Figure S15. The COSY (700.13 MHz) spectrum of conicospermiumoside A_3-1 (**1**) in C_5D_5N/D_2O (4/1)
Figure S16. The HSQC (700.13 MHz) spectrum of conicospermiumoside A_3-1 (**1**) in C_5D_5N/D_2O (4/1)
Figure S17. The ROESY (700.13 MHz) spectrum of conicospermiumoside A_3-1 (**1**) in C_5D_5N/D_2O (4/1)
Figure S18. The HMBC (700.13 MHz) spectrum of conicospermiumoside A_3-1 (**1**) in C_5D_5N/D_2O (4/1)
Figure S19. 1 D TOCSY (700.13 MHz) spectra of Xyl1, Qui2, Glc3, MeGlc4, Xyl5 of conicospermiumoside A_3-1 (**1**) in C_5D_5N/D_2O (4/1)
Figure S20. HR-ESI-MS and ESI-MS/MS spectra of conicospermiumoside A_3-1 (**1**)
Figure S21. The ^{13}C NMR (176.04 MHz) spectrum of conicospermiumoside A_3-2 (**2**) in C_5D_5N/D_2O (4/1)
Figure S22. The ^{1}H NMR (700.13 MHz) spectrum of conicospermiumoside A_3-2 (**2**) in C_5D_5N/D_2O (4/1)
Figure S23. The COSY (700.13 MHz) spectrum of conicospermiumoside A_3-2 (**2**) in C_5D_5N/D_2O (4/1)
Figure S24. The HSQC (700.13 MHz) spectrum of conicospermiumoside A_3-2 (**2**) in C_5D_5N/D_2O (4/1)
Figure S25. The ROESY (700.13 MHz) spectrum of conicospermiumoside A_3-2 (**2**) in C_5D_5N/D_2O (4/1)
Figure S26. The HMBC (700.13 MHz) spectrum of conicospermiumoside A_3-2 (**2**) in C_5D_5N/D_2O (4/1)
Figure S27. 1 D TOCSY (700.13 MHz) spectra of Xyl1, Qui2, Glc3, MeGlc4, Xyl5 of conicospermiumoside A_3-2 (**2**) in C_5D_5N/D_2O (4/1)
Table S1. ^{13}C and ^{1}H NMR chemical shifts and HMBC and ROESY correlations of carbohydrate moiety of conicospermiumoside A_3-2 (**2**)

Figure S28. HR-ESI-MS and ESI-MS/MS spectra of conicospermiumoside A_3-2 (**2**)
Figure S29. The ^{13}C NMR (176.04 MHz) spectrum of conicospermiumoside A_3-3 (**3**) in C_5D_5N/D_2O (4/1)
Figure S30. The ^{1}H NMR (700.13 MHz) spectrum of conicospermiumoside A_3-3 (**3**) in C_5D_5N/D_2O (4/1)
Figure S31. The COSY (700.13 MHz) spectrum of conicospermiumoside A_3-3 (**3**) in C_5D_5N/D_2O (4/1)
Figure S32. The HSQC (700.13 MHz) spectrum of conicospermiumoside A_3-3 (**3**) in C_5D_5N/D_2O (4/1)
Figure S33. The ROESY (700.13 MHz) spectrum of conicospermiumoside A_3-3 (**3**) in C_5D_5N/D_2O (4/1)
Figure S34. The HMBC (700.13 MHz) spectrum of conicospermiumoside A_3-3 (**3**) in C_5D_5N/D_2O (4/1)
Figure S35. 1 D TOCSY (700.13 MHz) spectra of Xyl1, Qui2, Glc3, MeGlc4, Xyl5 of conicospermiumoside A_3-3 (**3**) in C_5D_5N/D_2O (4/1)
Table S2. ^{13}C and ^{1}H NMR chemical shifts and HMBC and ROESY correlations of carbohydrate moiety of conicospermiumoside A_3-3 (**3**)
Figure S36. HR-ESI-MS and ESI-MS/MS spectra of conicospermiumoside A_3-3 (**3**)
Figure S37. The ^{13}C NMR (176.04 MHz) spectrum of conicospermiumoside A_7-1 (**4**) in C_5D_5N/D_2O (4/1)
Figure S38. The ^{1}H NMR (700.13 MHz) spectrum of conicospermiumoside A_7-1 (**4**) in C_5D_5N/D_2O (4/1)
Figure S39. The COSY (700.13 MHz) spectrum of conicospermiumoside A_7-1 (**4**) in C_5D_5N/D_2O (4/1)
Figure S40. The HSQC (700.13 MHz) spectrum of conicospermiumoside A_7-1 (**4**) in C_5D_5N/D_2O (4/1)
Figure S41. The ROESY (700.13 MHz) spectrum of conicospermiumoside A_7-1 (**4**) in C_5D_5N/D_2O (4/1)
Figure S42. The HMBC (700.13 MHz) spectrum of conicospermiumoside A_7-1 (**4**) in C_5D_5N/D_2O (4/1)
Figure S43. 1 D TOCSY (700.13 MHz) spectra of Xyl1, Qui2, Glc3, MeGlc4, Xyl5 of conicospermiumoside A_7-1 (**4**) in C_5D_5N/D_2O (4/1)
Figure S44. HR-ESI-MS and ESI-MS/MS spectra of conicospermiumoside A_7-1 (**4**)
Figure S45. The ^{13}C NMR (176.04 MHz) spectrum of conicospermiumoside A_7-2 (**5**) in C_5D_5N/D_2O (4/1)
Figure S46. The ^{1}H NMR (700.13 MHz) spectrum of conicospermiumoside A_7-2 (**5**) in C_5D_5N/D_2O (4/1)
Figure S47. The COSY (700.13 MHz) spectrum of conicospermiumoside A_7-2 (**5**) in C_5D_5N/D_2O (4/1)
Figure S48. The HSQC (700.13 MHz) spectrum of conicospermiumoside A_7-2 (**5**) in C_5D_5N/D_2O (4/1)
Figure S49. The ROESY (700.13 MHz) spectrum of conicospermiumoside A_7-2 (**5**) in C_5D_5N/D_2O (4/1)
Figure S50. The HMBC (700.13 MHz) spectrum of conicospermiumoside A_7-2 (**5**) in C_5D_5N/D_2O (4/1)
Figure S51. 1 D TOCSY (700.13 MHz) spectra of Xyl1, Qui2, Glc3, MeGlc4, Xyl5 of conicospermiumoside A_7-2 (**5**) in C_5D_5N/D_2O (4/1)
Table S3. ^{13}C and ^{1}H NMR chemical shifts and HMBC and ROESY correlations of carbohydrate moiety of conicospermiumoside A_7-2 (**5**)
Figure S52. HR-ESI-MS and ESI-MS/MS spectra of conicospermiumoside A_7-2 (**5**)
Figure S53. Assigned ^{13}C NMR spectrum of desulfated derivative okhotoside A_1-1 (**7a**) isolated from *C. conicospermium*
Figure S54. Assigned ^{13}C NMR spectrum of desulfated derivative of okhotoside B_1 (**8a**) isolated from *C. conicospermium*
Figure S55. Cytotoxic effects of okhotoside B_1 (**8**), DS-okhotoside B_1 (**8a**) and cisplatin against MDA-MB-231 and MCF 10A cells for 24 h, 48 h, and 72 h.
Figure S56. Cytotoxic effects of conicospermiumosides A_3-3 (**3**) and A_7-1 (**4**) and cisplatin against MDA-MB-231 and MCF 10A cells for 24 h, 48 h, and 72 h.
Figure S57. Cytotoxic effects of conicospermiumoside A_3-1 (**1**), DS-okhotoside A_1-1 (**7a**) and cisplatin against MDA-MB-231 and MCF 10A cells for 24 h, 48 h, and 72 h.
Figure S58. PLS QSAR model correlation plot reflecting the relationship of predicted and experimental cytotoxic activity against MCF-10A cells.
Figure S59. Correlational matrix of descriptors impacts.

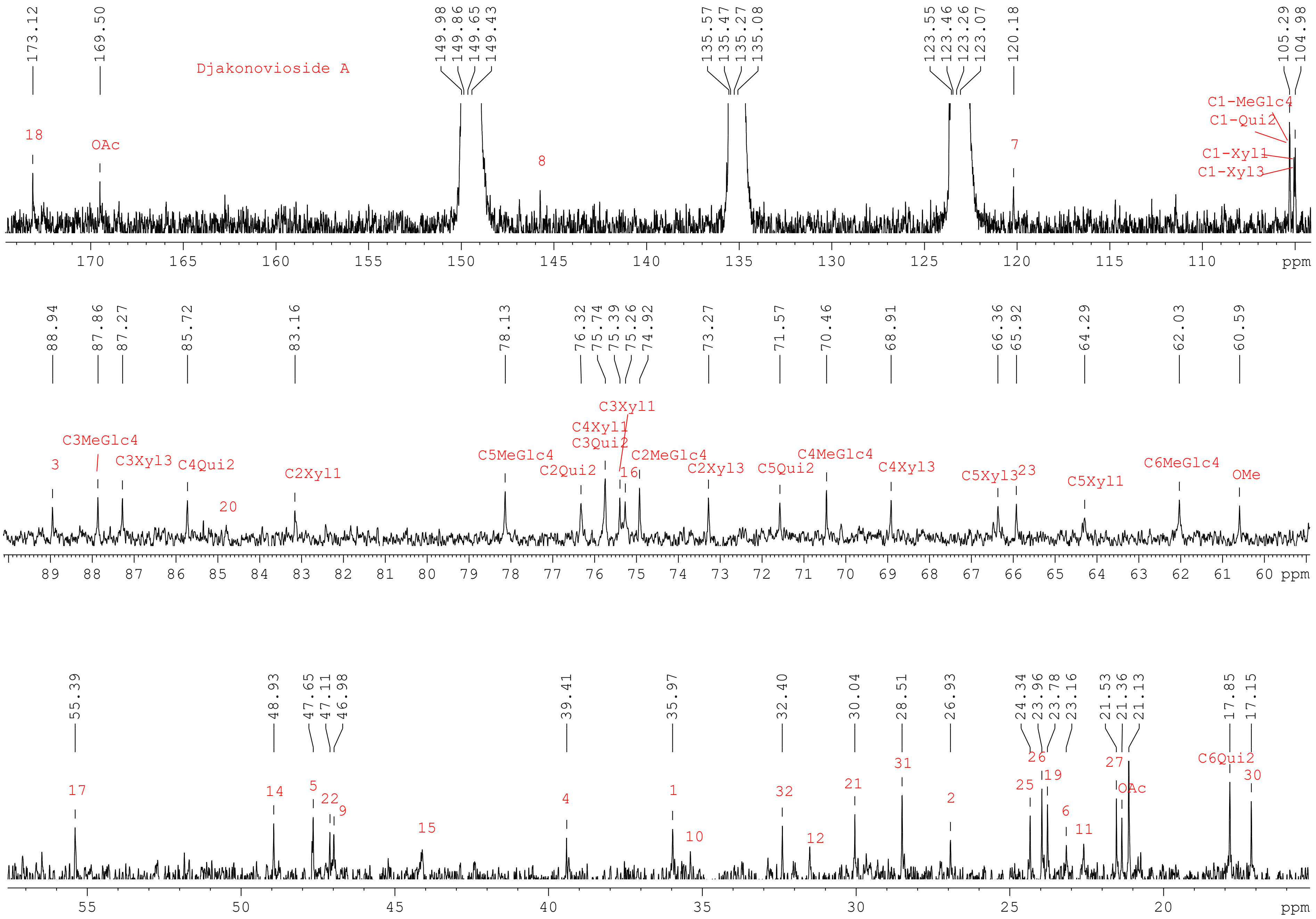

Figure S1. Assigned ^{13}C NMR spectrum of djakonovioside A isolated from *C. conicospermium*

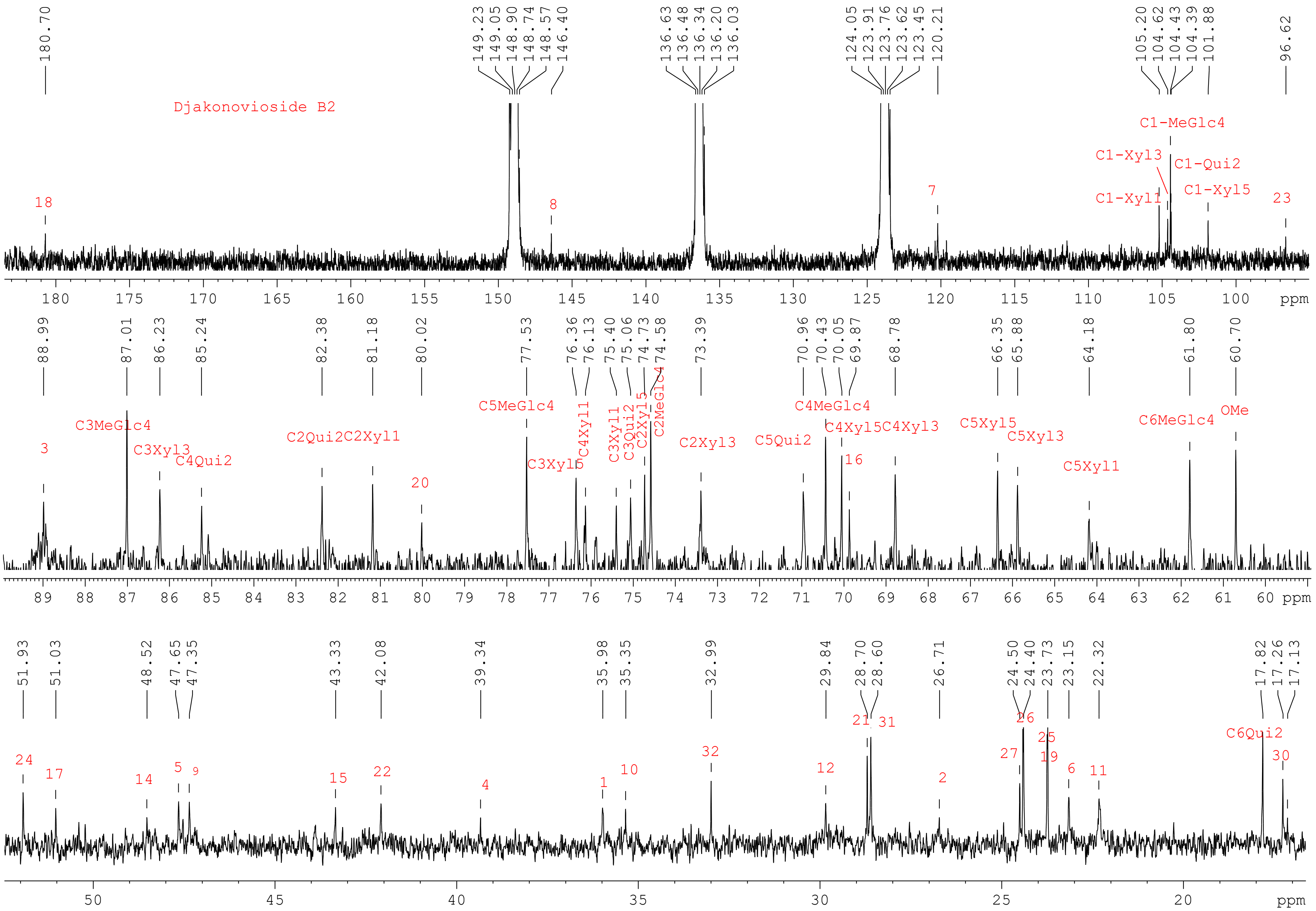

Figure S2. Assigned ^{13}C NMR spectrum of djakonovioside B_2 isolated from *C. conicospermium*

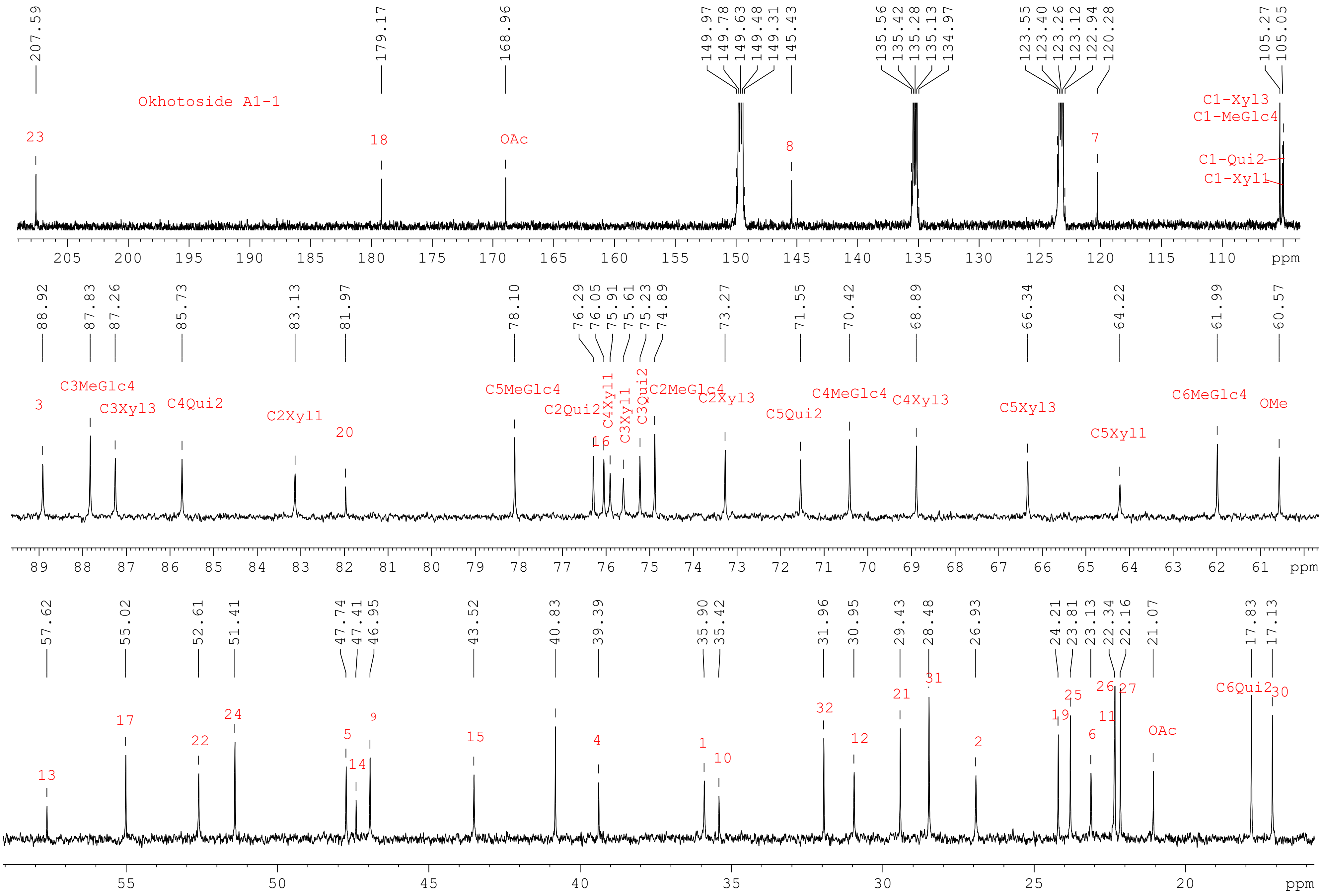

Figure S3. Assigned ^{13}C NMR spectrum of okhotoside A$_1$-1 isolated from *C. conicospermium*

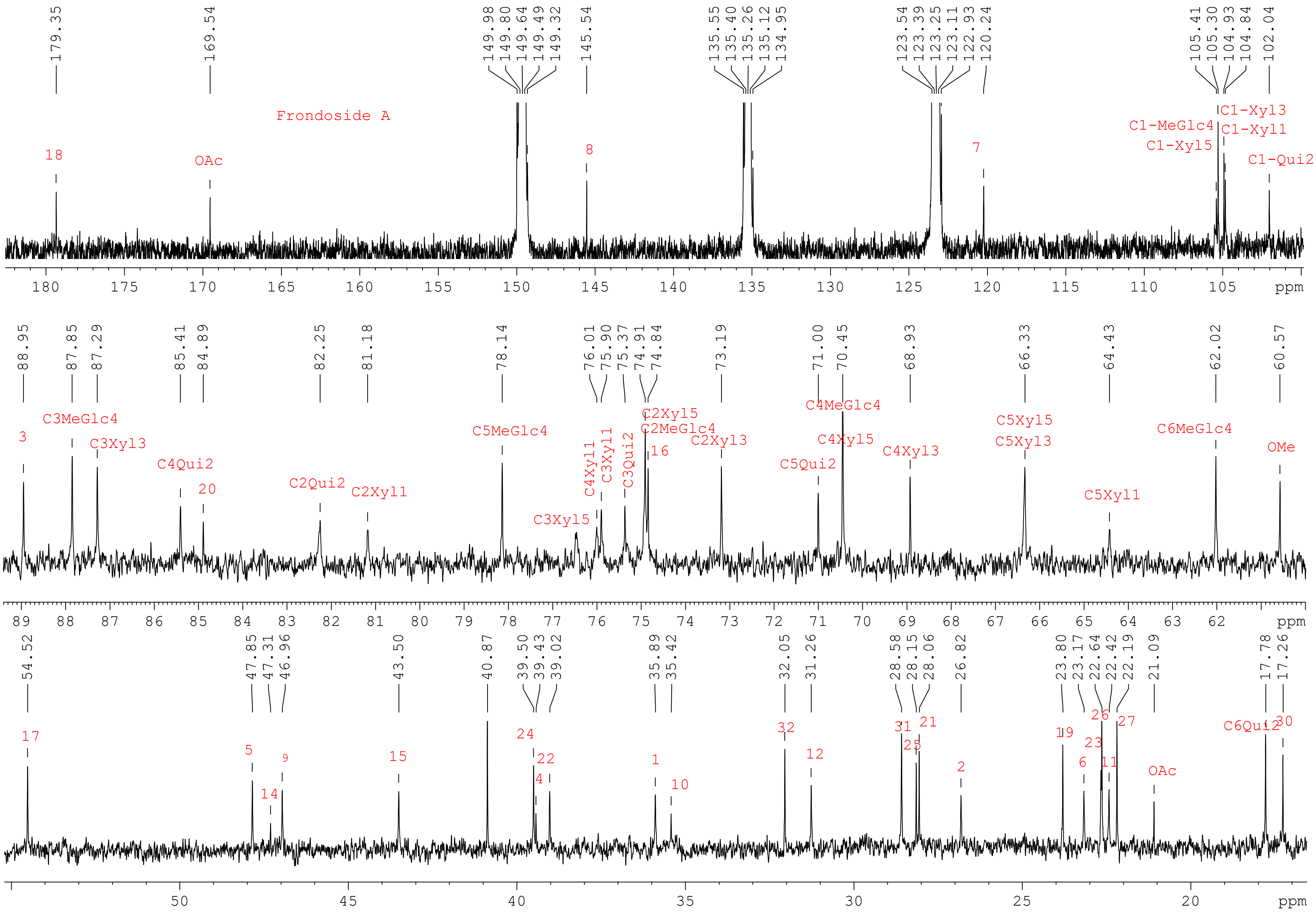

Figure S4. Assigned ^{13}C NMR spectrum of frondoside A isolated from *C. conicospermium*

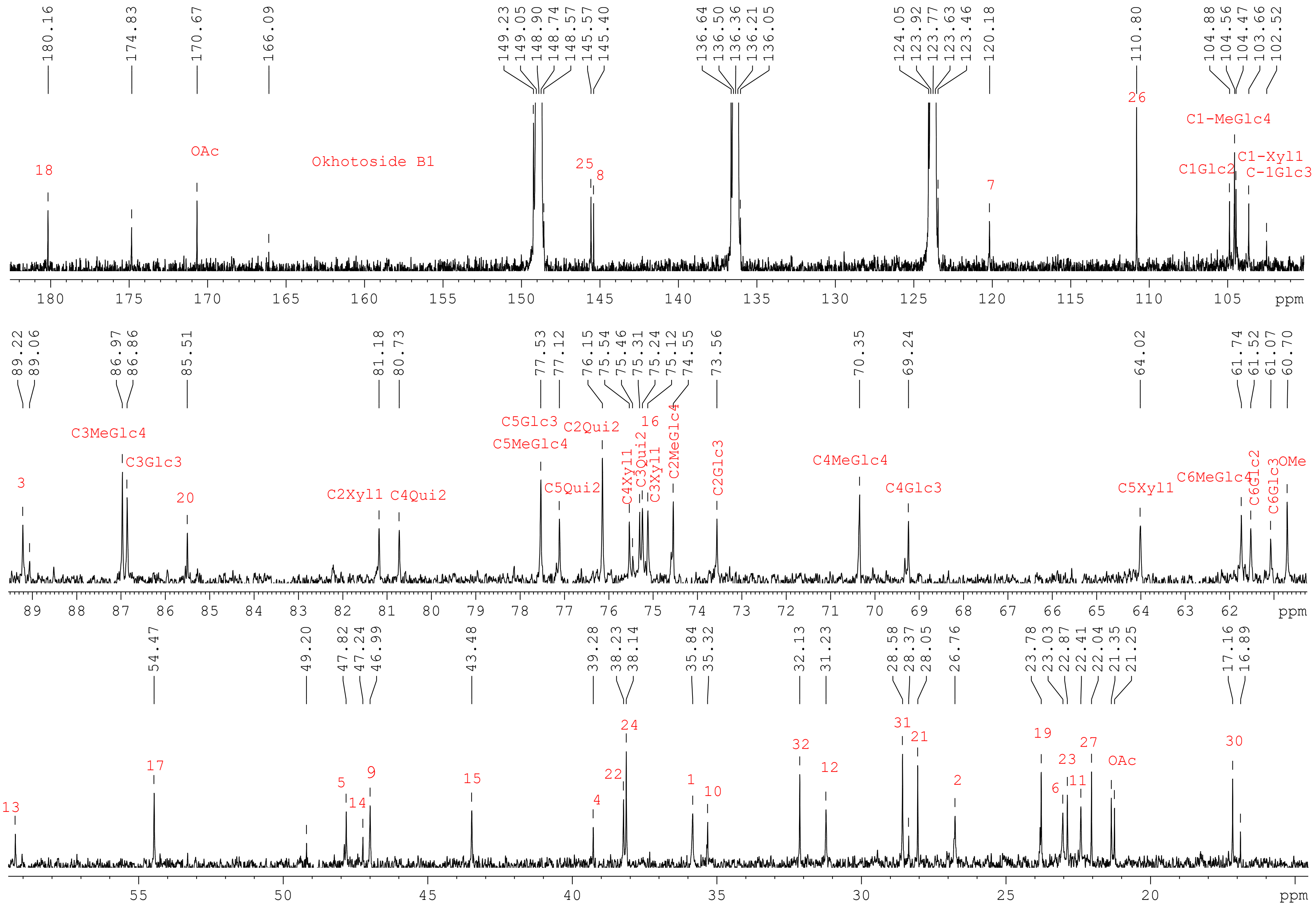

Figure S5. Assigned ^{13}C NMR spectrum of okhotoside B$_1$ isolated from *C. conicospermium*

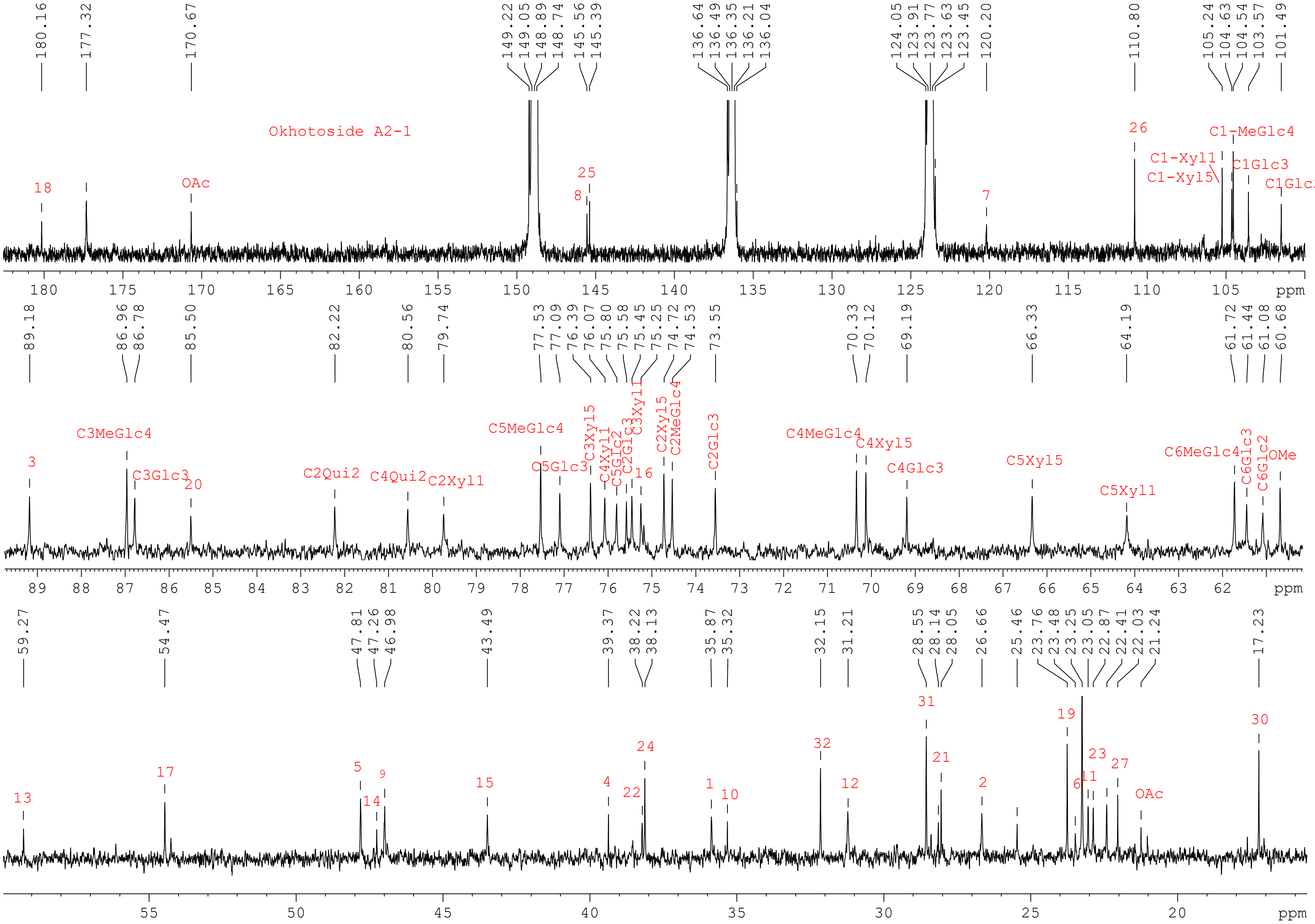

Figure S6. Assigned ^{13}C NMR spectrum of okhotoside A$_2$-1 isolated from *C. conicospermium*

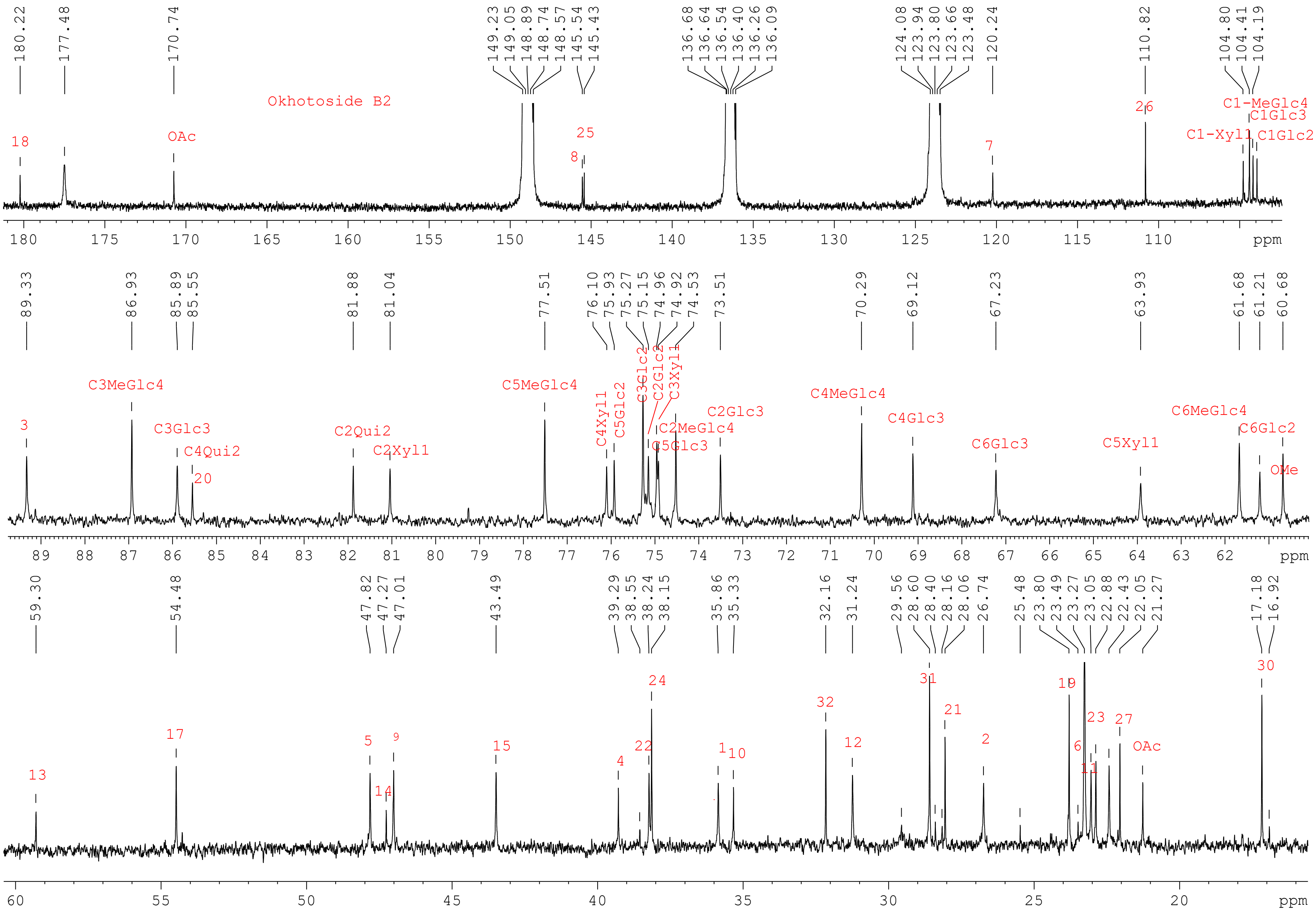

Figure S7. Assigned ^{13}C NMR spectrum of okhotoside B_2 isolated from *C. conicospermium*

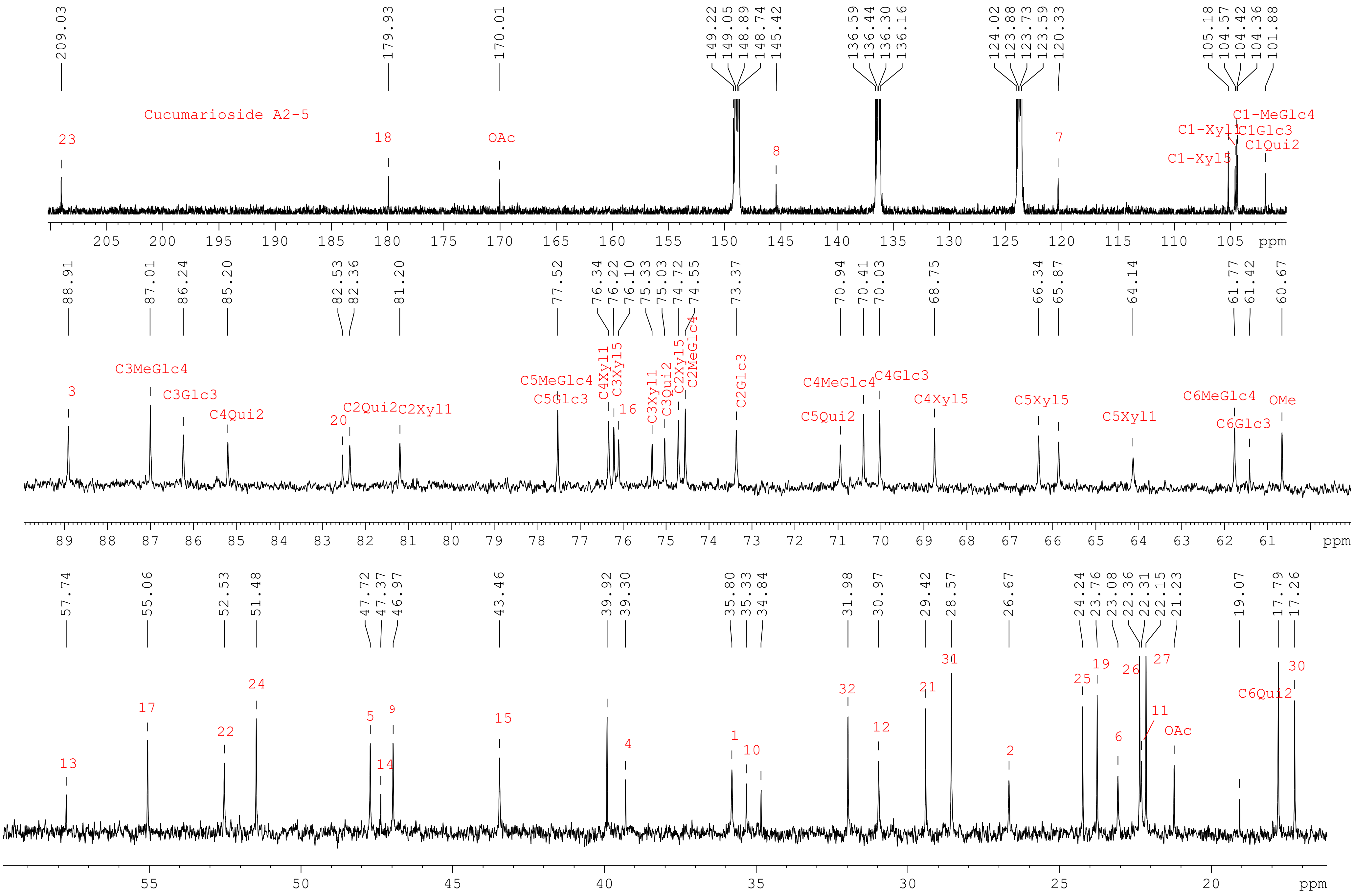

Figure S8. Assigned ^{13}C NMR spectrum of cucumarioside A$_2$-5 isolated from *C. conicospermium*

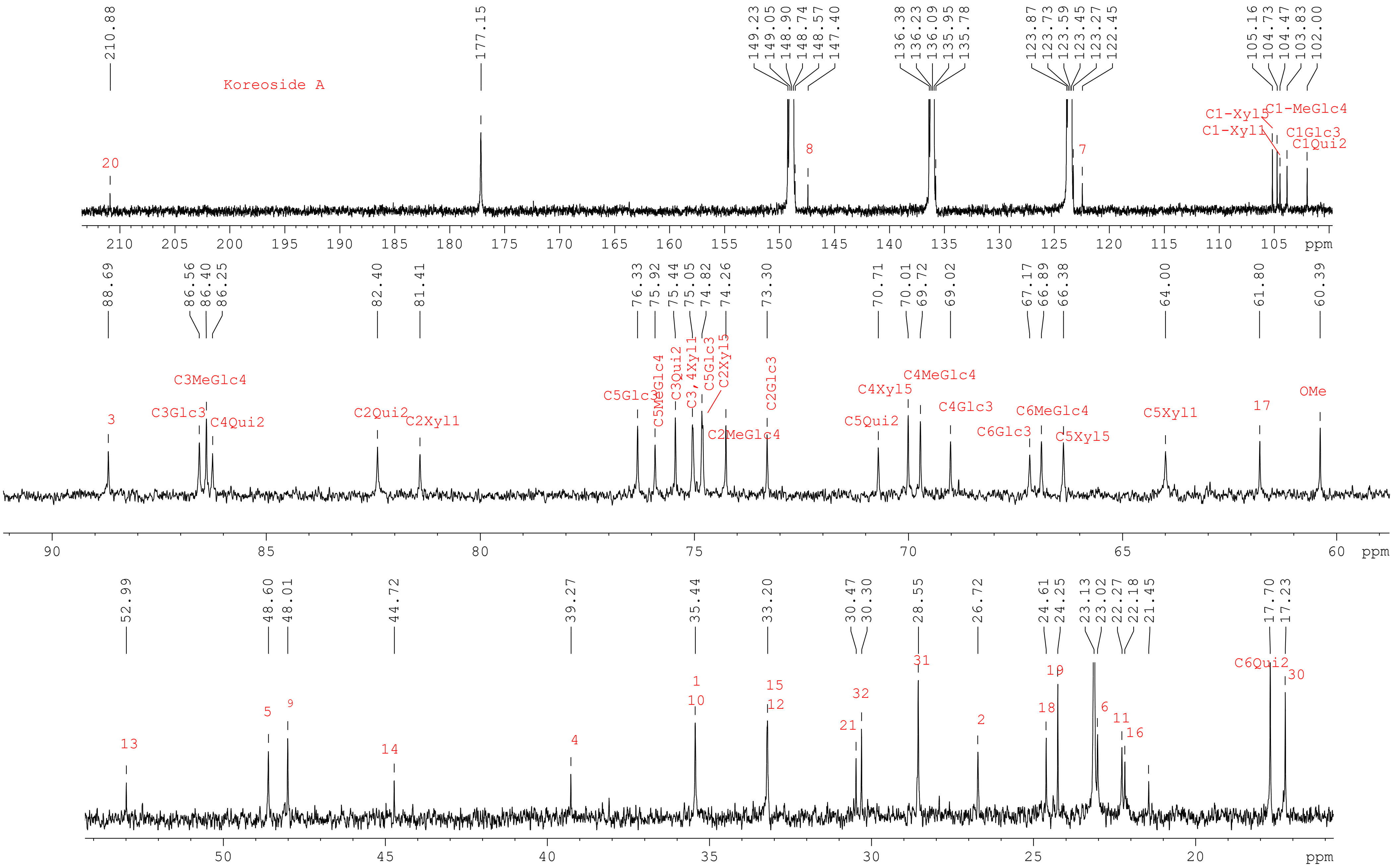

Figure S9. Assigned ^{13}C NMR spectrum of koreoside A isolated from *C. conicospermium*

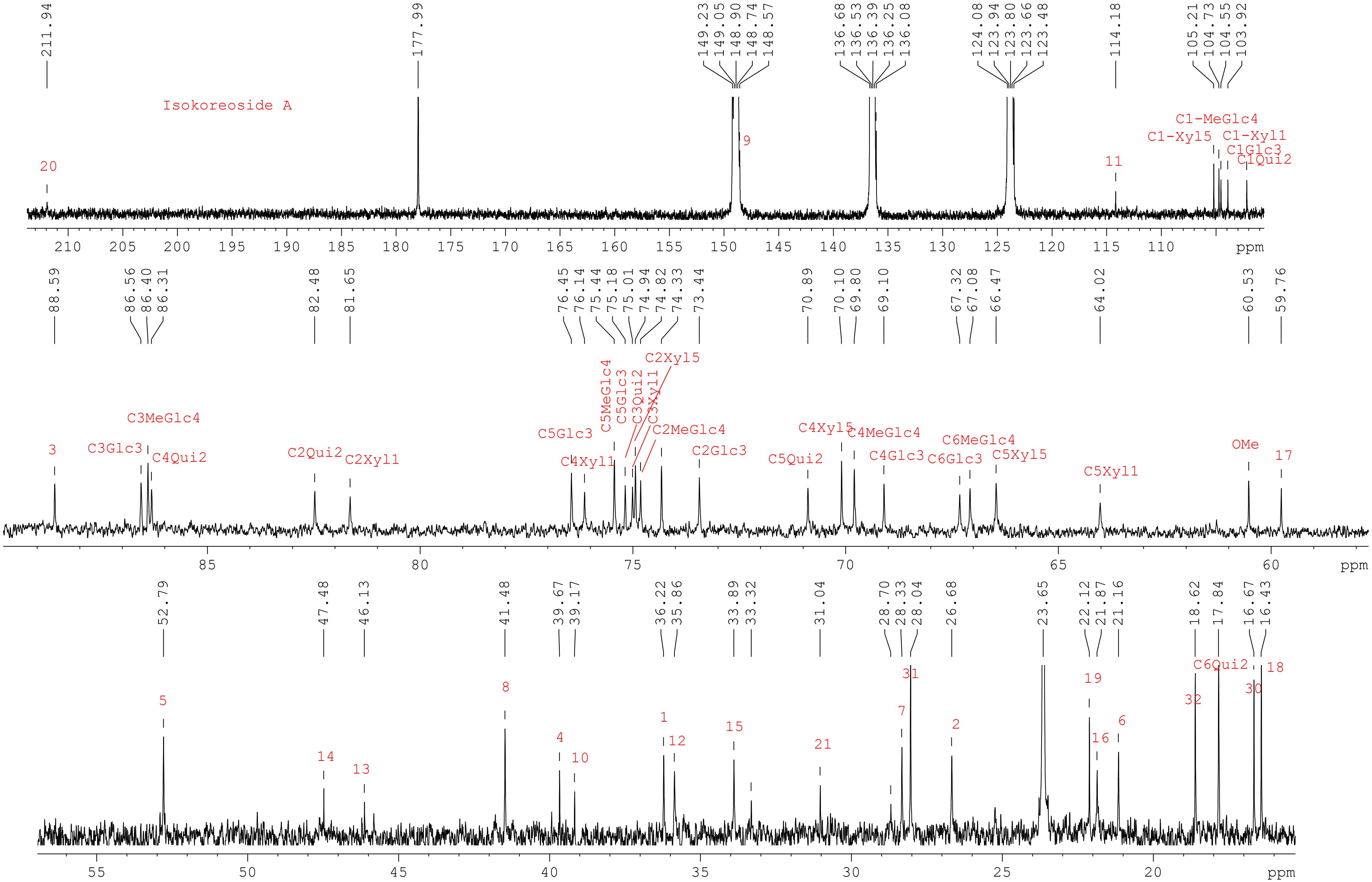

Figure S10. Assigned ^{13}C NMR spectrum of isokoreoside A isolated from *C. conicospermium*

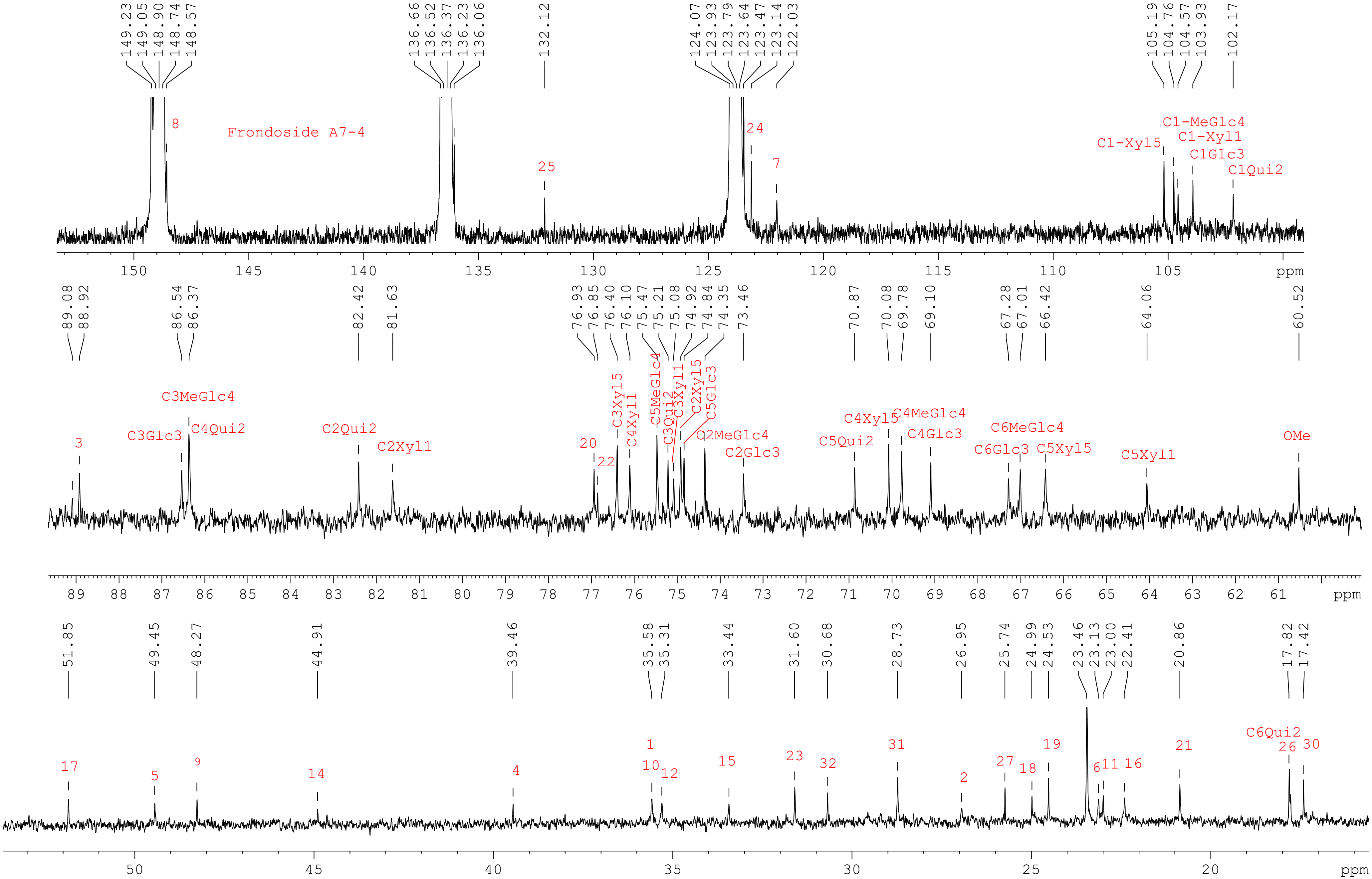

Figure S11. Assigned ^{13}C NMR spectrum of frondoside A7-4 isolated from *C. conicospermium*

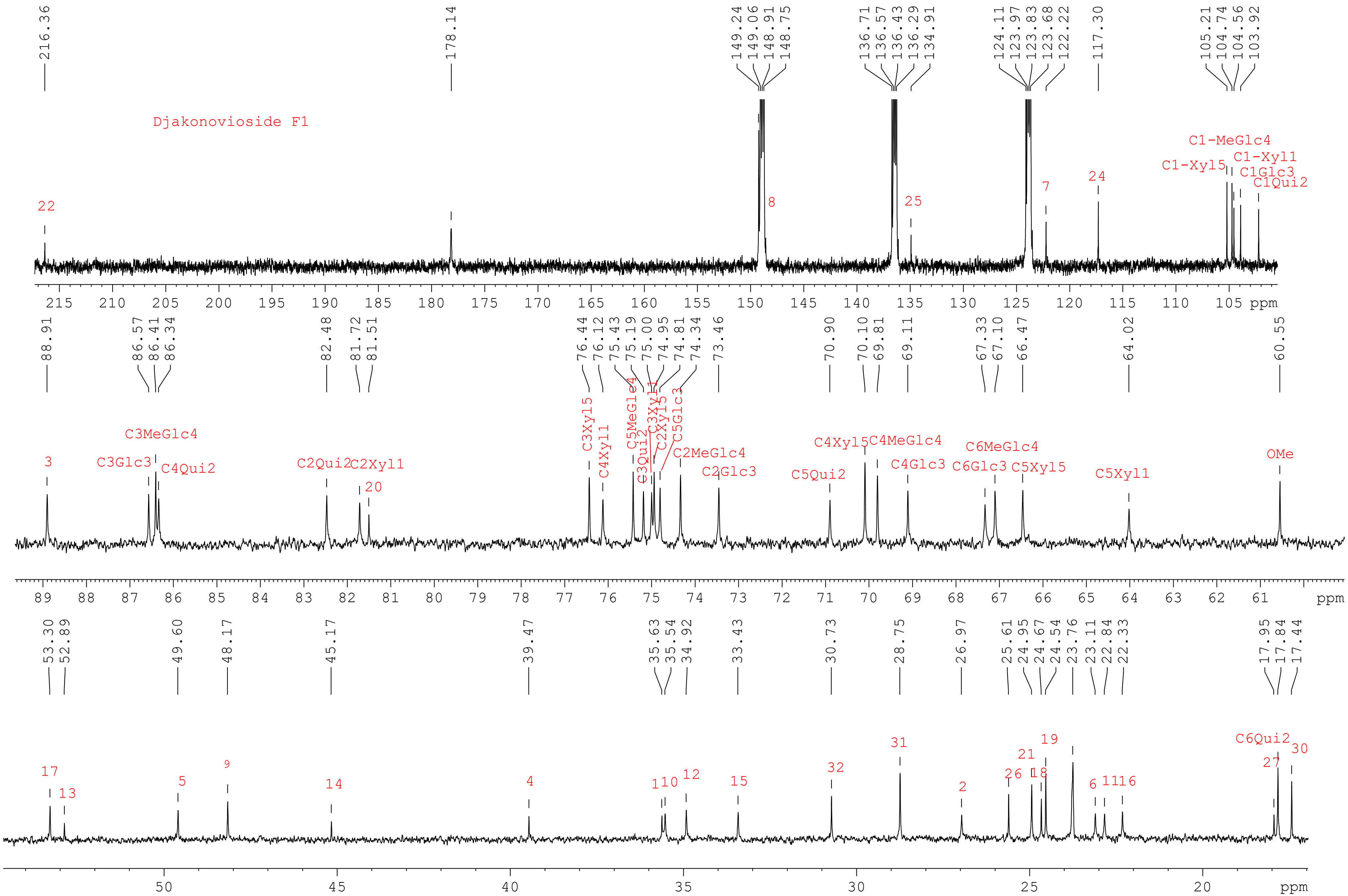

Figure S12. Assigned ^{13}C NMR spectrum of djakonovioside F_1 isolated from *C. conicospermium*

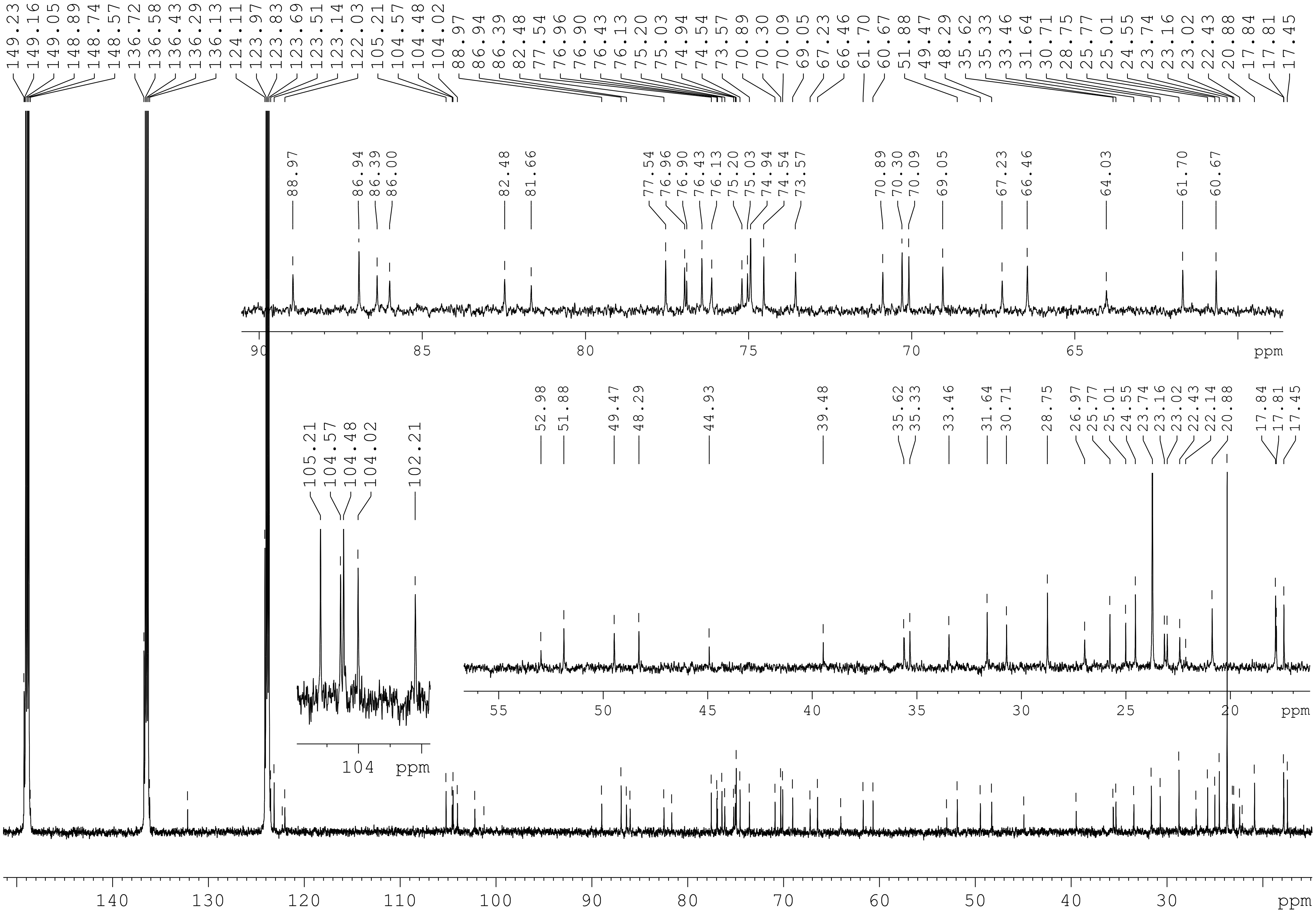

Figure S13. The ^{13}C NMR (125.67 MHz) spectrum of conicospermiumoside A_3-1 (**1**) in C_5D_5N/D_2O (4/1)

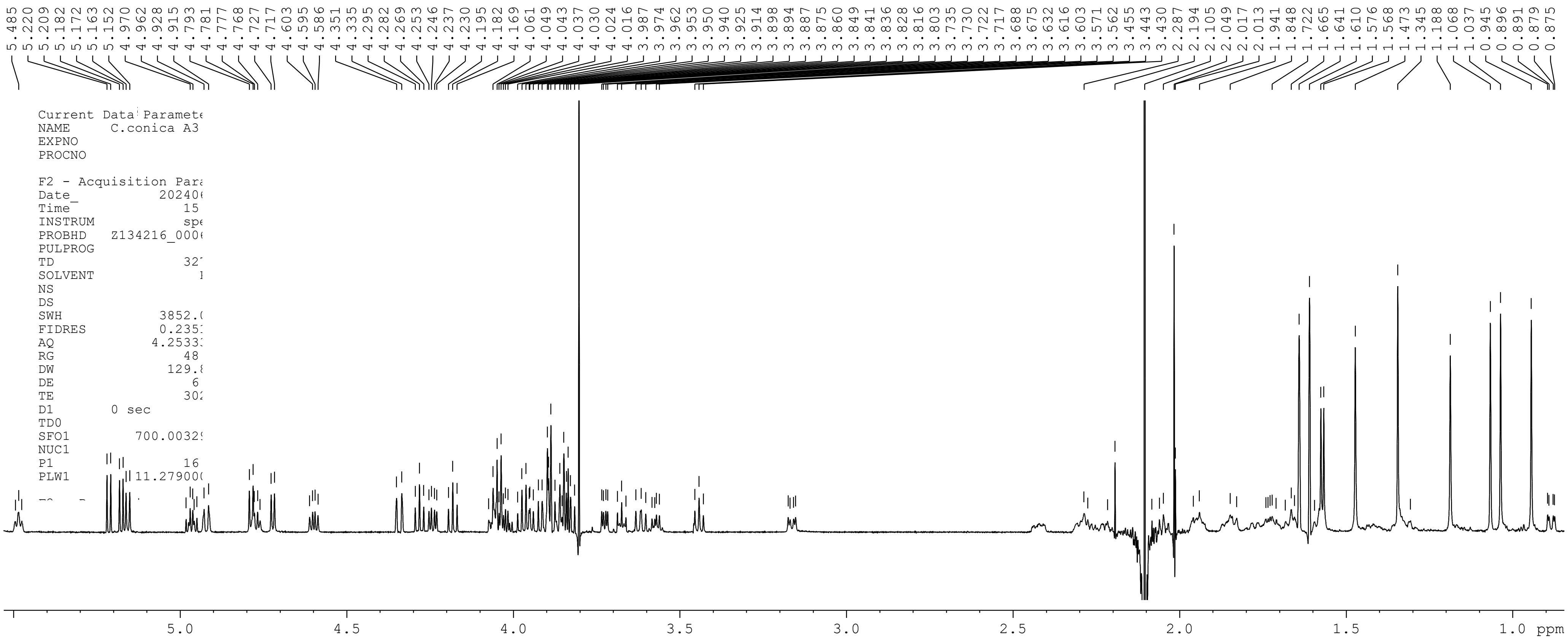

Figure S14. The ^{1}H NMR (700.13 MHz) spectrum of conicospermiumoside A$_3$-1 (**1**) in C_5D_5N/D_2O (4/1)

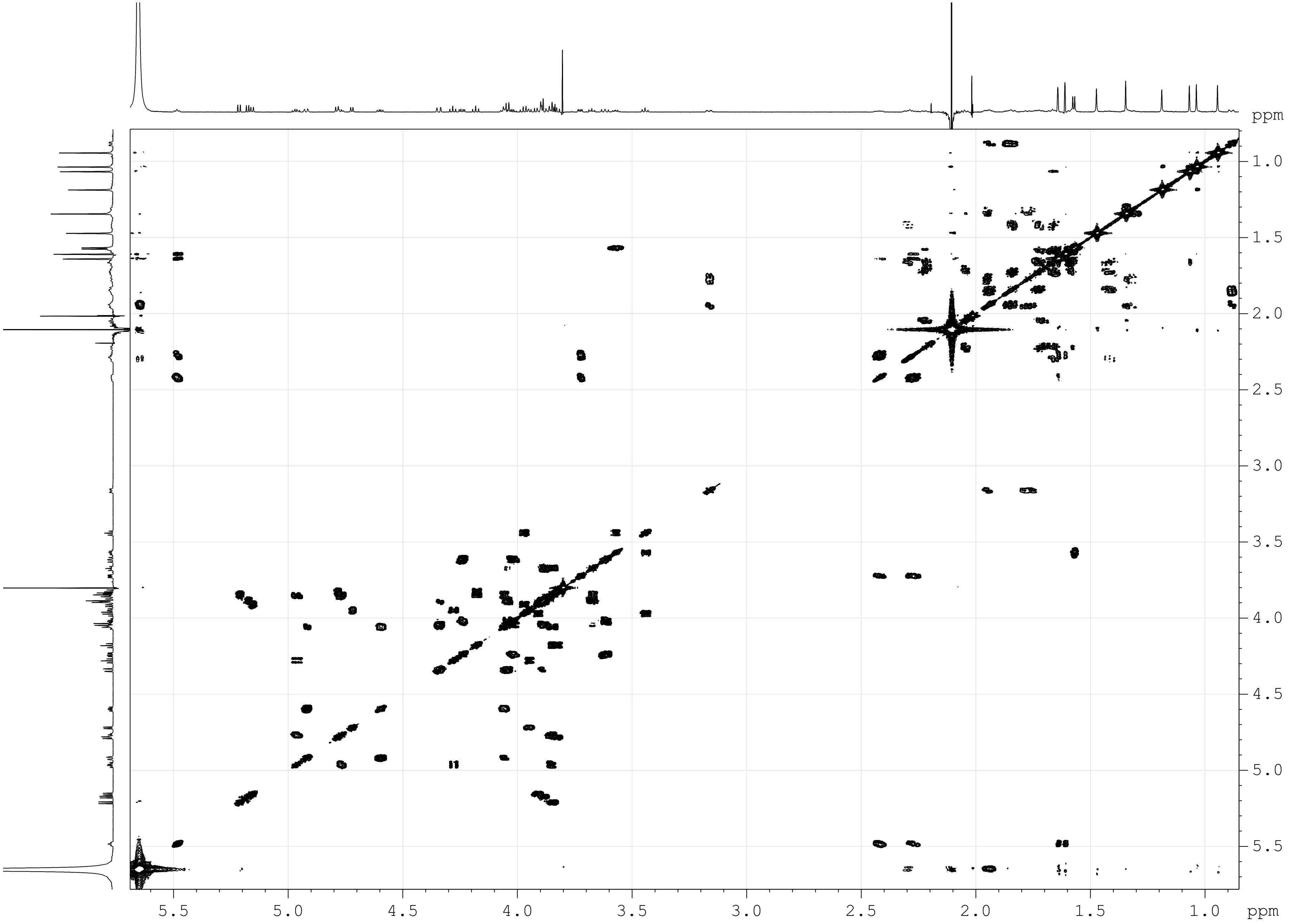

Figure S15. The COSY (700.13 MHz) spectrum of conicospermiumoside A3-1 (**1**) in C_5D_5N/D_2O (4/1)

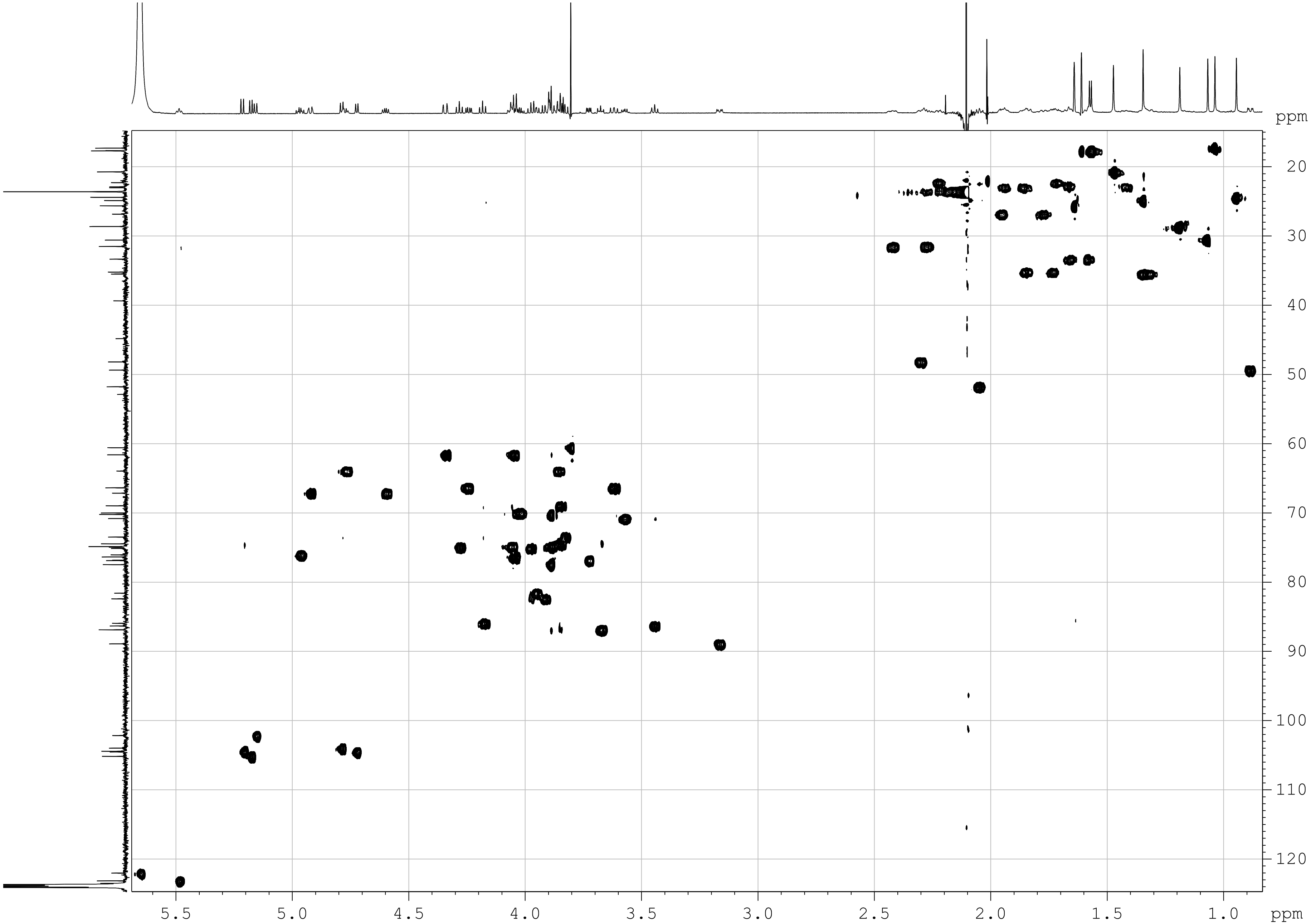

Figure S16. The HSQC (700.13 MHz) spectrum of conicospermiumoside A_3-1 (**1**) in C_5D_5N/D_2O (4/1)

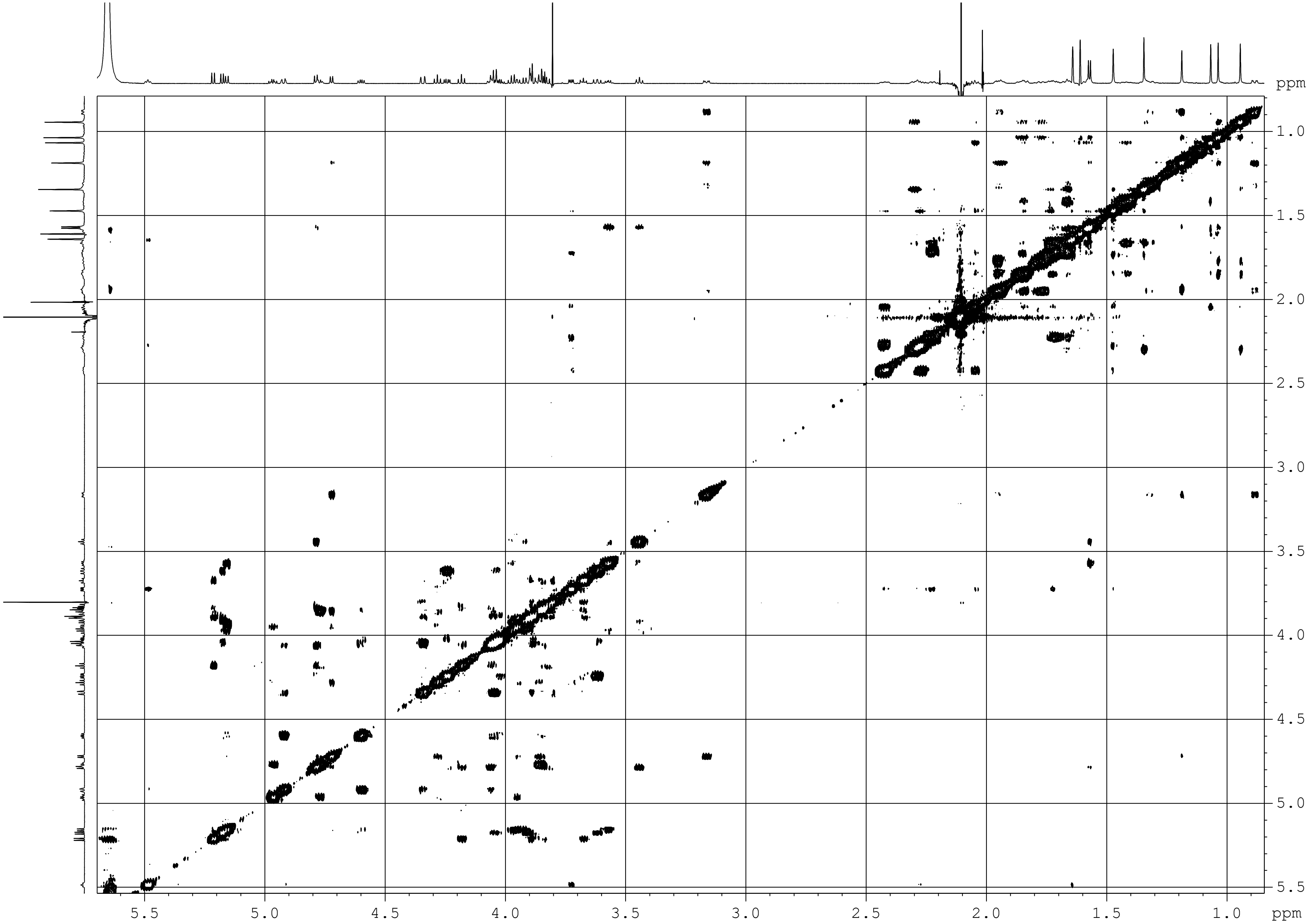

Figure S17. The ROESY (700.13 MHz) spectrum of conicospermiumoside A3-1 (**1**) in C_5D_5N/D_2O (4/1)

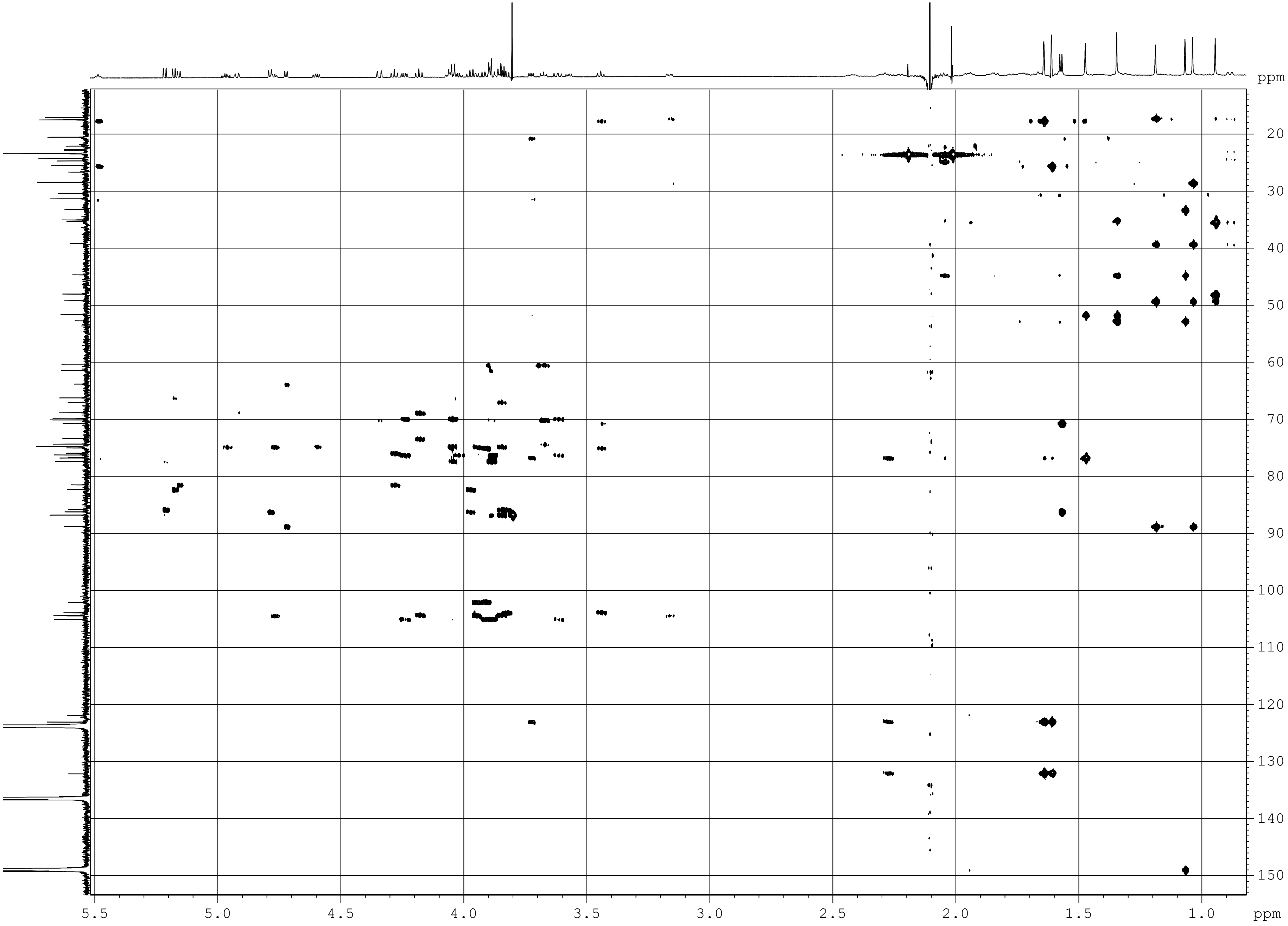

Figure S18. The HMBC (700.13 MHz) spectrum of conicospermiumoside A3-1 (**1**) in C_5D_5N/D_2O (4/1)

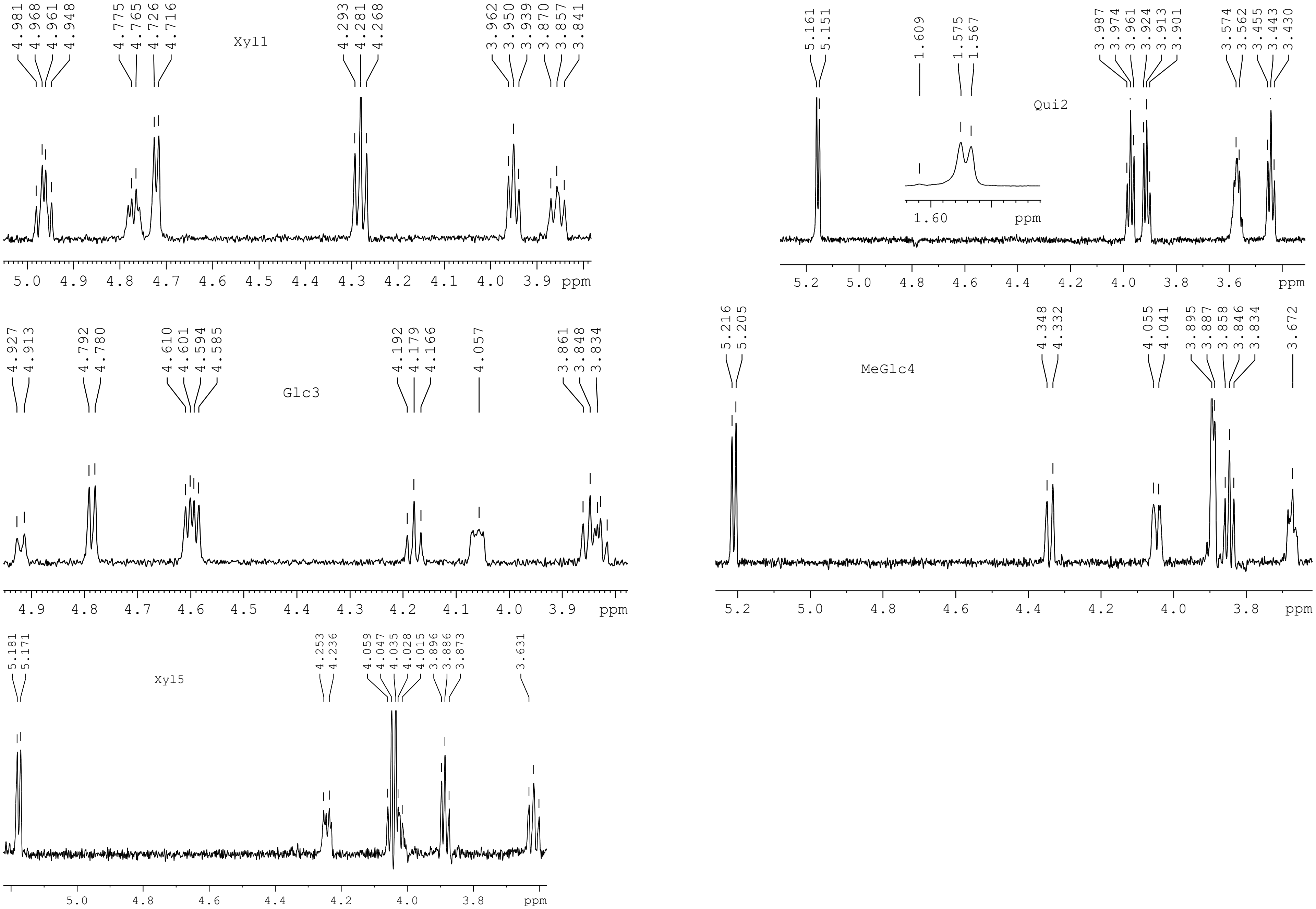

Figure S19. 1 D TOCSY (700.13 MHz) spectra of Xyl1, Qui2, Glc3, MeGlc4, Xyl5 of conicospermiumoside A3-1 (**1**) in C_5D_5N/D_2O (4/1)

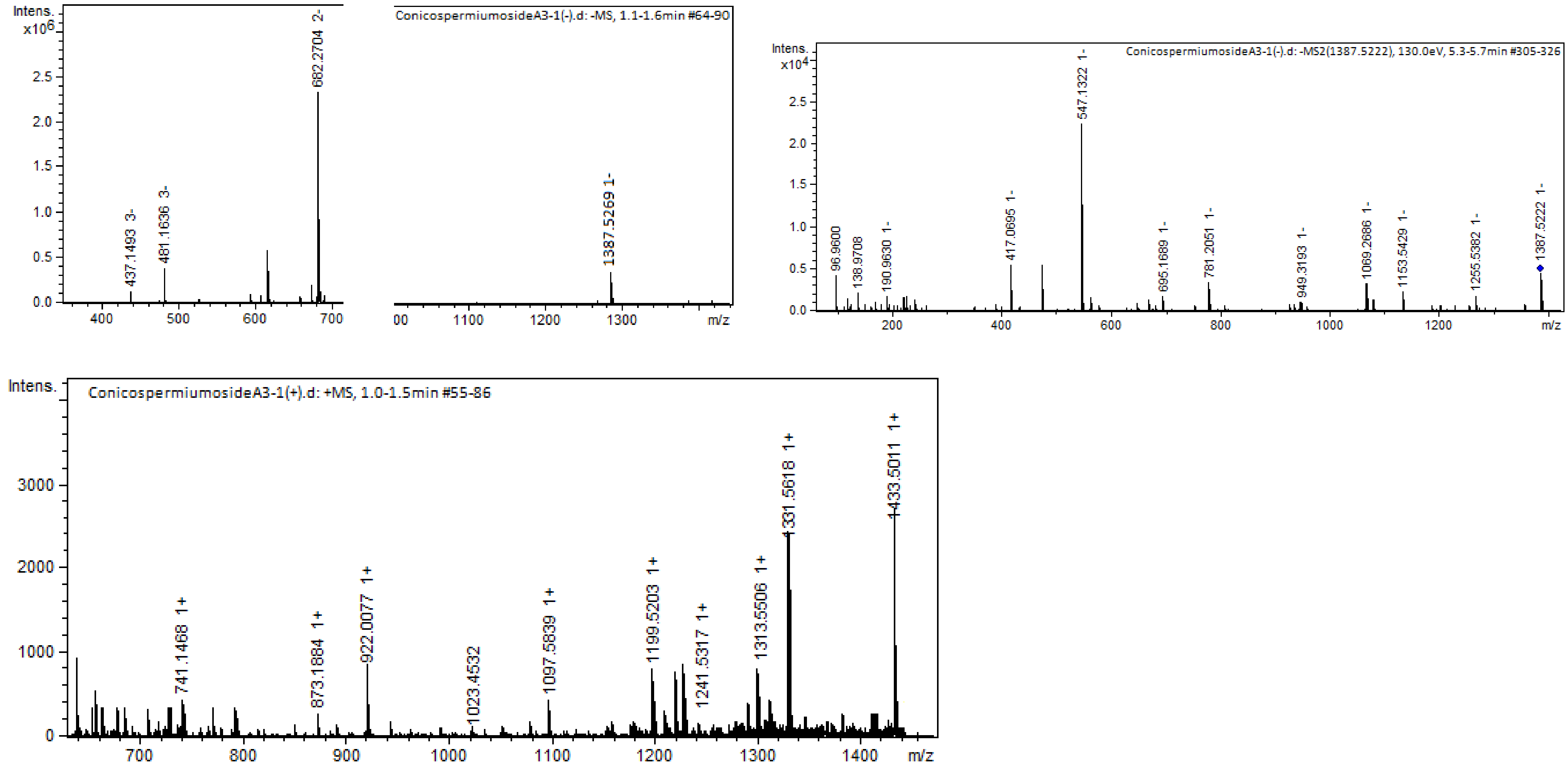

Figure S20. HR-ESI-MS and ESI-MS/MS spectra of conicospermiumoside A3-1 (**1**)

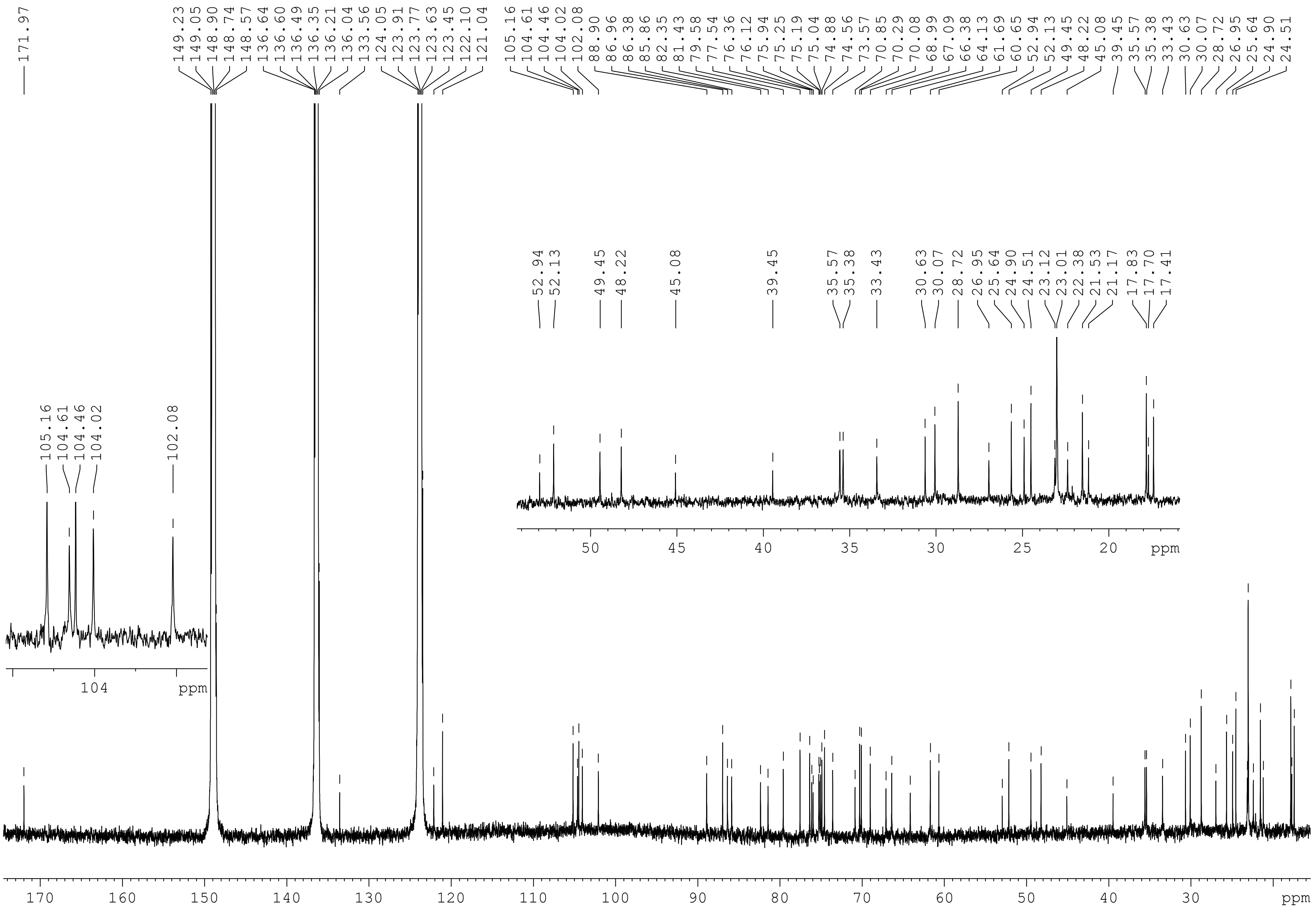

Figure S21. The ^{13}C NMR (125.67 MHz) spectrum of conicospermiumoside A3-2 (**2**) in C_5D_5N/D_2O (4/1)

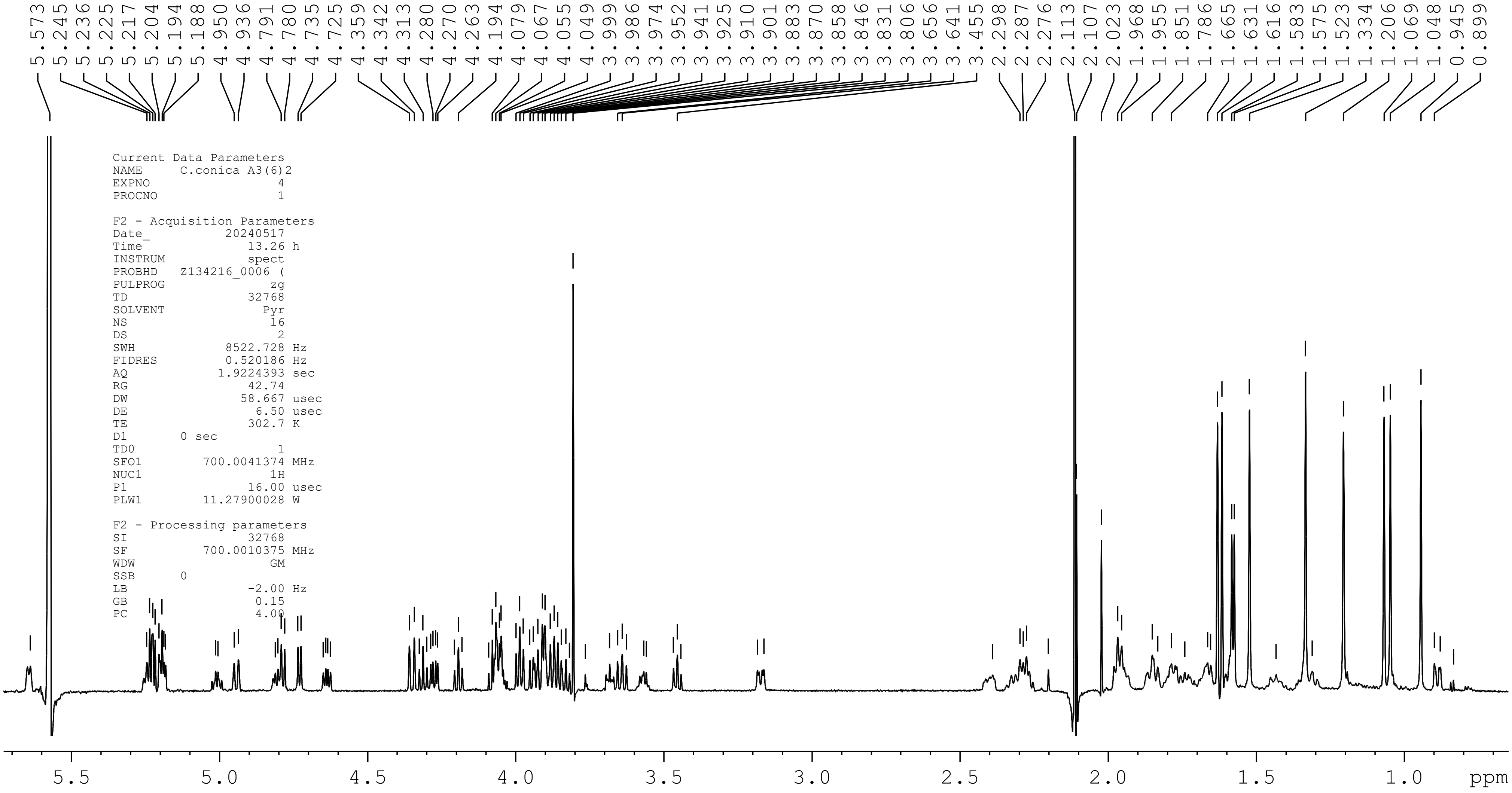

Figure S22. The ^{1}H NMR (700.13 MHz) spectrum of conicospermiumoside A3-2 (**2**) in C_5D_5N/D_2O (4/1)

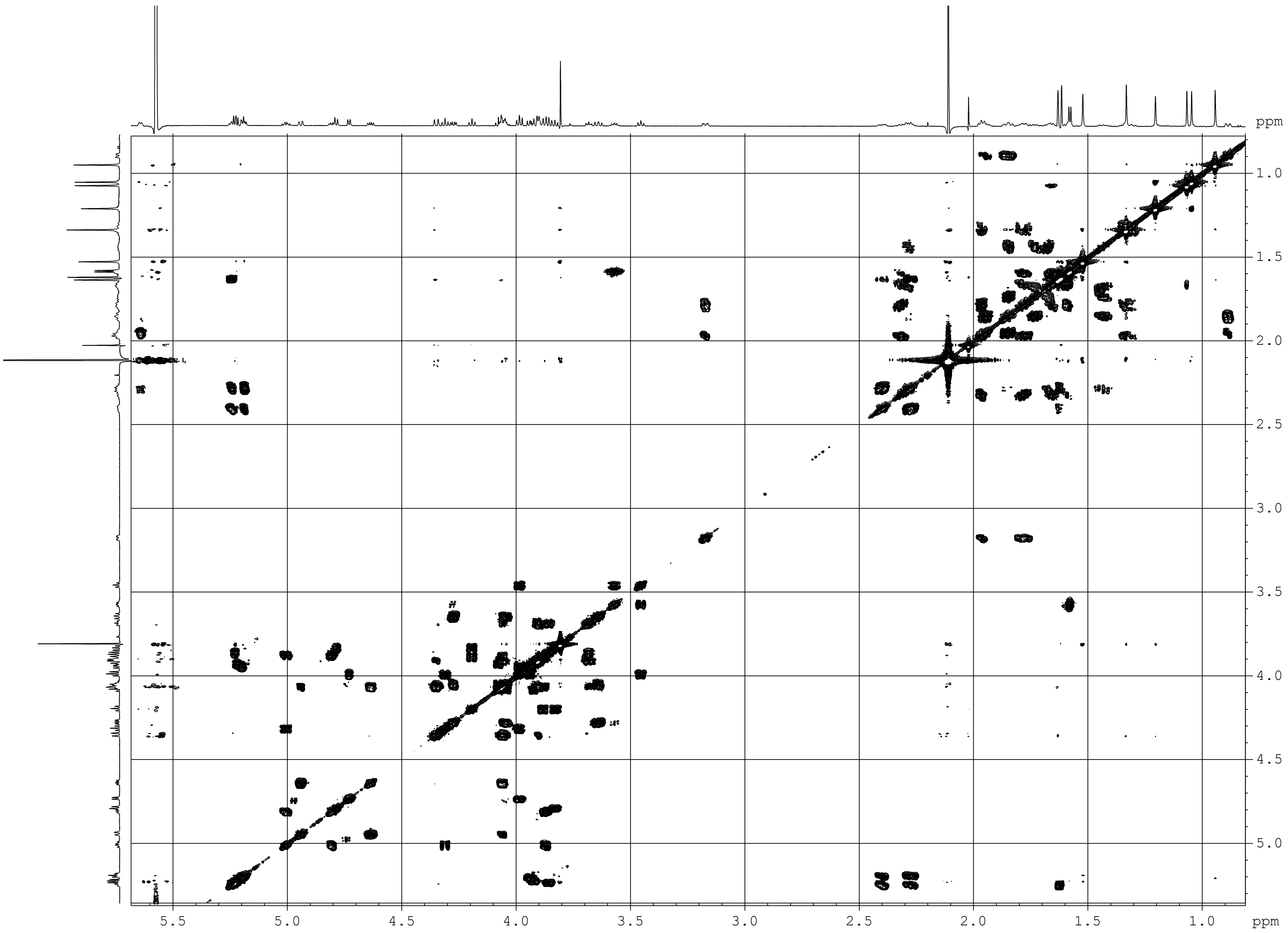

Figure S23. The COSY (700.13 MHz) spectrum of conicospermiumoside A3-2 (**2**) in C_5D_5N/D_2O (4/1)

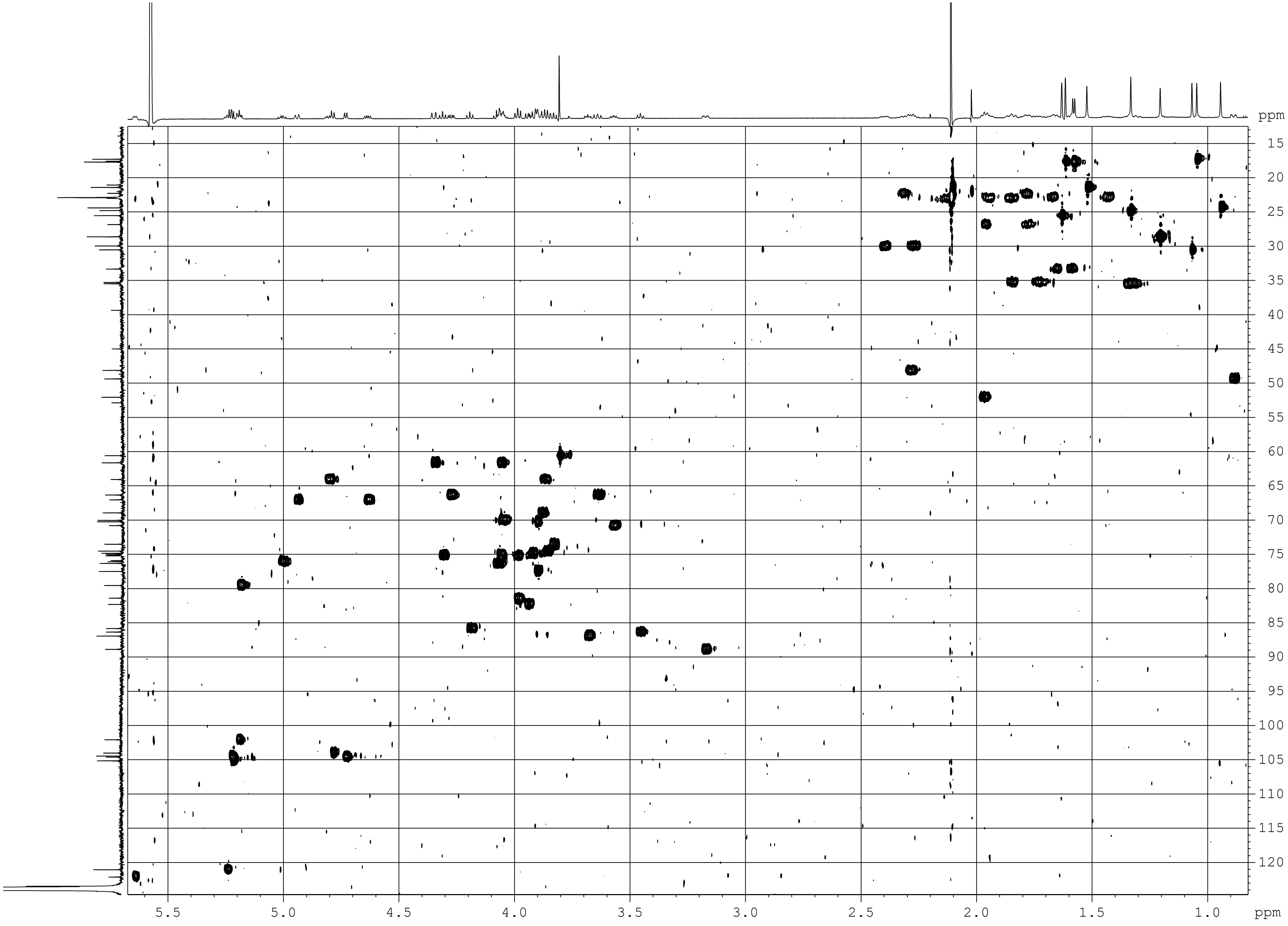

Figure S24. The HSQC (700.13 MHz) spectrum of conicospermiumoside A_3-2 (**2**) in C_5D_5N/D_2O (4/1)

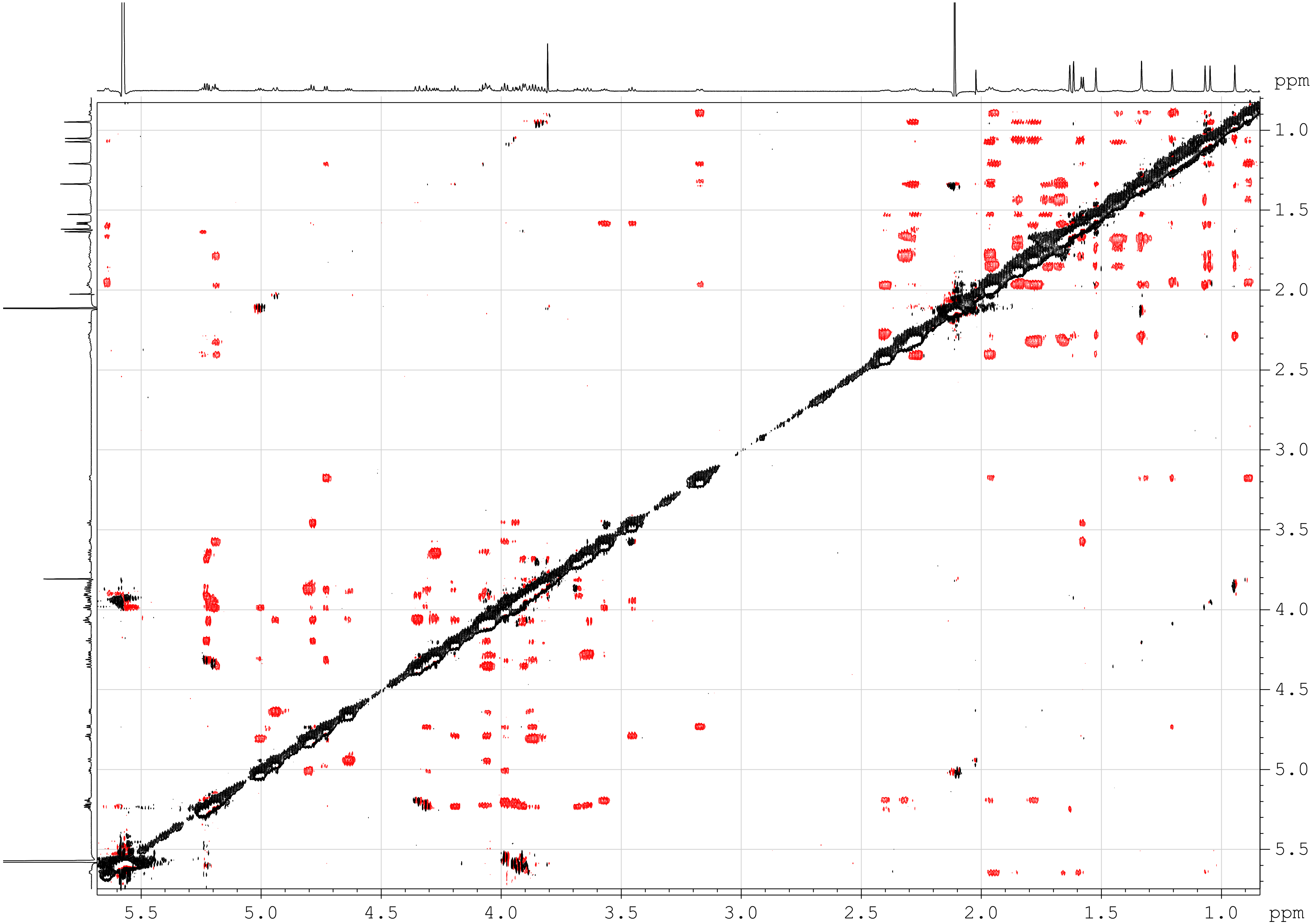

Figure S25. The ROESY (700.13 MHz) spectrum of conicospermiumoside A3-2 (**2**) in C_5D_5N/D_2O (4/1)

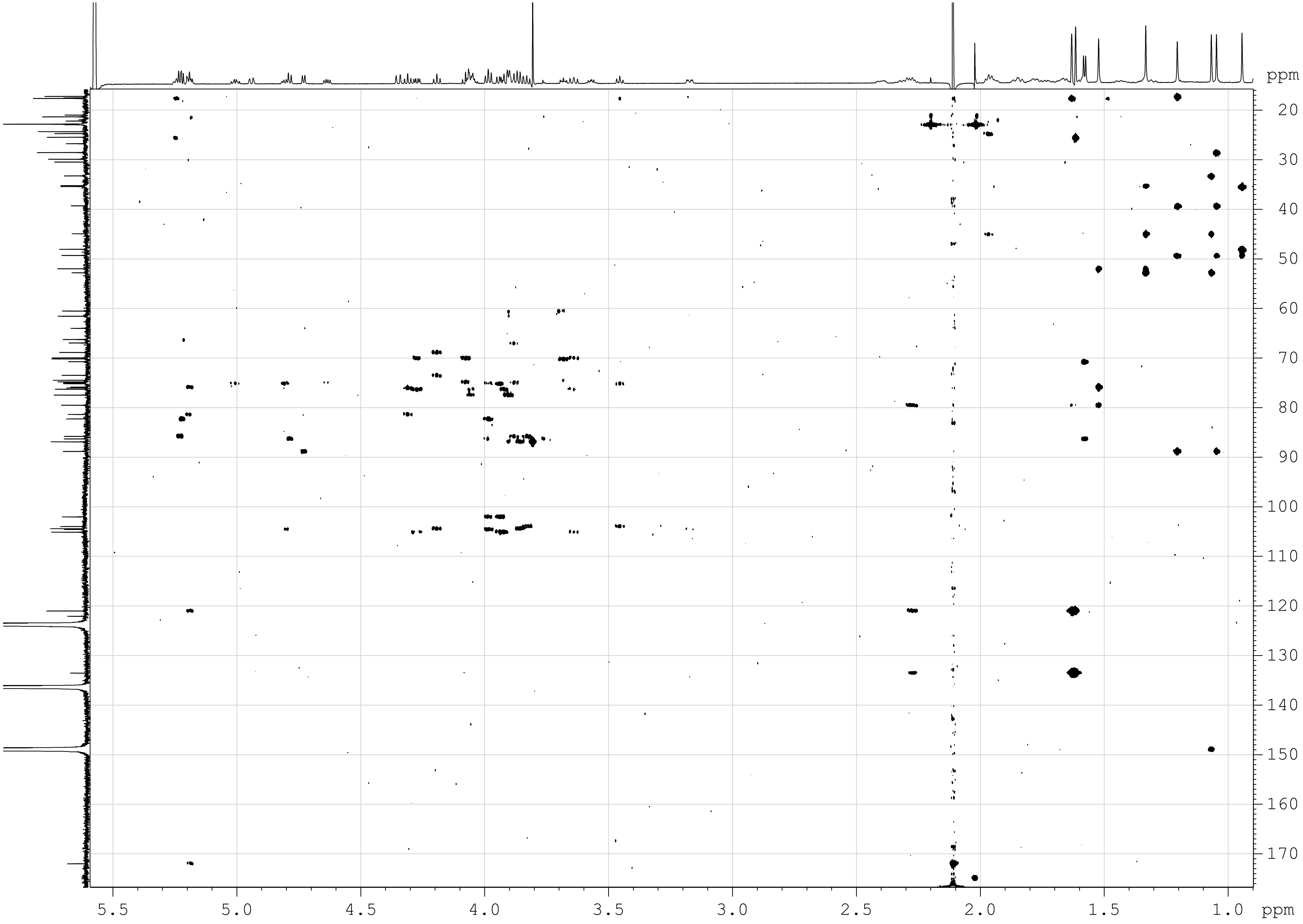

Figure S26. The HMBC (700.13 MHz) spectrum of conicospermiumoside A_3-2 (**2**) in C_5D_5N/D_2O (4/1)

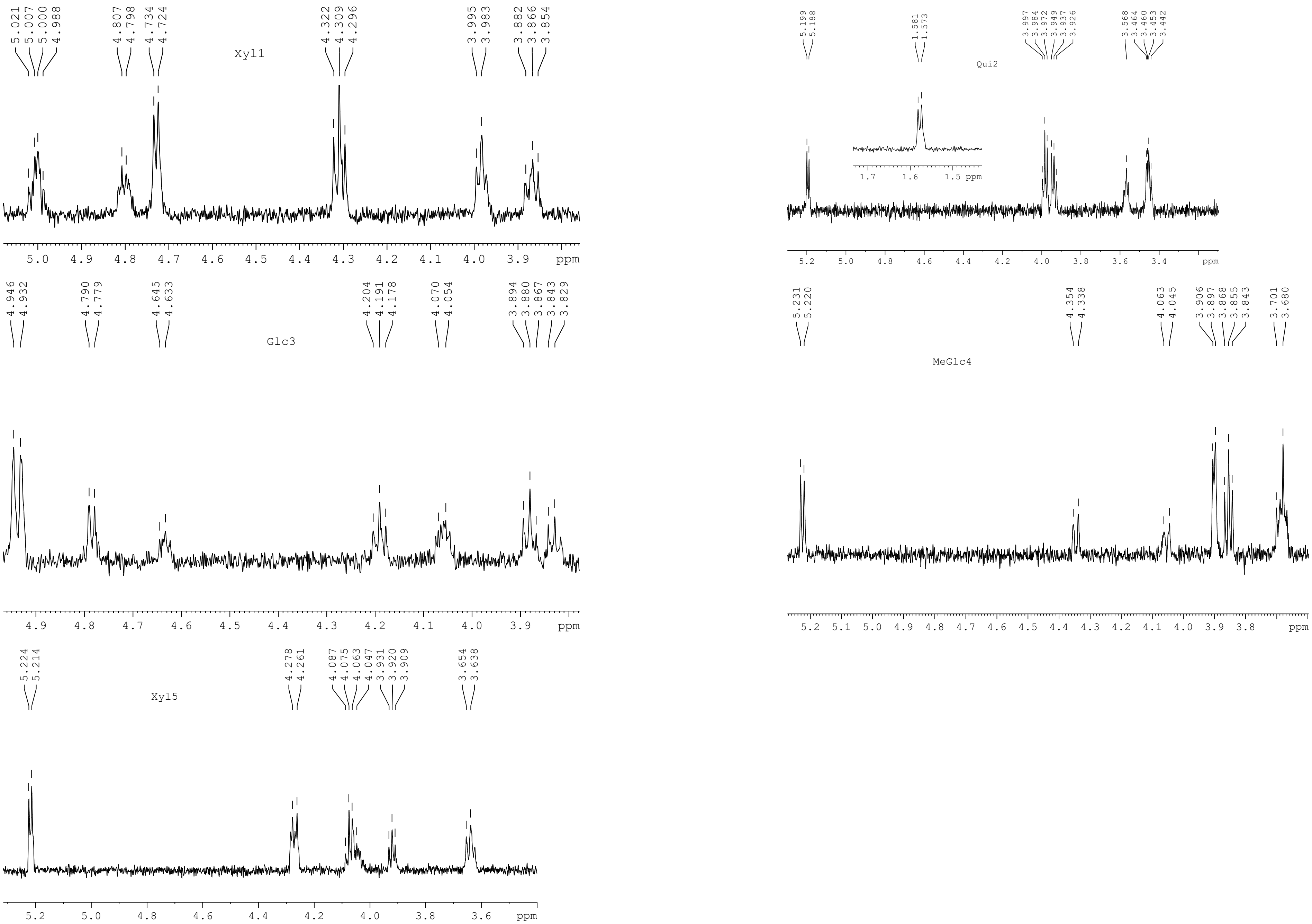

Figure S27. 1 D TOCSY (700.13 MHz) spectra of Xyl1, Qui2, Glc3, MeGlc4, Xyl5 of conicospermiumoside A$_3$-2 (**2**) in C_5D_5N/D_2O (4/1)

Table S1. ^{13}C and ^{1}H NMR chemical shifts and HMBC and ROESY correlations of carbohydrate moiety of conicospermiumoside A$_3$-2 (**2**).

Atom	δ_C mult.[a]	δ_H mult. (*J* in	HMBC	ROESY
Xyl1 (1→C-3)				
1	104.6 CH	4.73 d (6.8)	C: 3; C: 1 Xyl1	H-3; H-3, 5 Xyl1
2	**81.4** CH	3.98 t (8.2)	C: 1 Qui2; C: 1, 3 Xyl1	H-1 Qui2; H-4 Xyl1
3	75.2 CH	4.31 t (8.2)	C: 2, 4 Xyl1	H-1, 5 Xyl1
4	*76.1* CH	5.00 m	C: 3 Xyl1	H-2 Xyl1
5	64.1 CH_2	4.80 m	C: 1, 3 Xyl1	
		3.87 t (10.9)		H-1, 3 Xyl1
Qui2 (1→2Xyl1)				
1	102.1 CH	5.19 d (7.0)	C: 2 Xyl1	H-2 Xyl1; H-3, 5 Qui2
2	**82.4** CH	3.94 t (9.3)	C: 1, 3 Qui2; C: 1 Xyl5	H-4 Qui2; H-1 Xyl5
3	75.3 CH	3.98 t (9.3)	C: 2, 4 Qui2	
4	**86.4** CH	3.45 t (9.3)	C: 3 Qui2; C: 1 Glc3	H-1 Glc3; H-2 Qui2
5	70.8 CH	3.57 dd (6.8; 9.3)		H-1, 3 Qui2
6	17.7 CH_3	1.58 d (6.8)	C: 4, 5 Qui2	
Glc3 (1→4Qui2)				
1	104.0 CH	4.78 d (8.4)	C: 4 Qui2	H-4 Qui2; H-3, 5 Glc3
2	73.6 CH	3.83 t (8.6)	C: 1, 3 Glc3	
3	**85.9** CH	4.19 t (8.6)	C: 2, 4 Glc3, C: 1 MeGlc4	H-1 MeGlc4; H-1, 5 Glc3
4	69.0 CH	3.88 t (8.6)	C: 3, 5, 6 Glc3	H-6 Glc3
5	75.0 CH	4.06 m	C: 1, 3 Xyl3	H-1, 3 Glc3
6	67.1 CH_2	4.94 d (11.7)		
		4.64 t (9.3)	C: 5 Glc3	H-4 Glc3
MeGlc4 (1→3Glc3)				
1	104.5 CH	5.23 d (7.2)	C: 3 Glc3	H-3 Glc3; H-3, 5 MeGlc4
2	74.6 CH	3.85 t (8.2)	C: 1, 3 MeGlc4	
3	87.0 CH	3.68 t (8.2)	C: 2, 4 MeGlc4; OMe	H-1, 5 MeGlc4
4	70.3 CH	3.90 m	C: 3, 5 MeGlc4	
5	77.5 CH	3.90 m		H-1 MeGlc4
6	61.7 CH_2	4.35 d (11.9)		
		4.06 brd (11.9)	C: 5 MeGlc4	
OMe	60.6 CH_3	3.80 s	C: 3 MeGlc4	
Xyl5 (1→2Qui2)				
1	105.2 CH	5.22 d (7.0)	C: 2 Qui2	H-2 Qui2; H-3, 5 Xyl5
2	74.9 CH	3.92 t (7.8)	C: 1, 3 Xyl5	
3	76.4 CH	4.07 t (7.8)	C: 2, 4 Xyl5	
4	70.1 CH	4.05 m	C: 3 Xyl5	
5	66.4 CH_2	4.27 dd (5.4; 11.6)	C: 1, 3, 4 Xyl5	
		3.64 brt (10.9)	C: 1, 3, 4 Xyl5	H-1 Xyl5

[a] Recorded at 176.04 MHz in C_5D_5N/D_2O. [b] Bold—interglycosidic positions. [c] Italics—sulfate position. [d] Recorded at 700.13 MHz in C_5D_5N/D_2O. Multiplicity by 1D TOCSY.

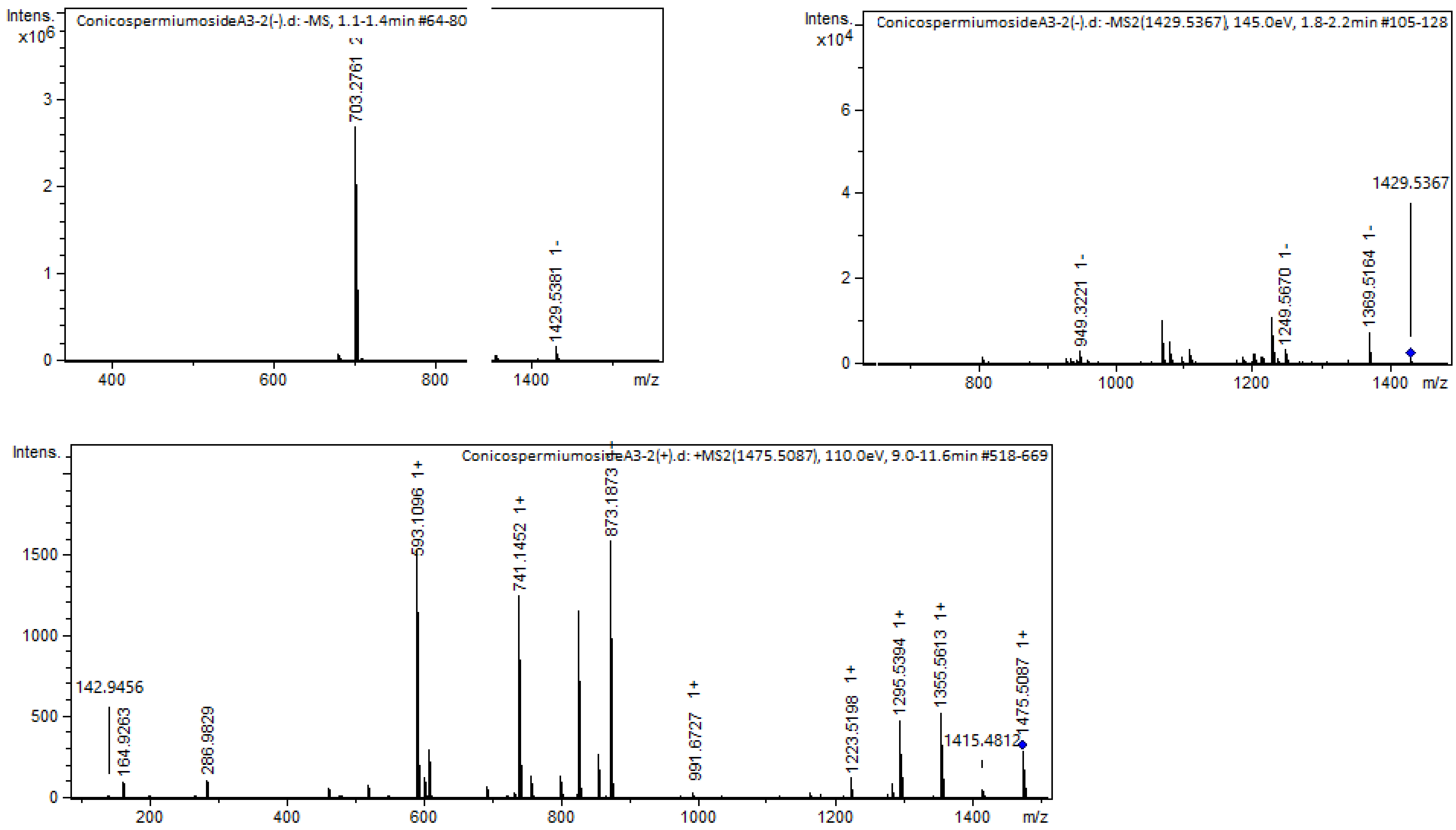

Figure S28. HR-ESI-MS and ESI-MS/MS spectra of conicospermiumoside A3-2 (**2**)

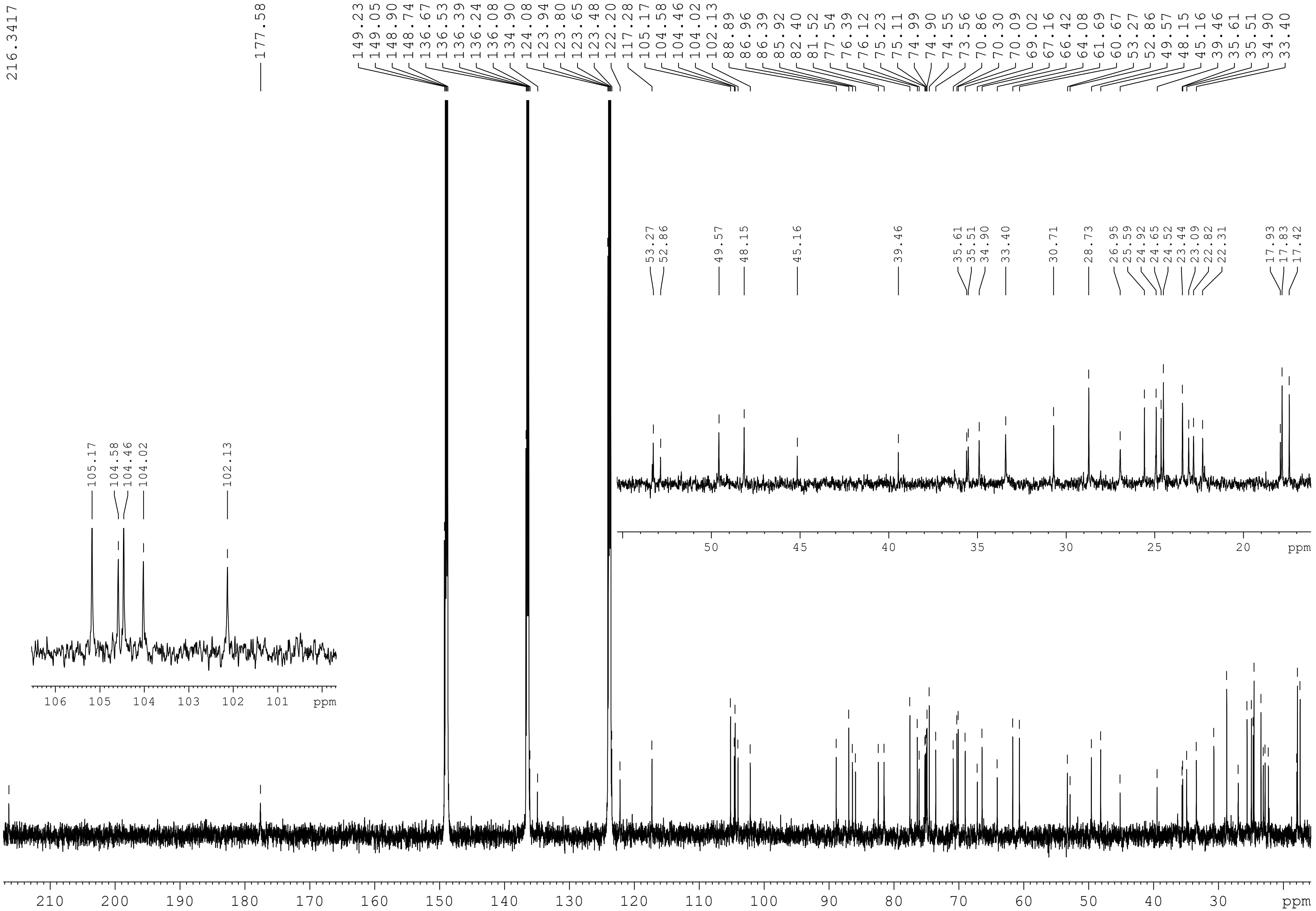

Figure S29. The ^{13}C NMR (125.67 MHz) spectrum of conicospermiumoside A3-3 (**3**) in C_5D_5N/D_2O (4/1)

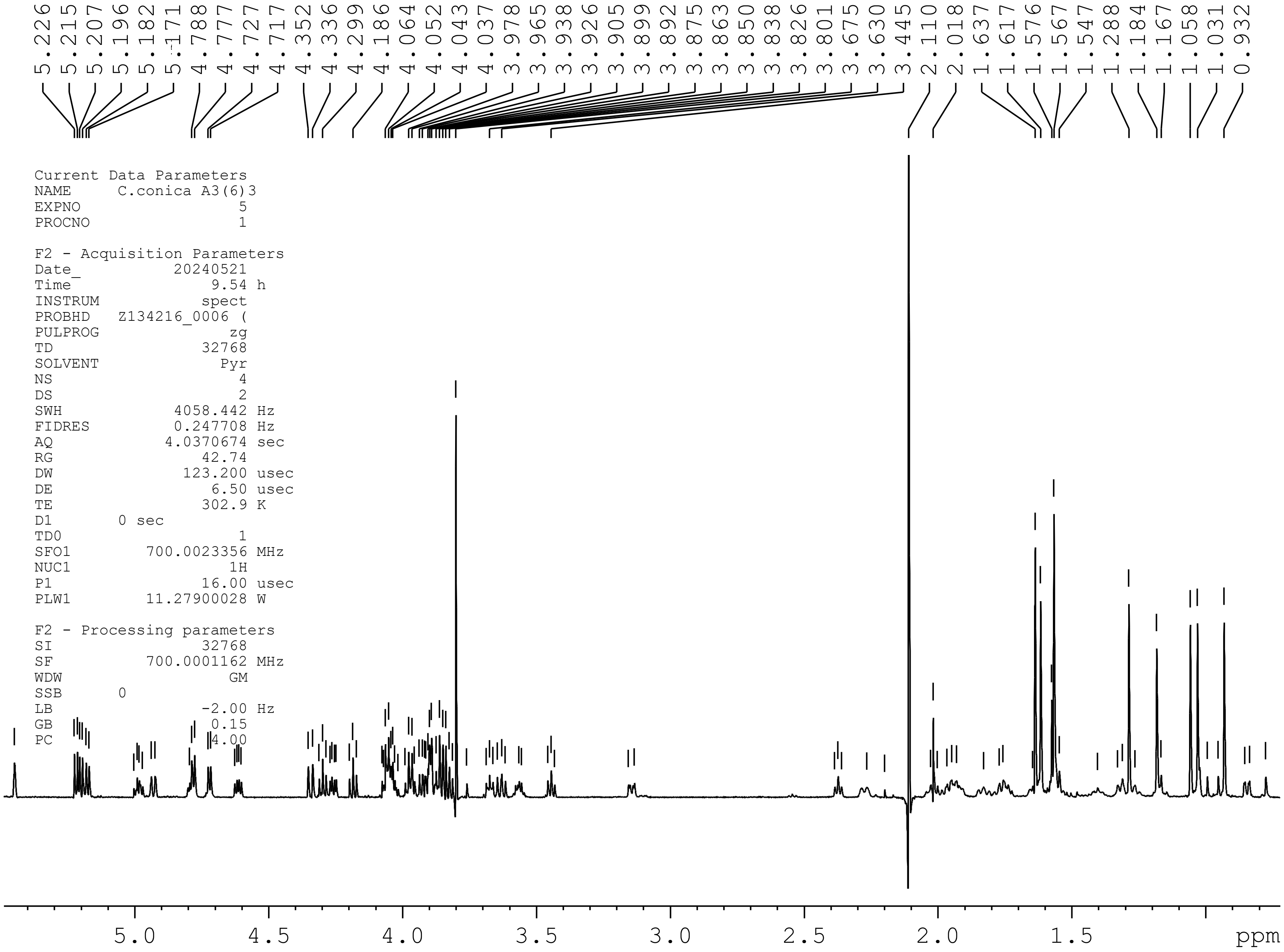

Figure S30. The ^{1}H NMR (700.13 MHz) spectrum of conicospermiumoside A3-3 (**3**) in C_5D_5N/D_2O (4/1)

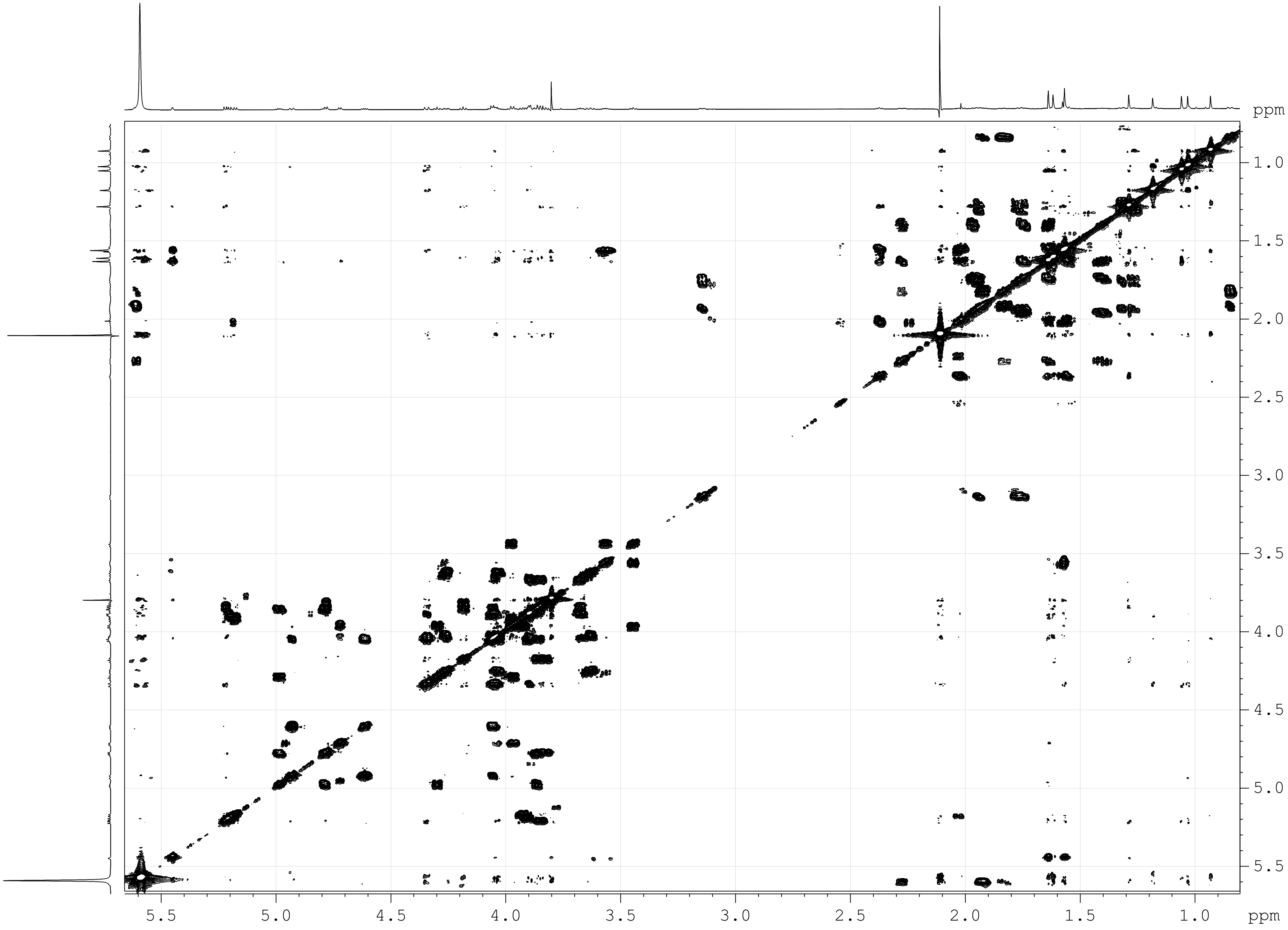

Figure S31. The COSY (700.13 MHz) spectrum of conicospermiumoside A3-3 (**3**) in C_5D_5N/D_2O (4/1)

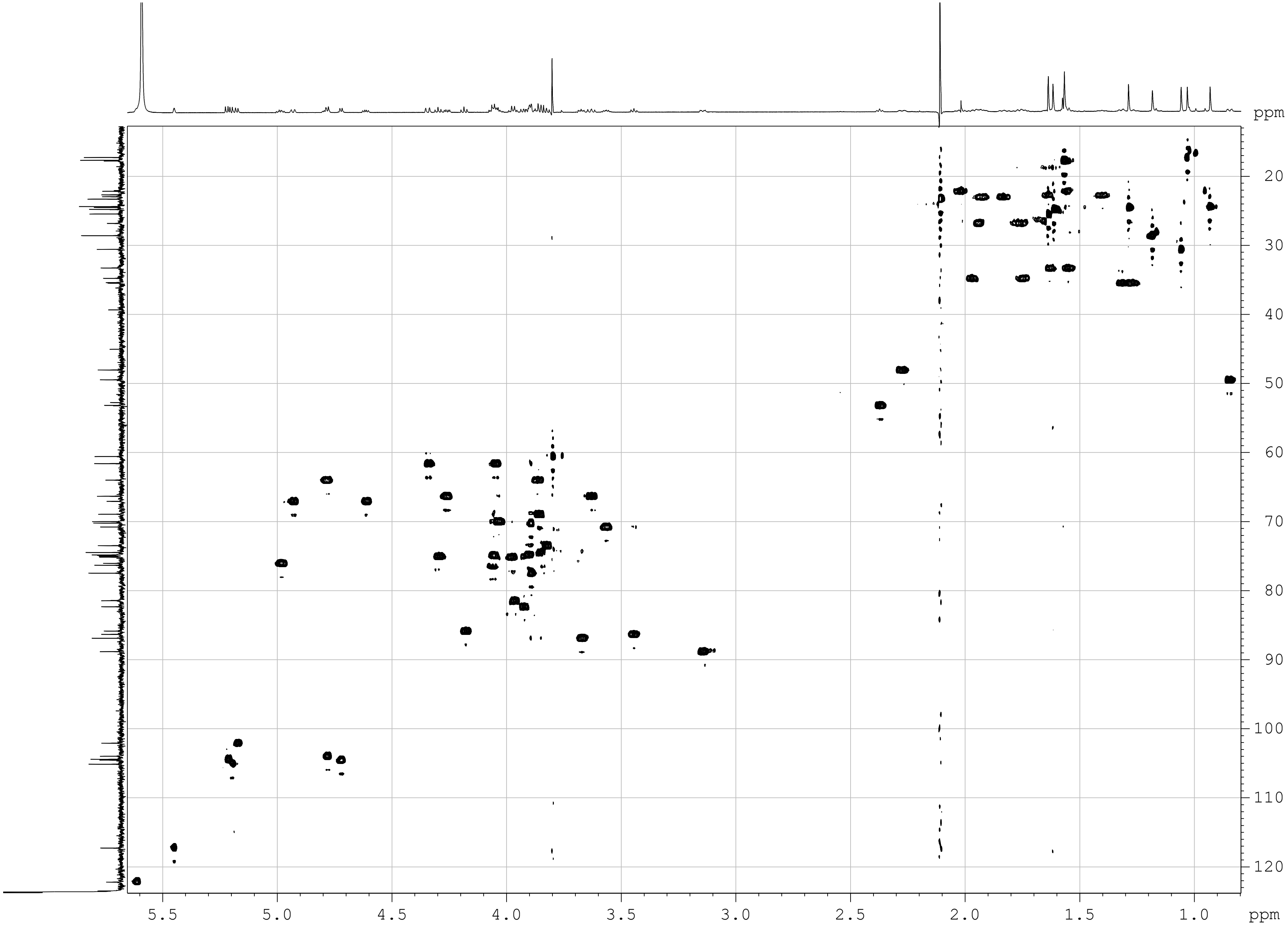

Figure S32. The HSQC (700.13 MHz) spectrum of conicospermiumoside A3-3 (**3**) in C_5D_5N/D_2O (4/1)

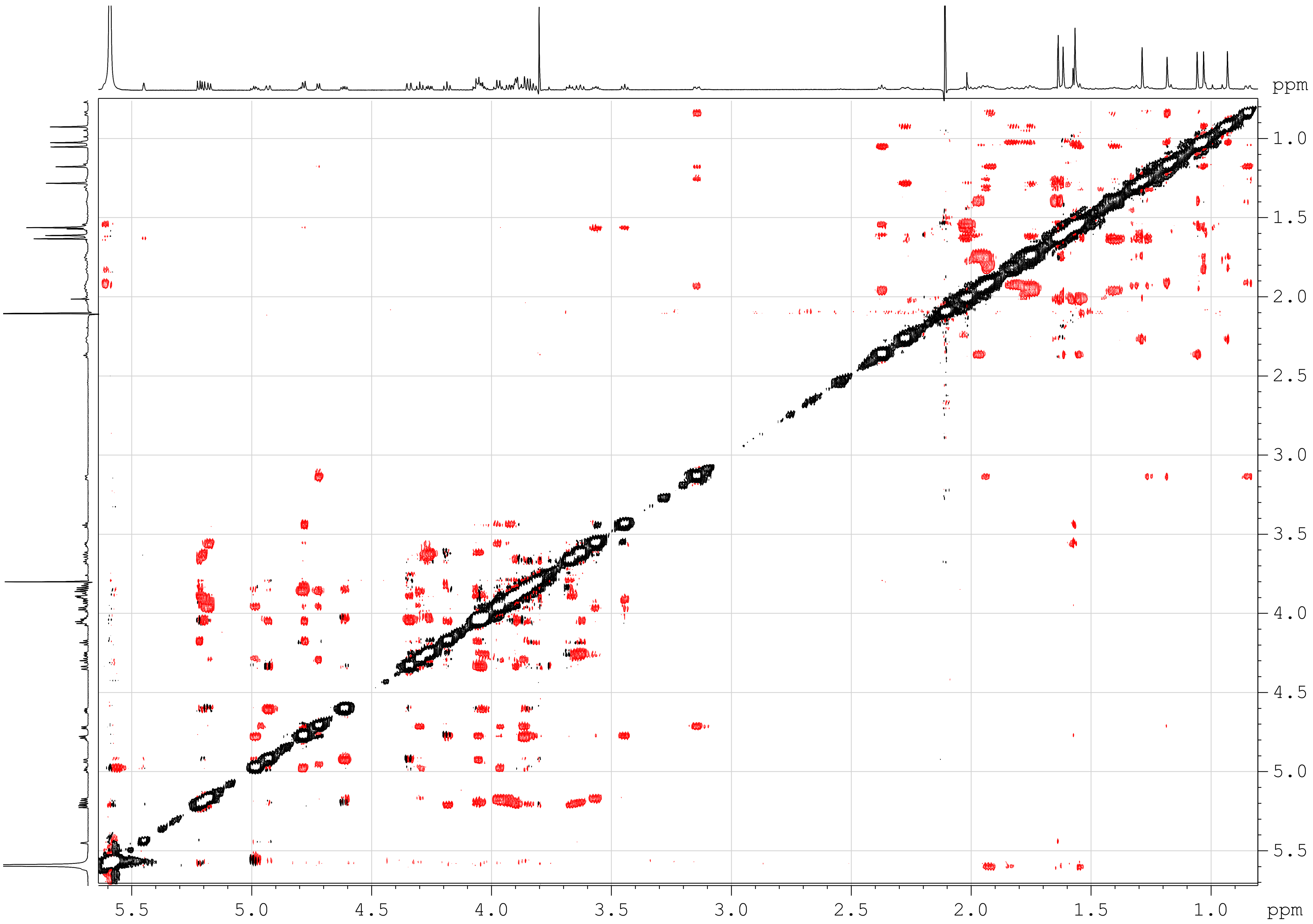

Figure S33. The ROESY (700.13 MHz) spectrum of conicospermiumoside A3-3 (**3**) in C_5D_5N/D_2O (4/1)

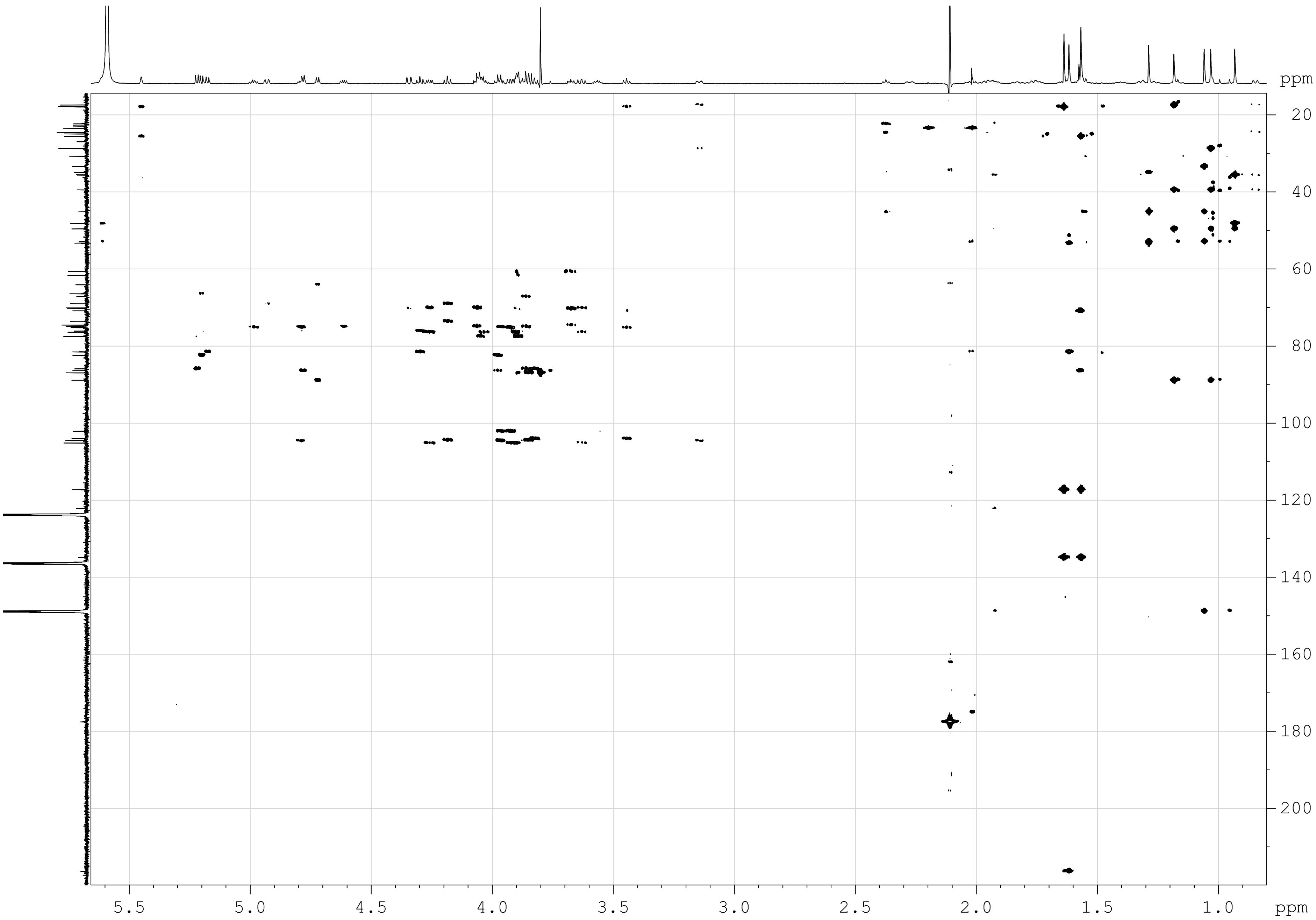

Figure S34. The HMBC (700.13 MHz) spectrum of conicospermiumoside A$_3$-3 (**3**) in C_5D_5N/D_2O (4/1)

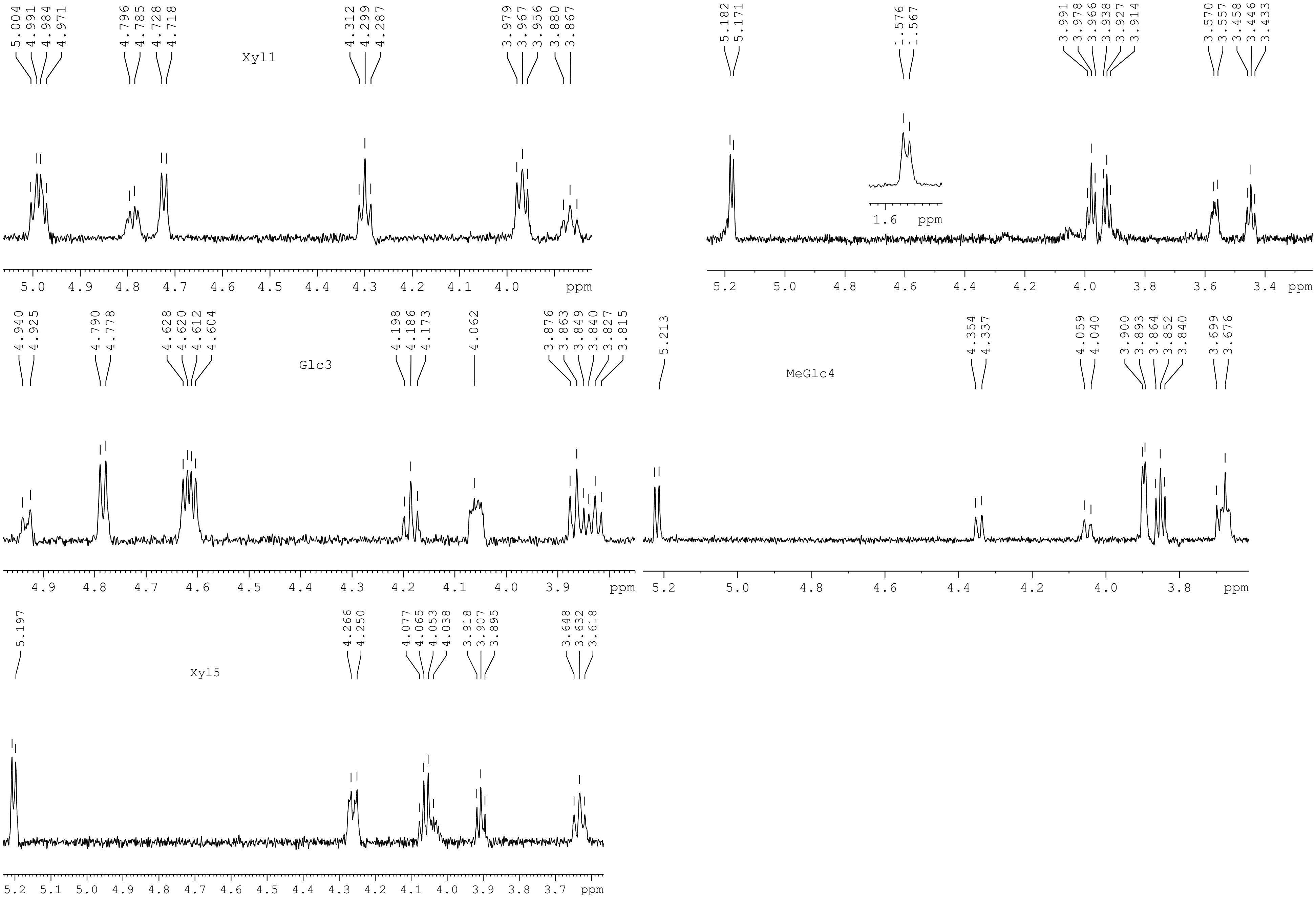

Figure S35. 1 D TOCSY (700.13 MHz) spectra of Xyl1, Qui2, Glc3, MeGlc4, Xyl5 of conicospermiumoside A3-3 (**3**) in C_5D_5N/D_2O (4/1)

Table S2. ^{13}C and ^{1}H NMR chemical shifts and HMBC and ROESY correlations of carbohydrate moiety of conicospermiumoside A3-3 (**3**)

Atom	δ_C mult.[a]	δ_H mult. (*J* in	HMBC	ROESY
Xyl1 (1→C-3)				
1	104.6 CH	4.72 d (6.8)	C: 3; C: 5 Xyl1	H-3; H-3, 5 Xyl1
2	**81.5** CH	3.97 t (7.8)	C: 1 Qui2; C: 1, 3 Xyl1	H-1 Qui2; H-4 Xyl1
3	75.1 CH	4.30 t (7.8)	C: 2, 4 Xyl1	H-1, 5 Xyl1
4	*76.1* CH	5.00 m	C: 3 Xyl1	H-2 Xyl1
5	64.1 CH_2	4.79 dd (7.8; 12.0)	C: 1, 3 Xyl1	
		3.87 t (9.7)		H-1, 3 Xyl1
Qui2 (1→2Xyl1)				
1	102.1 CH	5.18 d (7.5)	C: 2 Xyl1	H-2 Xyl1; H-3, 5 Qui2
2	**82.4** CH	3.93 t (8.6)	C: 1, 3 Qui2; C: 1 Xyl5	H-4 Qui2; H-1 Xyl5
3	75.2 CH	3.98 t (8.6)	C: 2, 4 Qui2	
4	**86.4** CH	3.45 t (8.6)	C: 3 Qui2; C: 1 Glc3	H-1 Glc3; H-2 Qui2
5	70.9 CH	3.56 dd (5.7; 8.6)		H-1, 3 Qui2
6	17.9 CH_3	1.57 d (5.7)	C: 4, 5 Qui2	
Glc3 (1→4Qui2)				
1	104.0 CH	4.78 d (8.1)	C: 4 Qui2	H-4 Qui2; H-3, 5 Glc3
2	73.6 CH	3.83 t (8.7)	C: 1, 3 Glc3	
3	**85.9** CH	4.19 t (8.7)	C: 2, 4 Glc3, C: 1 MeGlc4	H-1 MeGlc4; H-1, 5 Glc3
4	69.0 CH	3.86 t (8.7)	C: 3, 5, 6 Glc3	H-6 Glc3
5	75.0 CH	4.06 m	C: 1, 3 Xyl3	H-1, 3 Glc3
6	67.2 CH_2	4.93 brd (10.4)		
		4.62 dd (6.2; 11.2)	C: 5 Glc3	H-4 Glc3
MeGlc4 (1→3Glc3)				
1	104.5 CH	5.22 d (8.2)	C: 3 Glc3	H-3 Glc3; H-3, 5 MeGlc4
2	74.6 CH	3.85 t (8.2)	C: 1, 3 MeGlc4	
3	87.0 CH	3.68 t (8.2)	C: 2, 4 MeGlc4; OMe	H-1, 5 MeGlc4
4	70.3 CH	3.90 m	C: 3, 5 MeGlc4	
5	77.5 CH	3.90 m		H-1 MeGlc4
6	61.7 CH_2	4.35 d (11.8)		
		4.05 brd (11.8)	C: 5 MeGlc4	
OMe	60.7 CH_3	3.80 s	C: 3 MeGlc4	
Xyl5 (1→2Qui2)				
1	105.2 CH	5.20 d (7.8)	C: 2 Qui2	H-2 Qui2; H-3, 5 Xyl5
2	74.9 CH	3.91 t (8.6)	C: 1, 3 Xyl5	
3	76.4 CH	4.06 t (8.6)	C: 2, 4 Xyl5	
4	70.1 CH	4.04 m	C: 3 Xyl5	
5	66.4 CH_2	4.26 dd (4.7; 11.7)	C: 1, 3, 4 Xyl5	
		3.63 brt (10.9)	C: 1, 3, 4 Xyl5	H-1 Xyl5

[a] Recorded at 176.04 MHz in C_5D_5N/D_2O. [b] Bold—interglycosidic positions. [c] Italics—sulfate position. [d] Recorded at 700.13 MHz in C_5D_5N/D_2O. Multiplicity by 1D TOCSY.

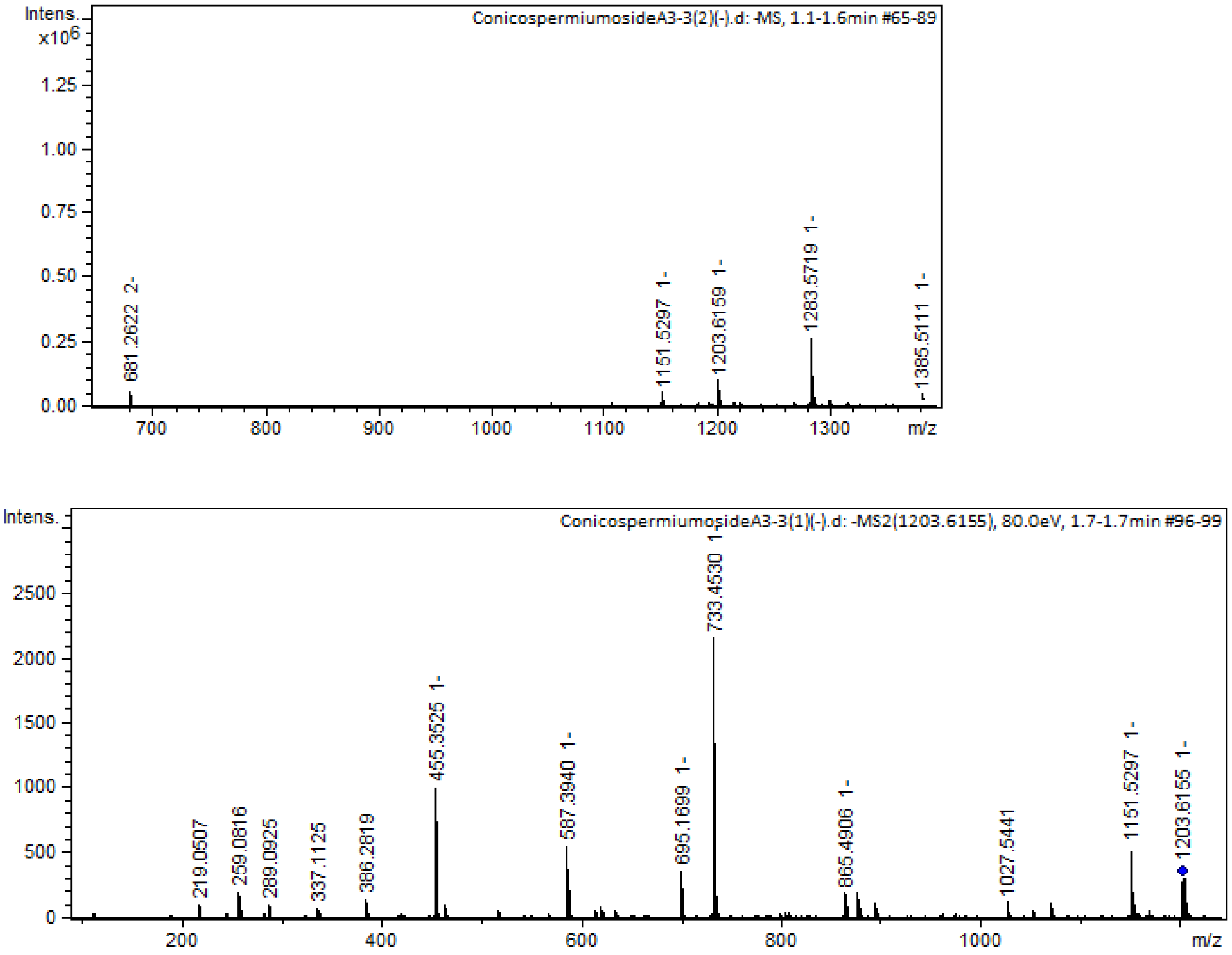

Figure S36. HR-ESI-MS and ESI-MS/MS spectra of conicospermiumoside A3-3 (**3**)

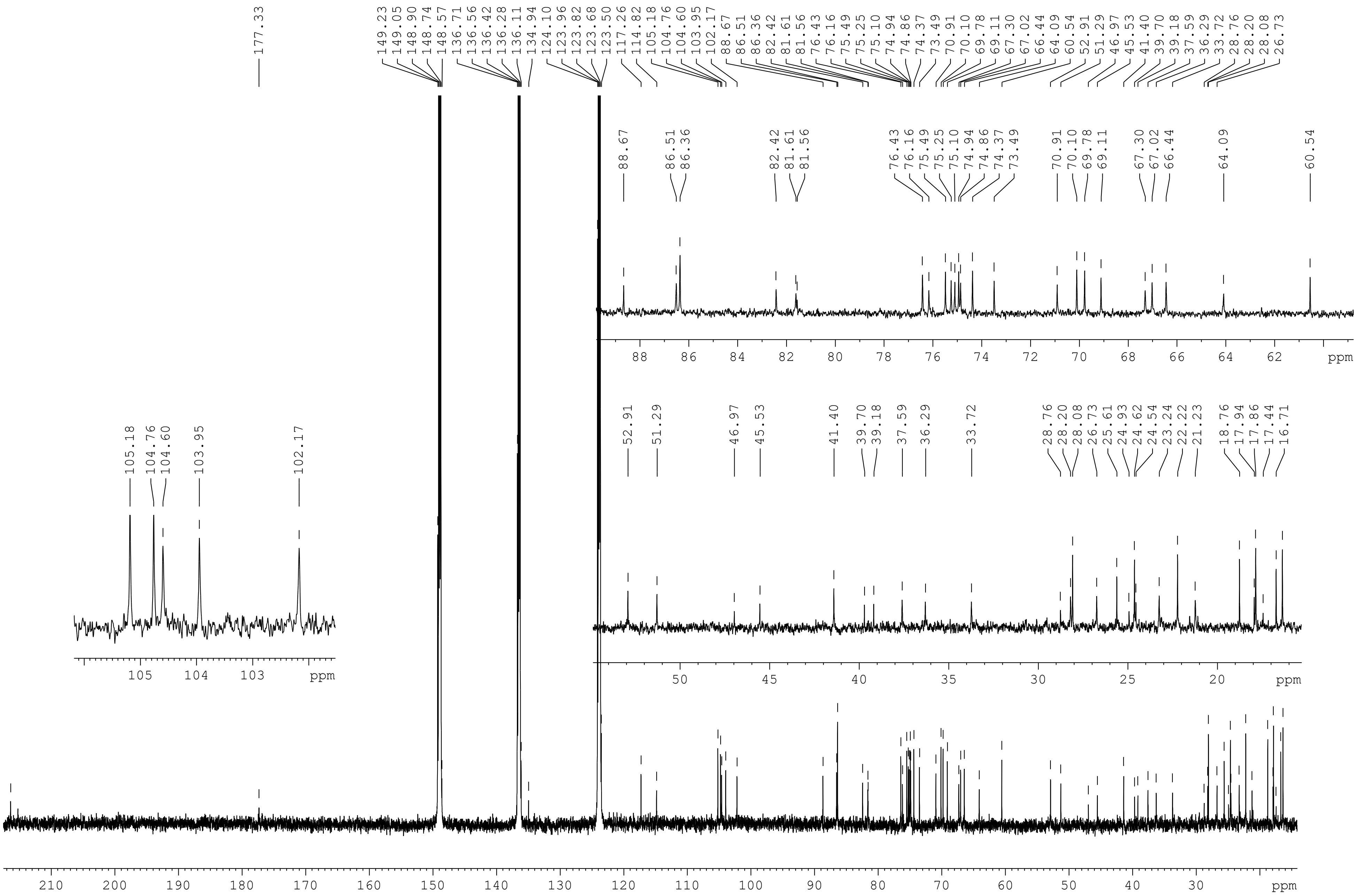

Figure S37. The ¹³C NMR (125.67 MHz) spectrum of conicospermiumoside A_7-1 (**4**) in C_5D_5N/D_2O (4/1)

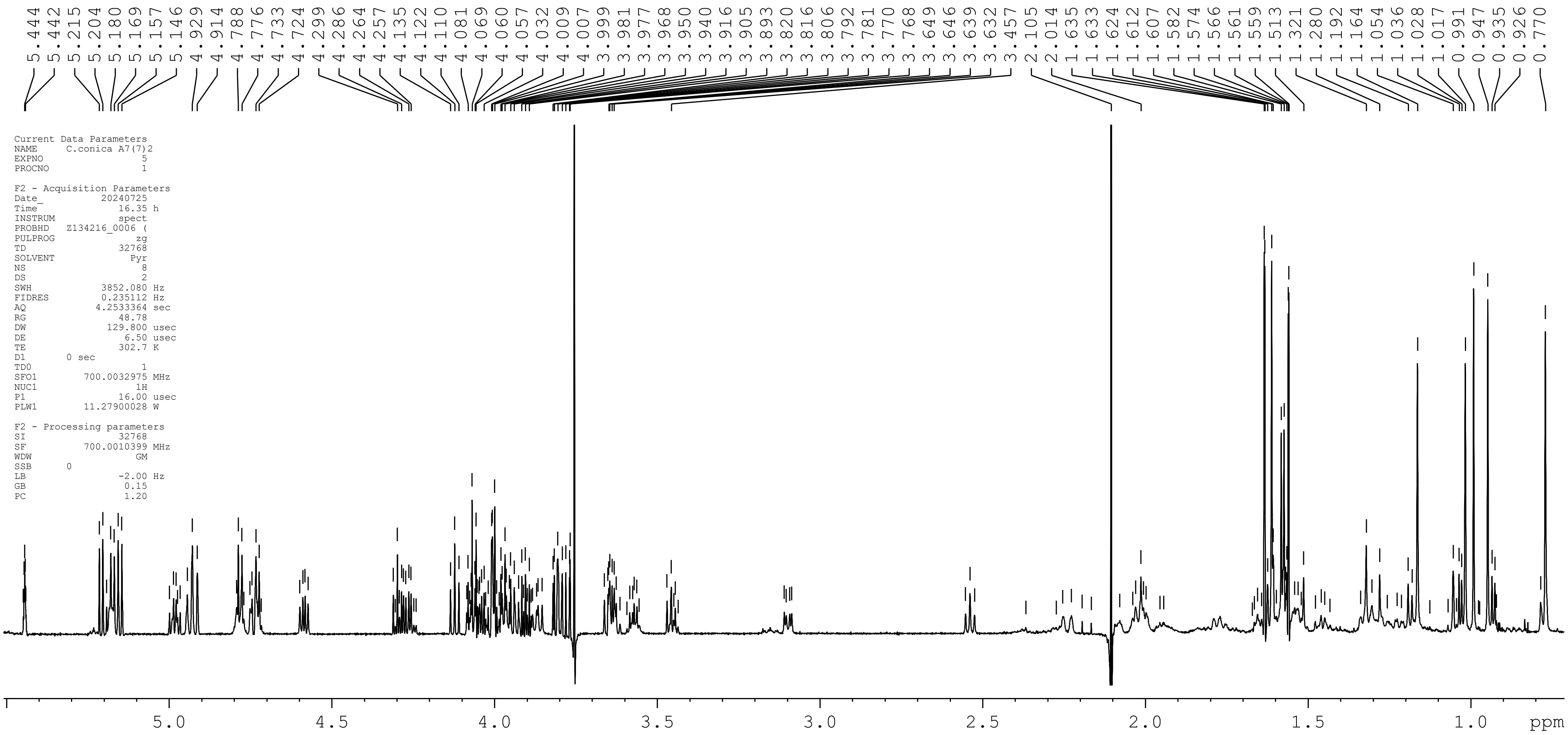

Figure S38. The ^{1}H NMR (700.13 MHz) spectrum of conicospermiumoside A$_7$-1 (**4**) in C_5D_5N/D_2O (4/1)

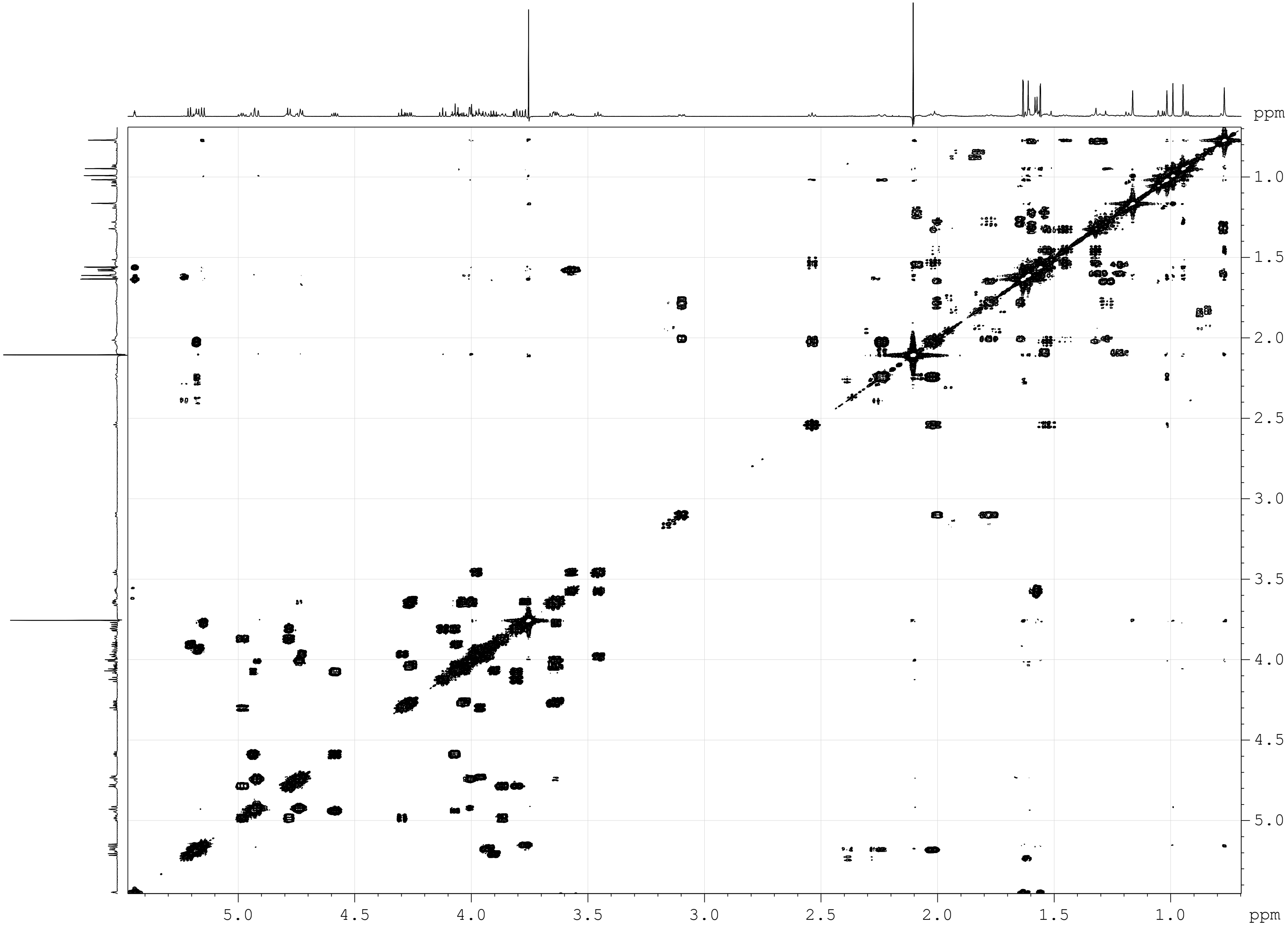

Figure S39. The COSY (700.13 MHz) spectrum of conicospermiumoside A7-1 (**4**) in C_5D_5N/D_2O (4/1)

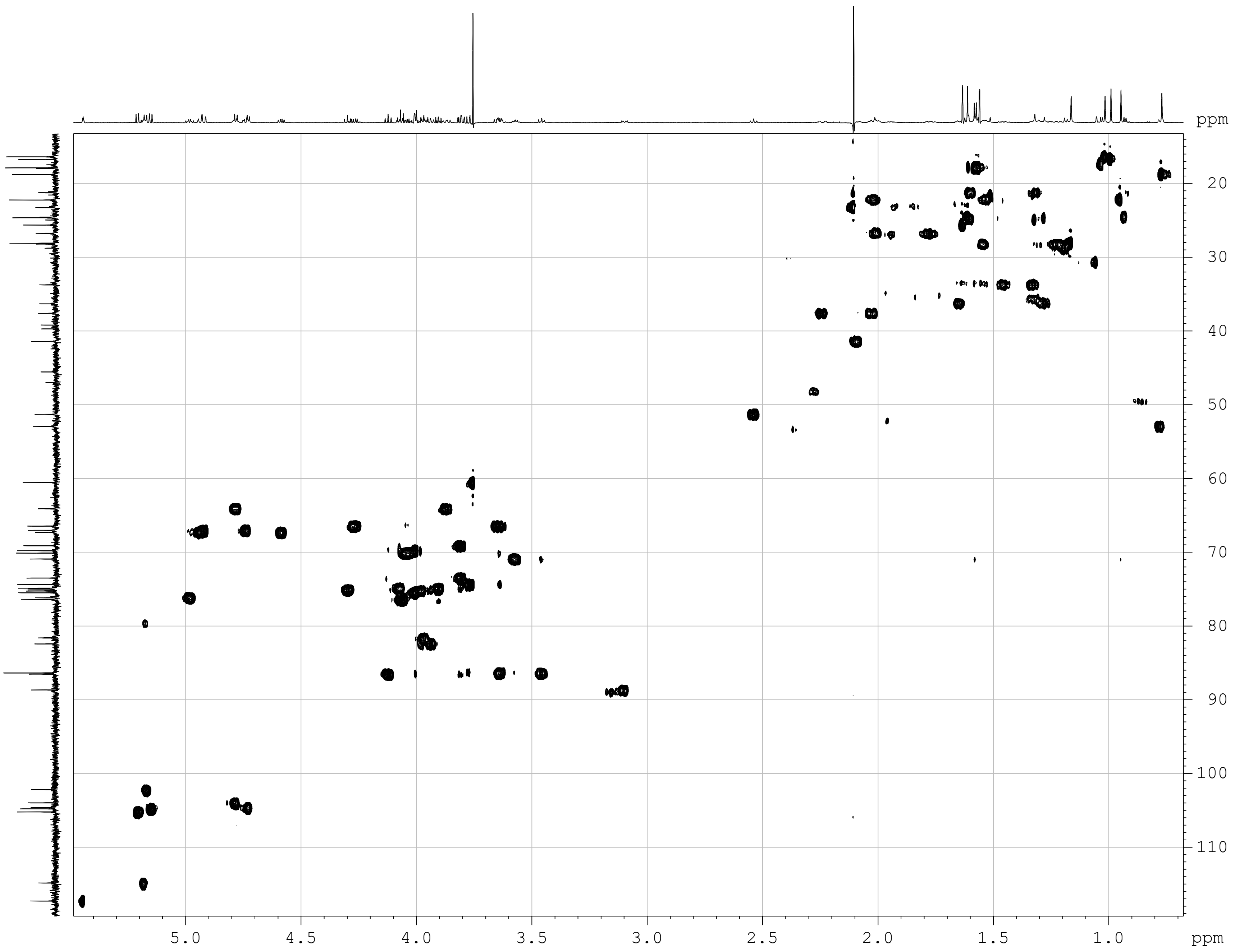

Figure S40. The HSQC (700.13 MHz) spectrum of conicospermiumoside A7-1 (**4**) in C_5D_5N/D_2O (4/1)

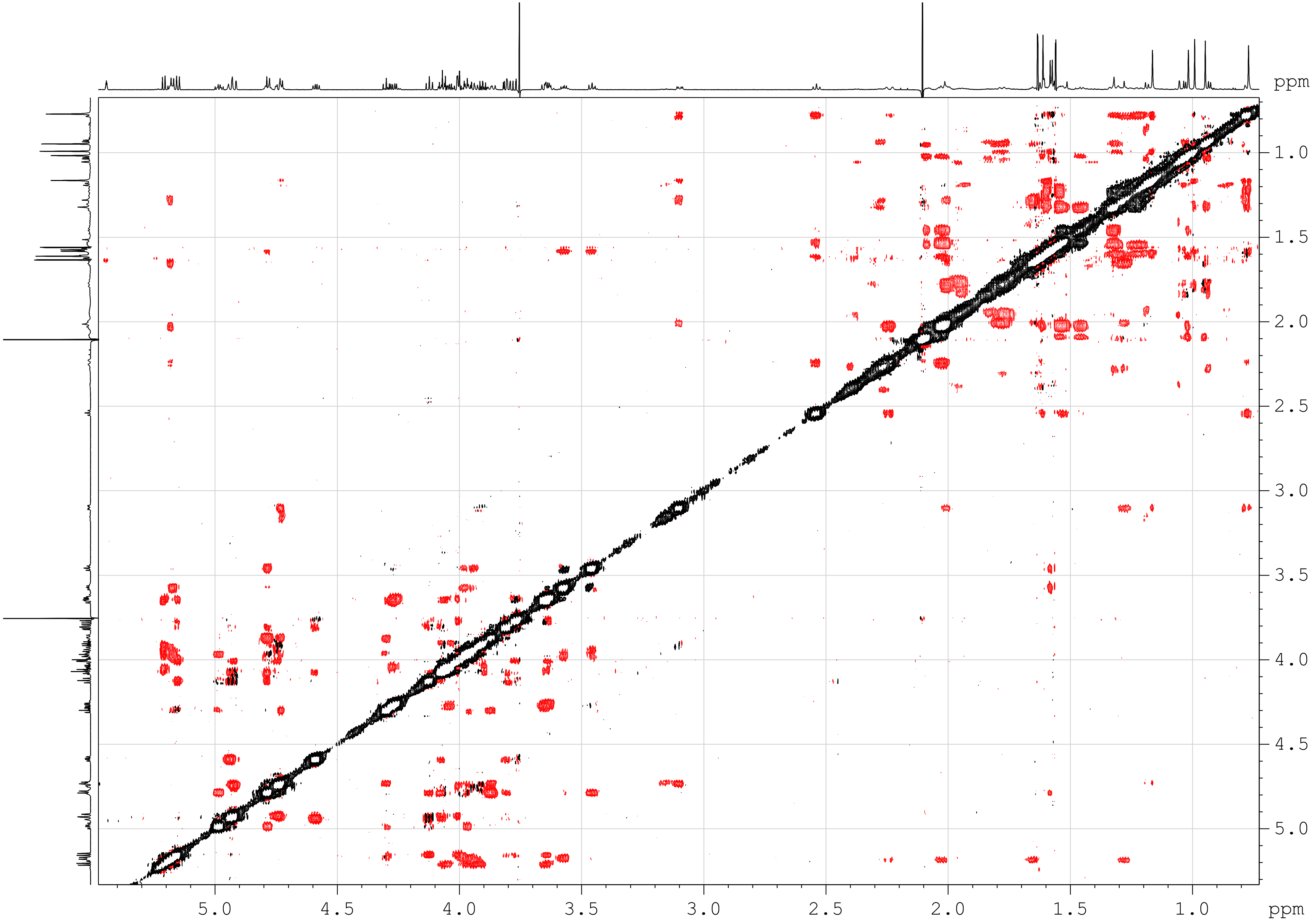

Figure S41. The ROESY (700.13 MHz) spectrum of conicospermiumoside A7-1 (**4**) in C_5D_5N/D_2O (4/1)

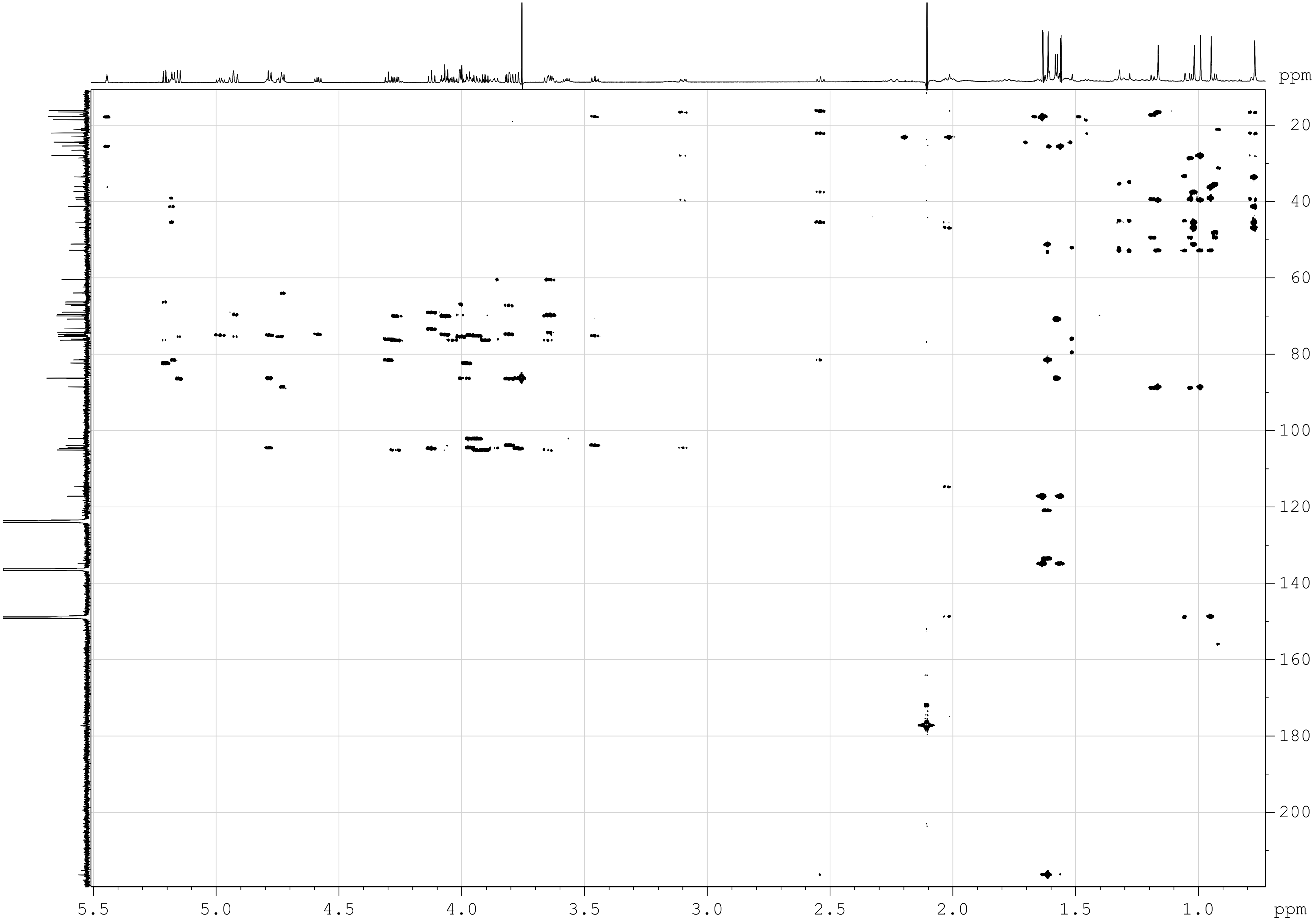

Figure S42. The HMBC (700.13 MHz) spectrum of conicospermiumoside A7-1 (**4**) in C_5D_5N/D_2O (4/1)

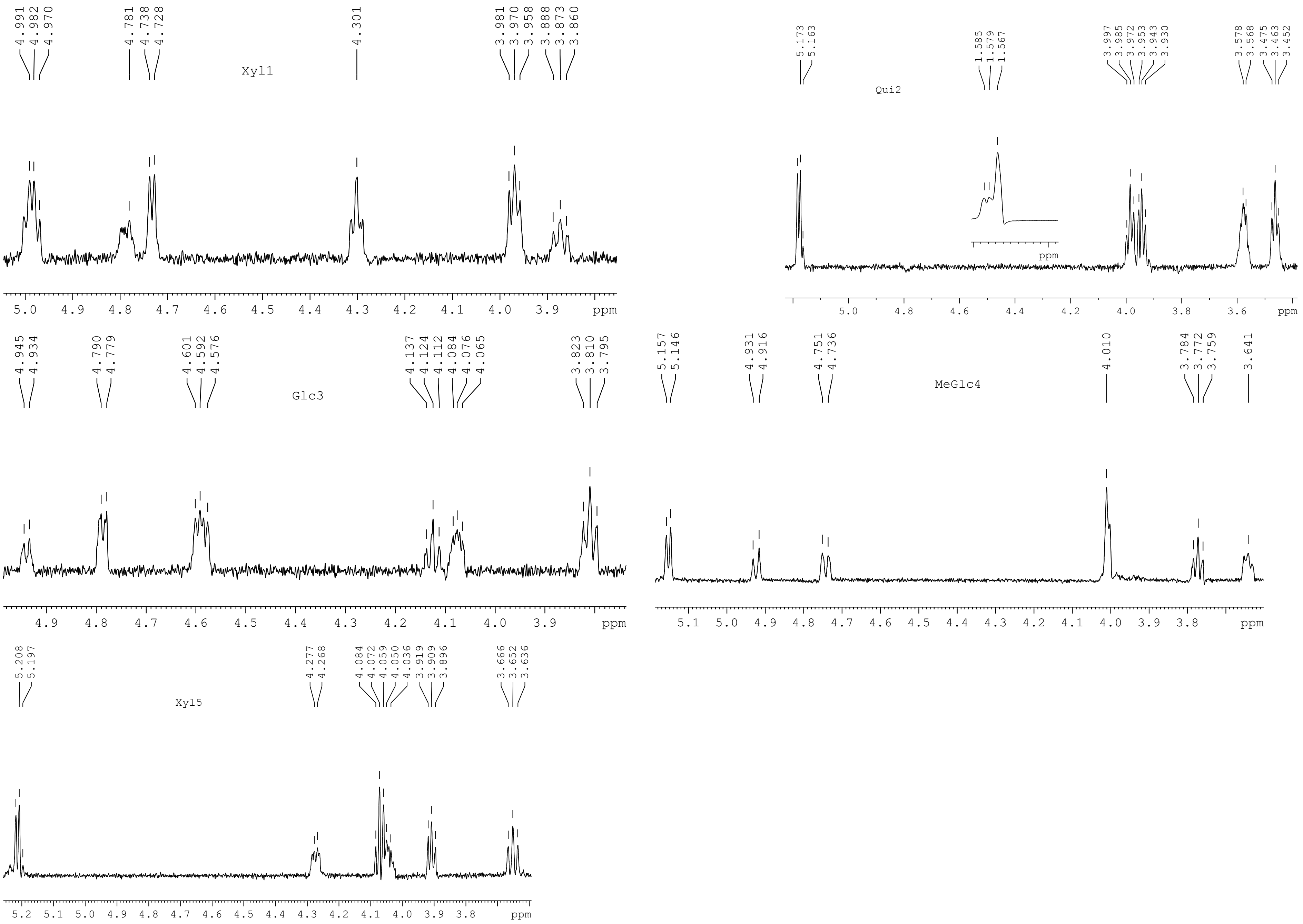

Figure S43. 1 D TOCSY (700.13 MHz) spectra of Xyl1, Qui2, Glc3, MeGlc4, Xyl5 of conicospermiumoside A$_7$-1 (**4**) in C_5D_5N/D_2O (4/1)

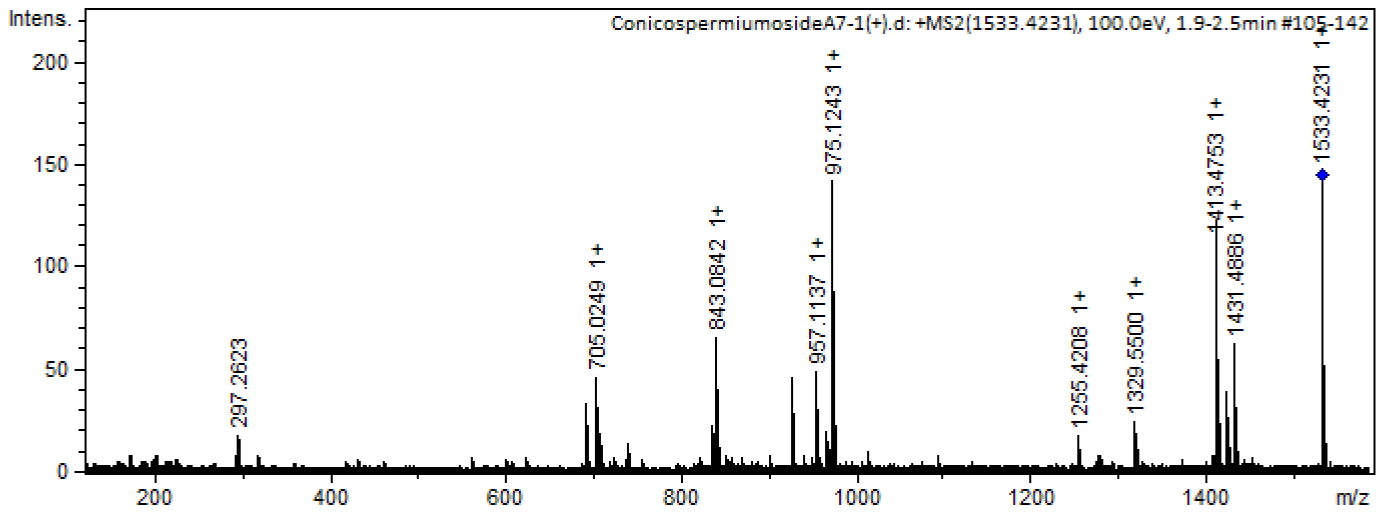

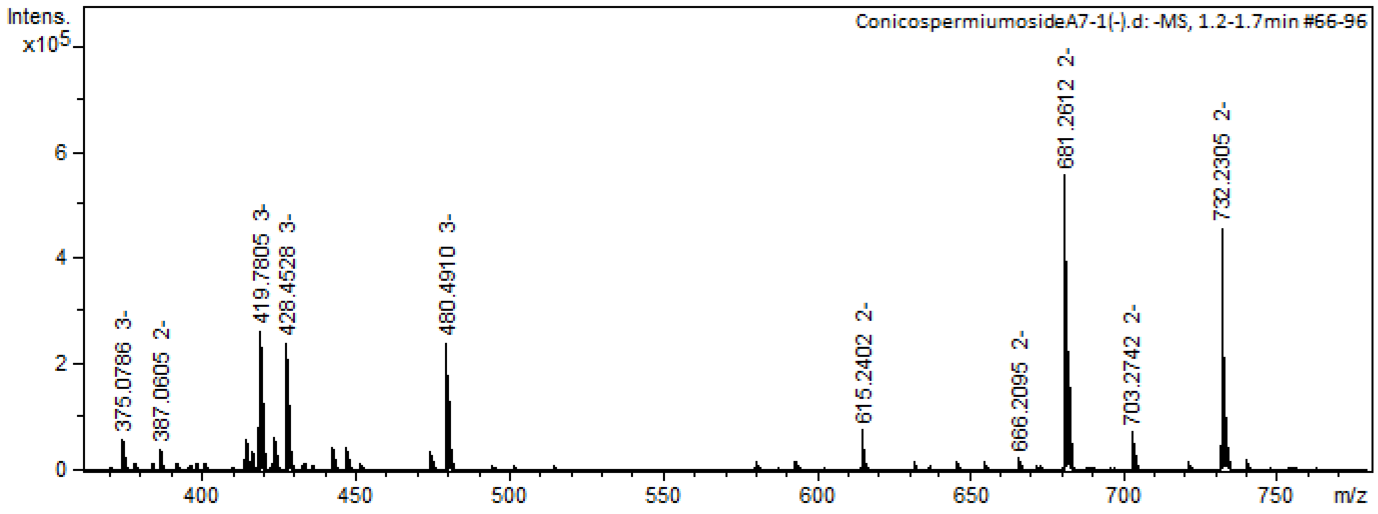

Figure S44. HR-ESI-MS and ESI-MS/MS spectra of conicospermiumoside A$_7$-1 (**4**)

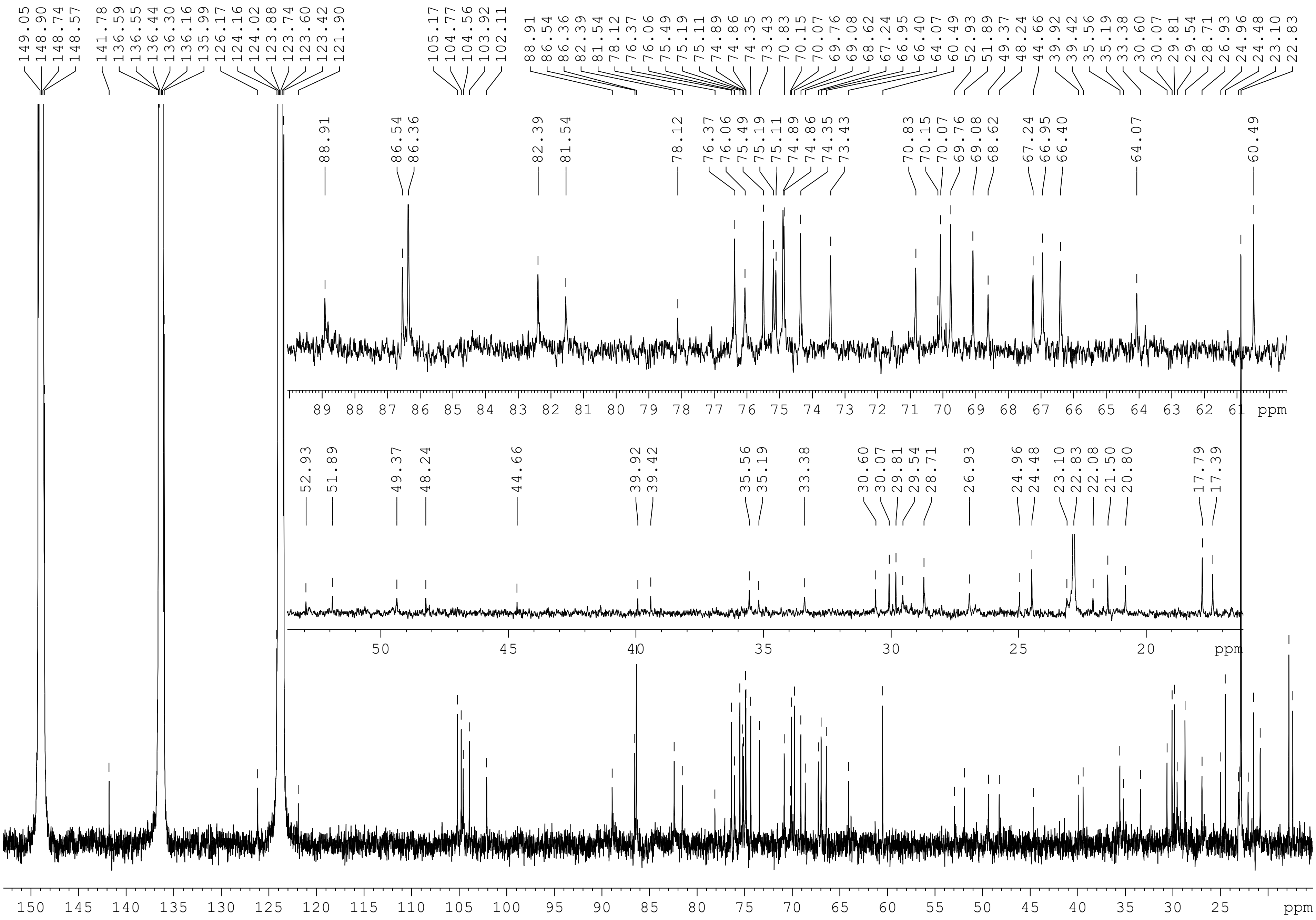

Figure S45. The ^{13}C NMR (176.04 MHz) spectrum of conicospermiumoside A_7-2 (**5**) in C_5D_5N/D_2O (4/1)

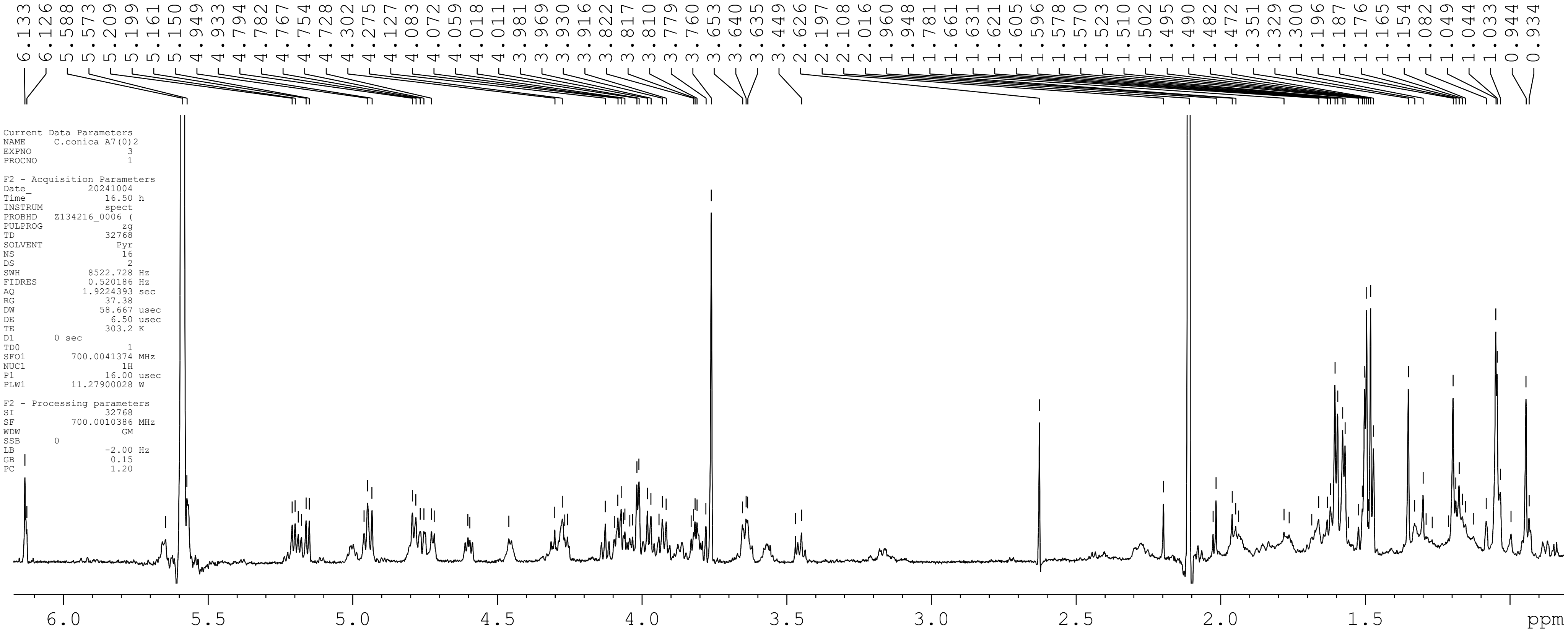

Figure S46. The ^1H NMR (700.13 MHz) spectrum of conicospermiumoside A7-2 (**5**) in C_5D_5N/D_2O (4/1)

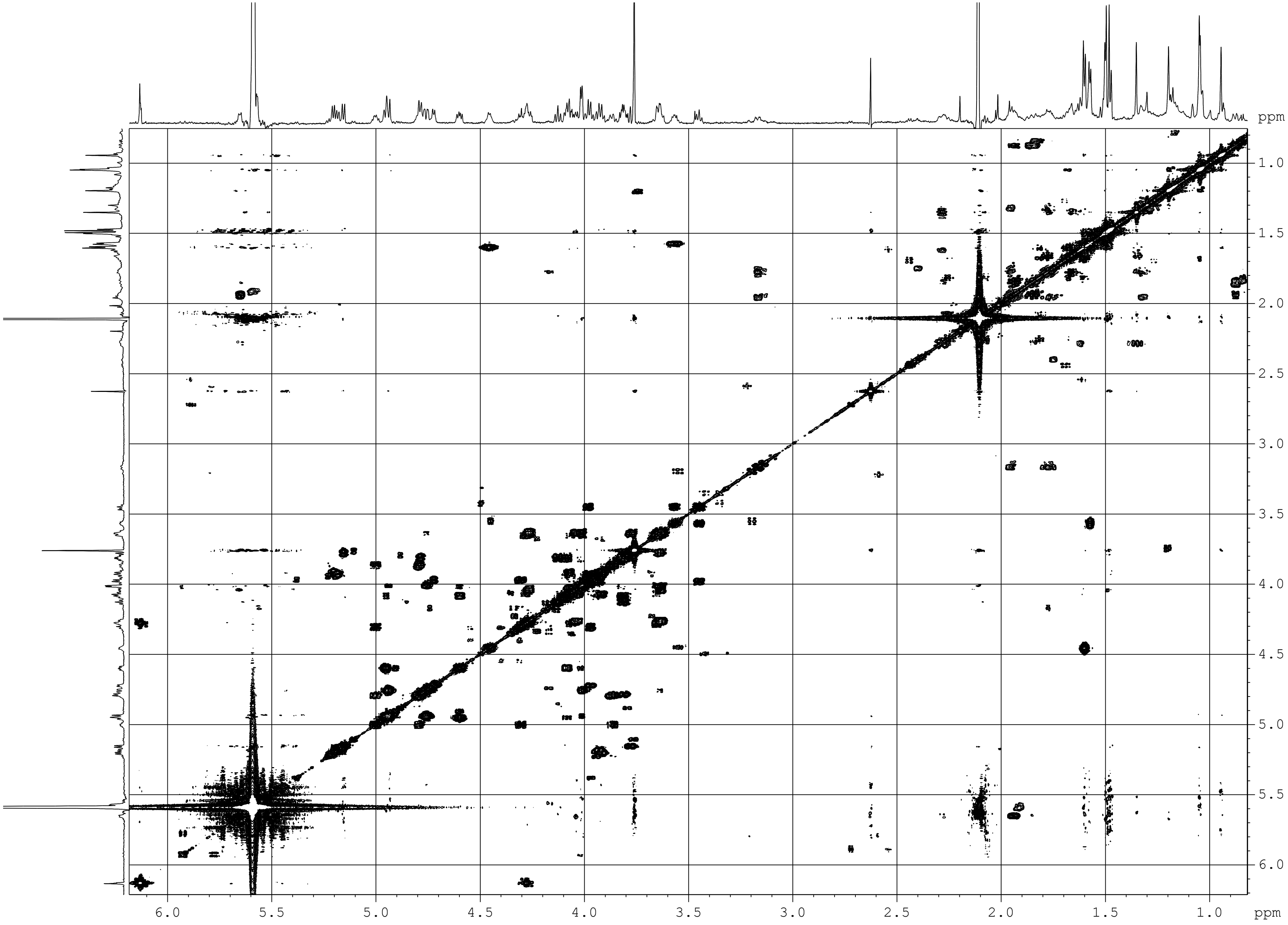

Figure S47. The COSY (700.13 MHz) spectrum of conicospermiumoside A_7-2 (**5**) in C_5D_5N/D_2O (4/1)

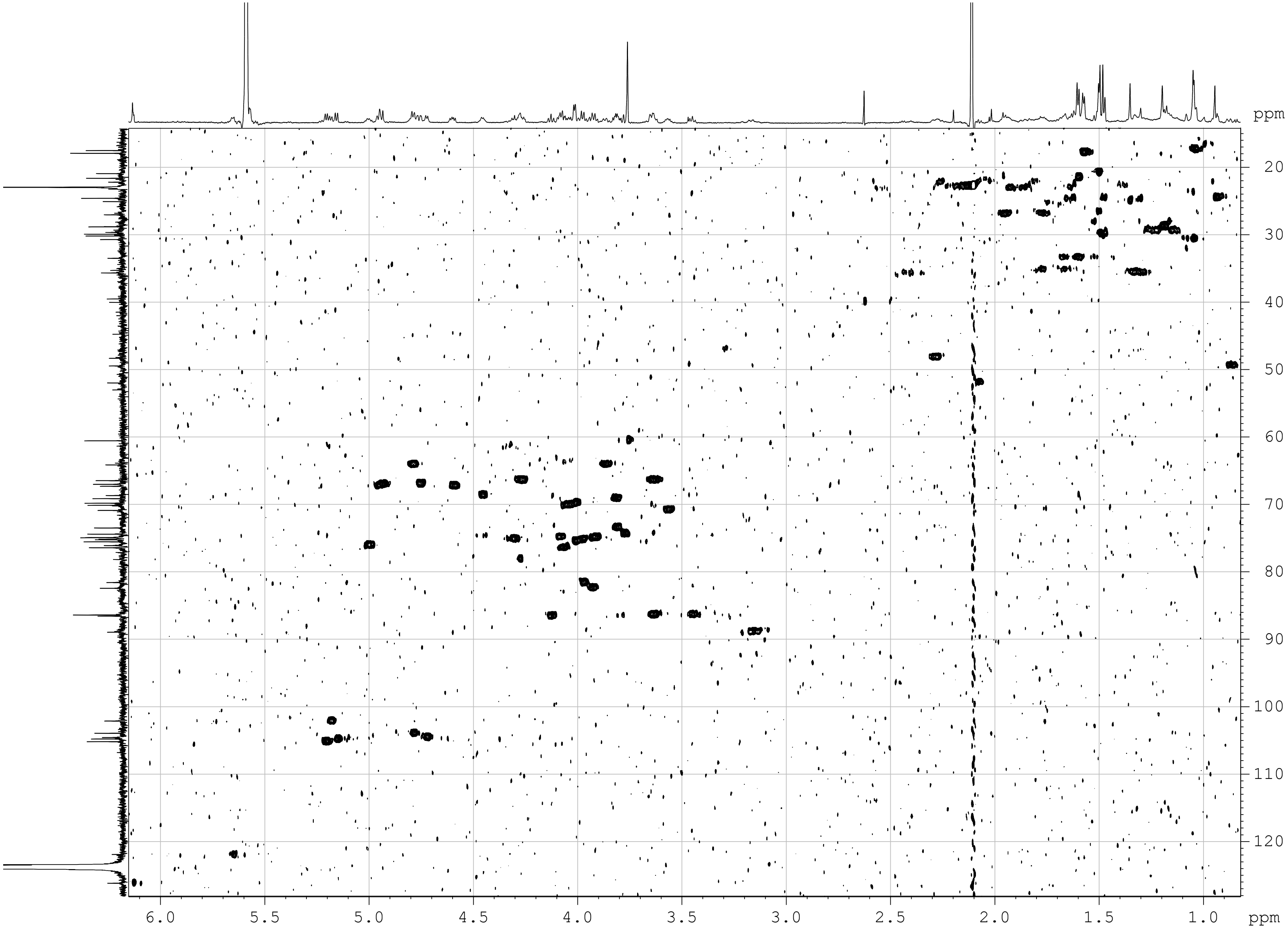

Figure S48. The HSQC (700.13 MHz) spectrum of conicospermiumoside A7-2 (**5**) in C_5D_5N/D_2O (4/1)

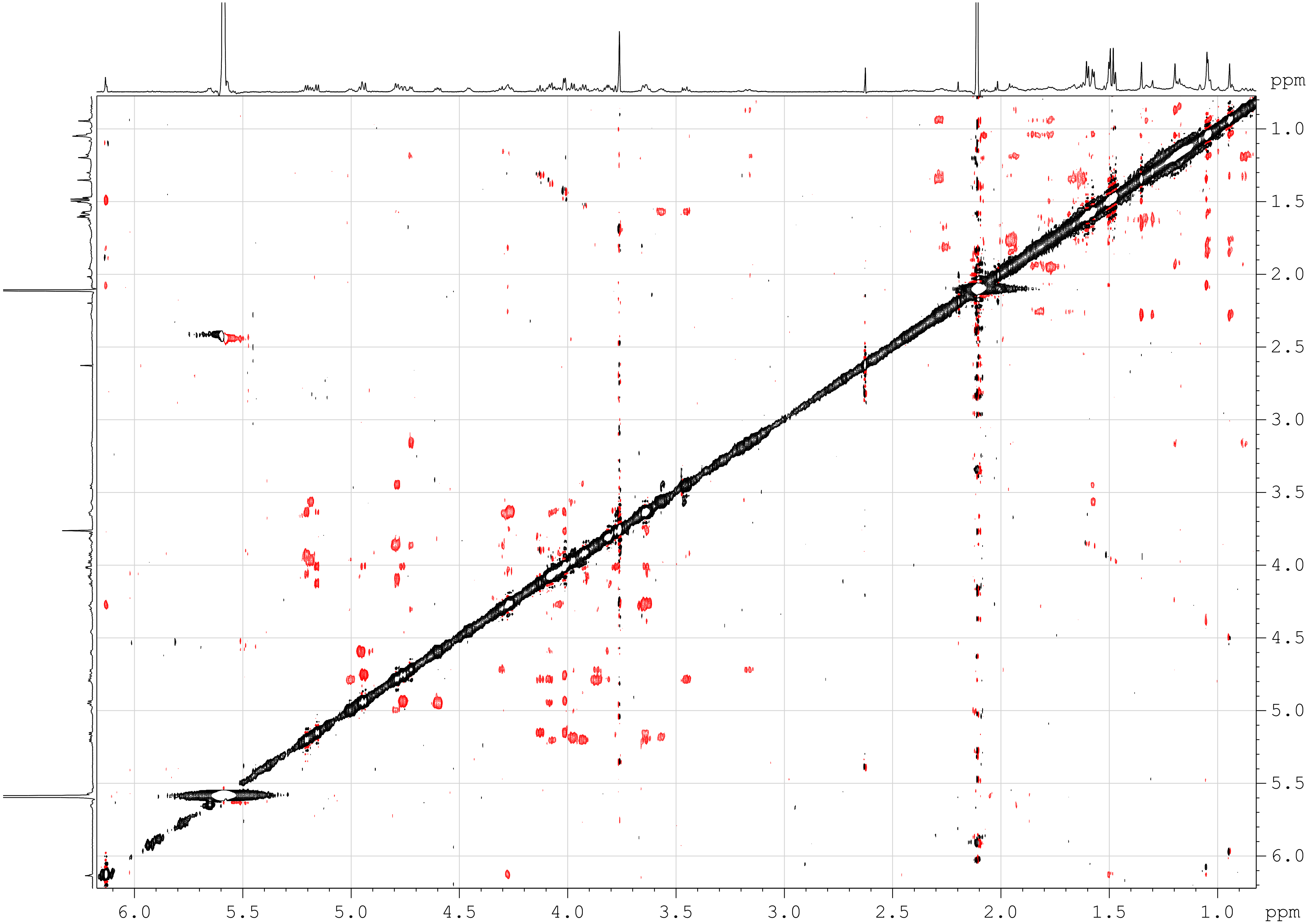

Figure S49. The ROESY (700.13 MHz) spectrum of conicospermiumoside A_7-2 (**5**) in C_5D_5N/D_2O (4/1)

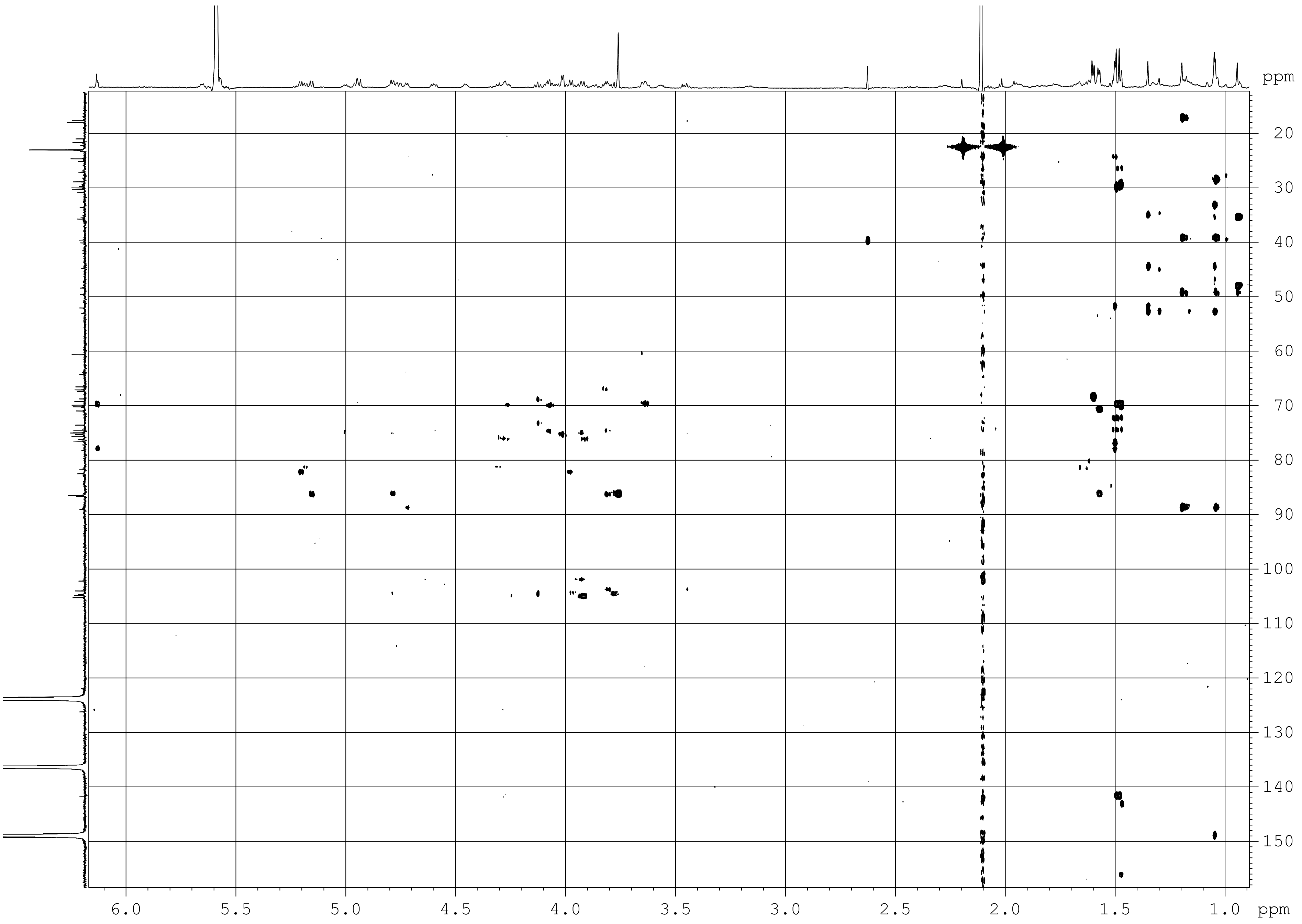

Figure S50. The HMBC (700.13 MHz) spectrum of conicospermiumoside A_7-2 (**5**) in C_5D_5N/D_2O (4/1)

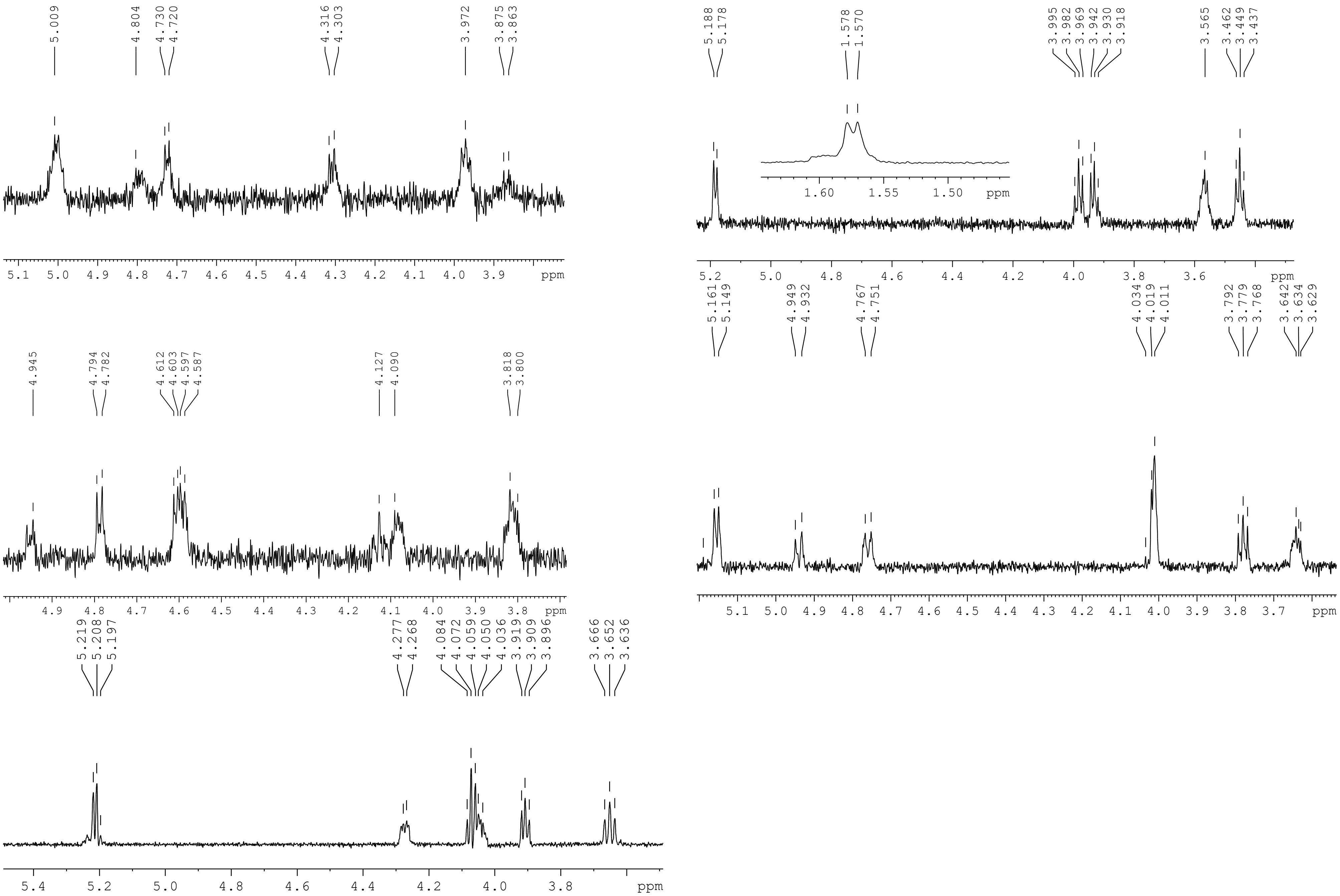

Figure S51. 1 D TOCSY (700.13 MHz) spectra of Xyl1, Qui2, Glc3, MeGlc4, Xyl5 of conicospermiumoside A7-2 (**5**) in C_5D_5N/D_2O (4/1)

Table S3. ^{13}C and ^{1}H NMR chemical shifts and HMBC and ROESY correlations of carbohydrate moiety of conicospermiumoside A_7-2 (**5**)

Atom	δ_C mult.[a]	δ_H mult. (*J* in	HMBC	ROESY
Xyl1 (1→C-3)				
1	104.6 CH	4.72 d (6.7)	C: 3; C: 5 Xyl1	H-3; H-3, 5 Xyl1
2	**81.5** CH	3.97 t (8.7)	C: 1 Qui2; C: 1, 3 Xyl1	H-1 Qui2; H-4 Xyl1
3	75.1 CH	4.30 t (8.7)	C: 2, 4 Xyl1	H-1, 5 Xyl1
4	*76.1* CH	5.01 m	C: 3 Xyl1	H-2 Xyl1
5	64.1 CH_2	4.78 dd (4.7; 11.4)	C: 1, 4 Xyl1	
		3.87 dd (8.7; 11.4)		H-1, 3 Xyl1
Qui2 (1→2Xyl1)				
1	102.1 CH	5.18 d (7.3)	C: 2 Xyl1	H-2 Xyl1; H-3, 5 Qui2
2	**82.4** CH	3.93 t (9.4)	C: 1, 3 Qui2; C: 1 Xyl5	H-4 Qui2; H-1 Xyl5
3	75.2 CH	3.98 t (9.4)	C: 2, 4 Qui2	H-1, 5 Qui2
4	**86.4** CH	3.45 t (9.4)	C: 3 Qui2; C: 1 Glc3	H-1 Glc3; H-2 Qui2
5	70.9 CH	3.57 m		H-1, 3 Qui2
6	17.8 CH_3	1.57 d (5.8)	C: 4, 5 Qui2	
Glc3 (1→4Qui2)				
1	103.9 CH	4.79 d (7.7)	C: 4 Qui2	H-4 Qui2; H-3, 5 Glc3
2	73.4 CH	3.82 t (8.8)	C: 1, 3 Glc3	
3	**86.5** CH	4.13 t (8.8)	C: 4 Glc3, C: 1 MeGlc4	H-1 MeGlc4; H-1 Glc3
4	69.1 CH	3.82 t (8.8)	C: 5, 6 Glc3	H-6 Glc3
5	74.9 CH	4.09 m		H-1 Glc3
6	*67.2* CH_2	4.95 brd (10.4)		
		4.60 dd (6.7; 11.0)	C: 5 Glc3	H-4 Glc3
MeGlc4 (1→3Glc3)				
1	104.8 CH	5.16 d (8.6)	C: 3 Glc3	H-3 Glc3; H-3, 5 MeGlc4
2	74.3 CH	3.78 t (8.6)	C: 1 MeGlc4	H-4 MeGlc4
3	86.4 CH	3.63 t (8.6)	C: 2, 4 MeGlc4; OMe	H-1, 5 MeGlc4
4	69.8 CH	4.03 m	C: 5 MeGlc4	H-2, 6 MeGlc4
5	75.5 CH	4.02 m	C: 6 MeGlc4	H-1, 3 MeGlc4
6	*67.0* CH_2	4.94 d (11.9)	C: 4, 5 MeGlc4	
		4.76 brd (11.9)	C: 5 MeGlc4	
OMe	60.5 CH_3	3.76 s	C: 3 MeGlc4	
Xyl5 (1→2Qui2)				
1	105.2 CH	5.21 d (7.4)	C: 2 Qui2	H-2 Qui2; H-3, 5 Xyl5
2	74.9 CH	3.91 t (9.6)	C: 1, 3 Xyl5	H-4 Xyl5
3	76.4 CH	4.07 t (9.6)	C: 2, 4 Xyl5	H-1, 5 Xyl5
4	70.1 CH	4.05 m		
5	66.4 CH_2	4.27 dd (5.3; 11.7)	C: 1, 3, 4 Xyl5	H-3 Xyl5
		3.65 t (11.7)		H-1, 3 Xyl5

[a] Recorded at 176.04 MHz in C_5D_5N/D_2O. [b] Bold—interglycosidic positions. [c] Italics—sulfate position. [d] Recorded at 700.13 MHz in C_5D_5N/D_2O. Multiplicity by 1D TOCSY.

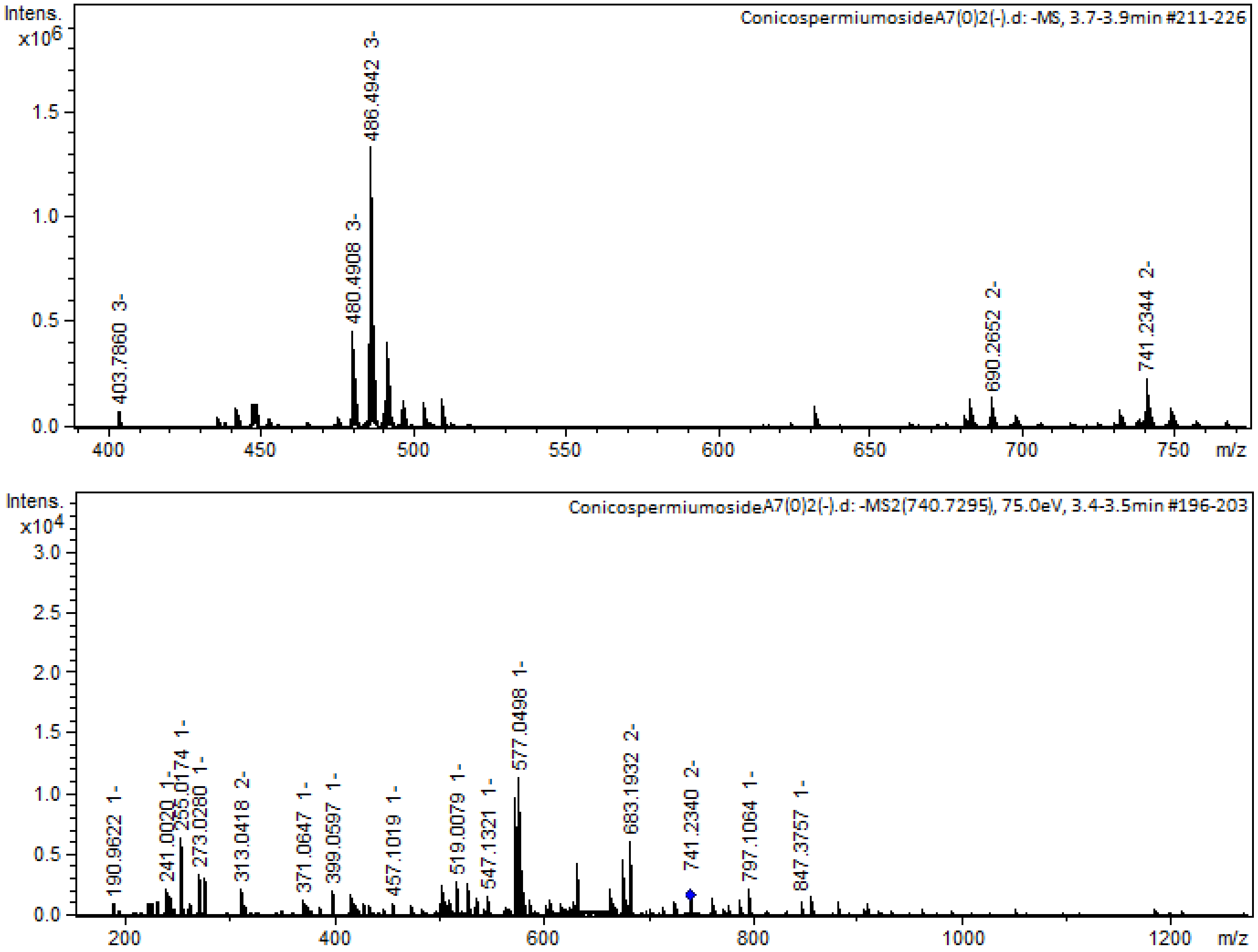

Figure S52. HR-ESI-MS and ESI-MS/MS spectra of conicospermiumoside A$_7$-2 (**5**)

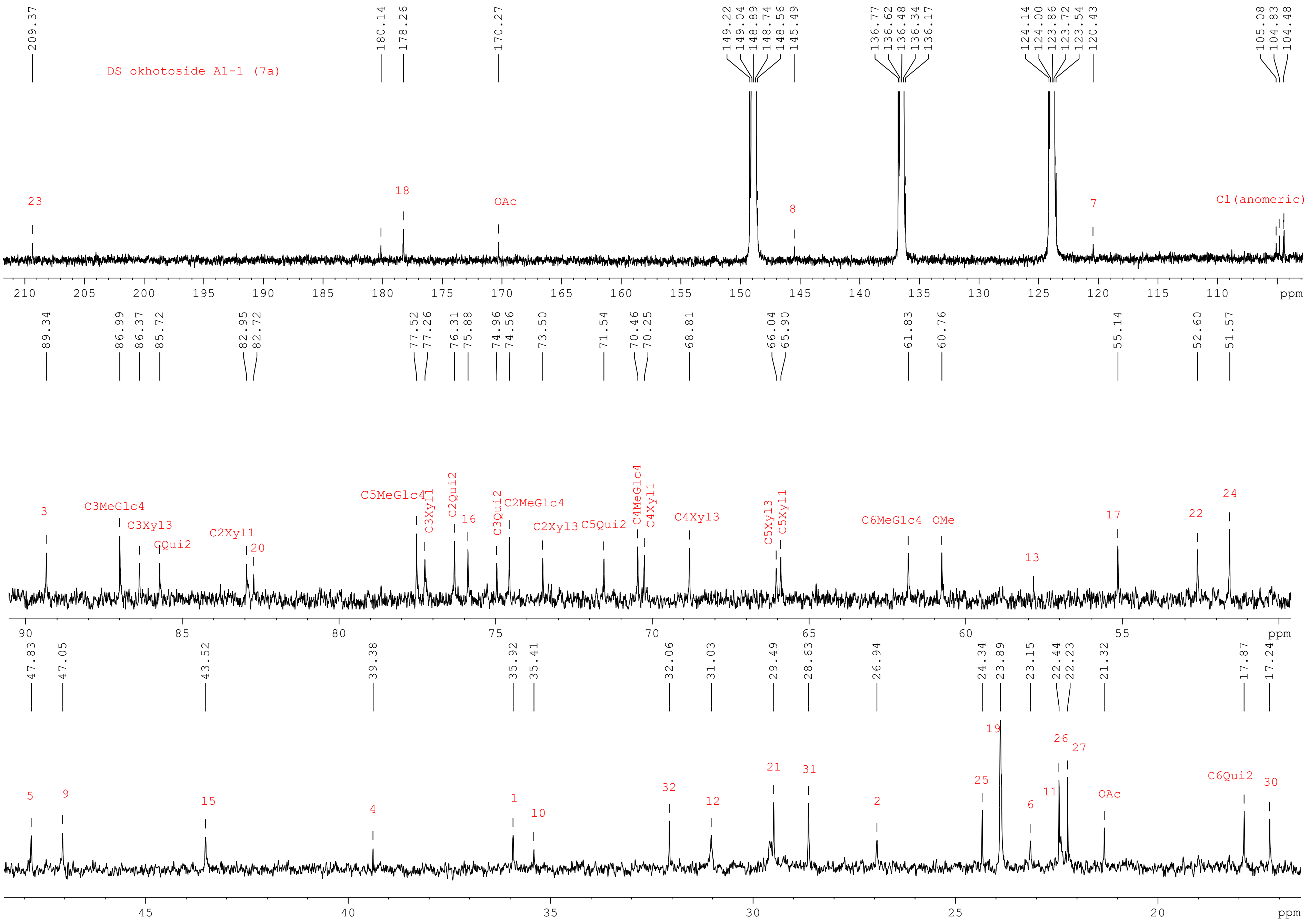

Figure S53. Assigned ^{13}C NMR spectrum of desulfated derivative okhotoside A1-1 (**7a**) isolated from *C. conicospermium*

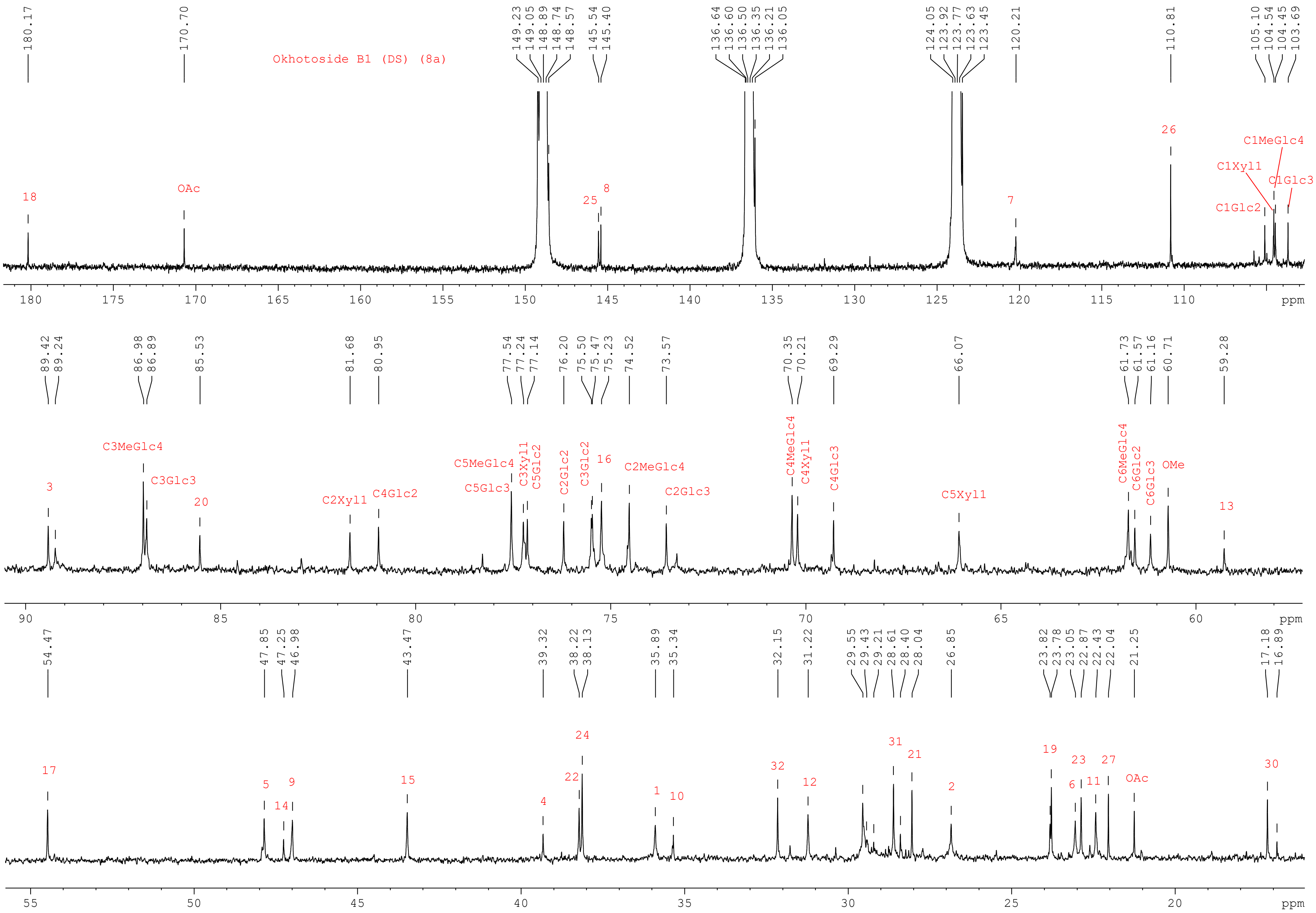

Figure S54. Assigned ¹³C NMR spectrum of desulfated derivative of okhotoside B1 (**8a**) isolated from *C. conicospermium*

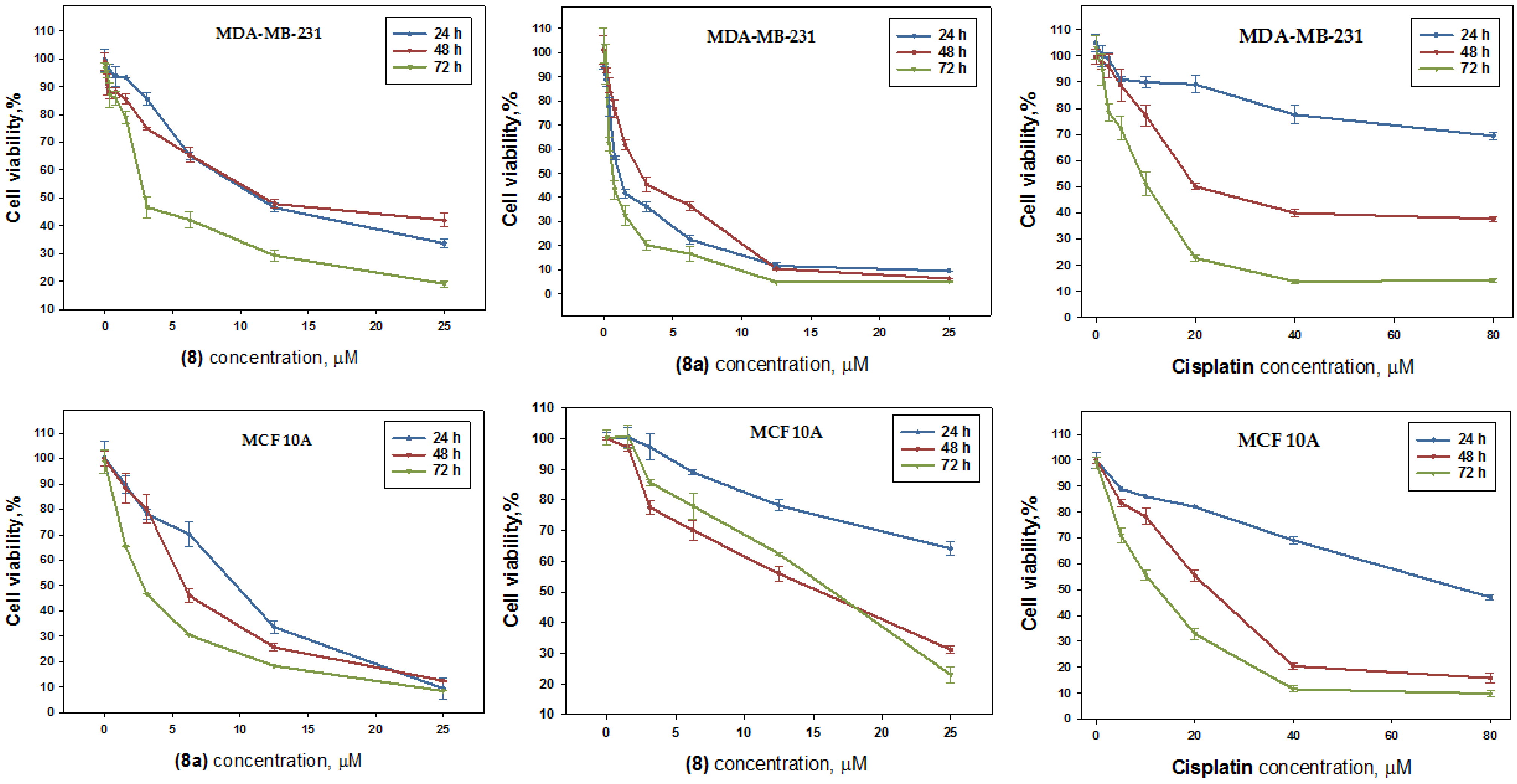

Figure S55. Cytotoxic effects of okhotoside B_1 (**8**), DS-okhotoside B_1 (**8a**) and cisplatin against MDA-MB-231 and MCF 10A cells for 24 h, 48 h, and 72 h. All experiments were carried out in triplicate. The data are presented as mean ± SEM.

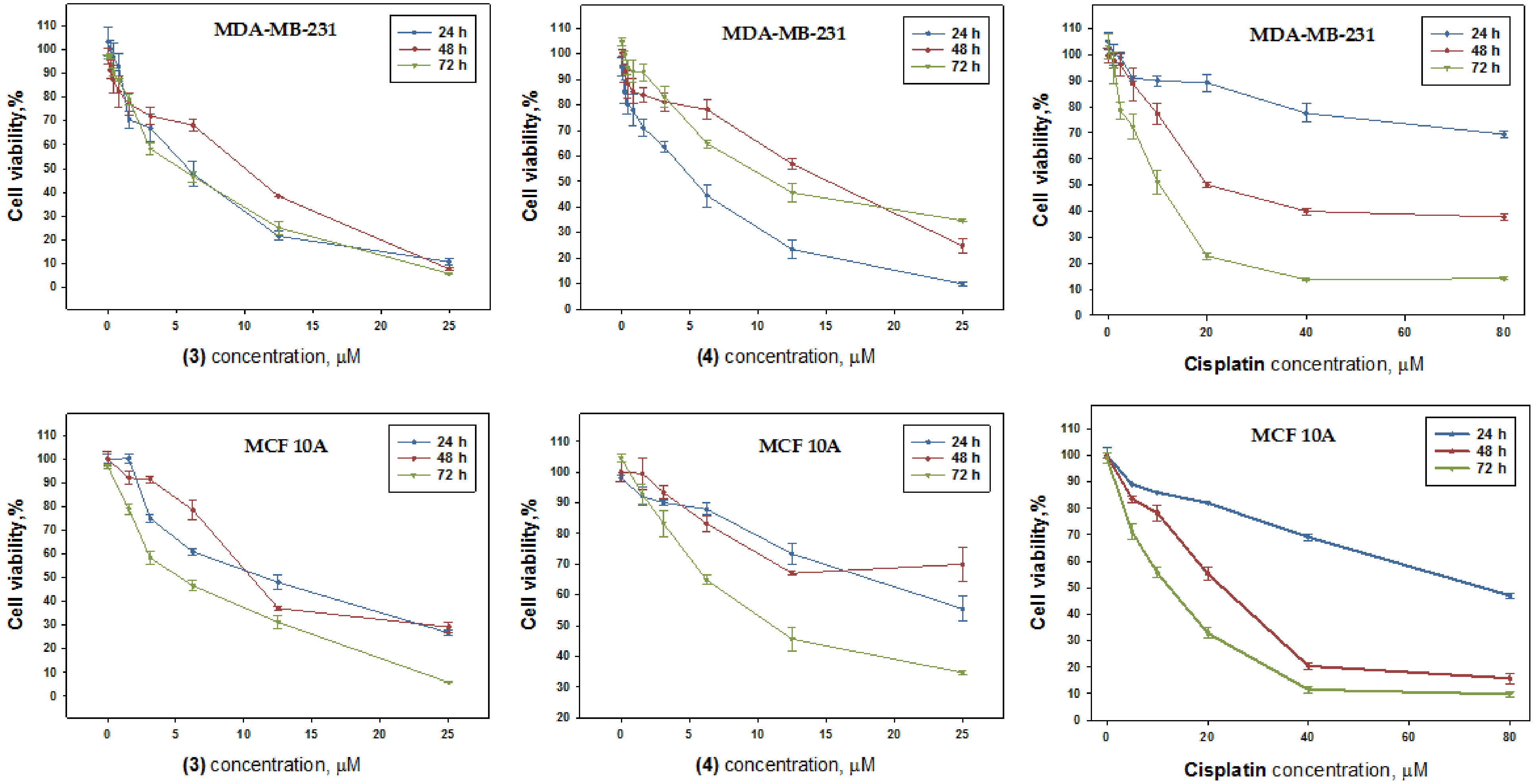

Figure S56. Cytotoxic effects of conicospermiumosides A_3-3 (**3**) and A_7-1 (**4**) and cisplatin against MDA-MB-231 and MCF 10A cells for 24 h, 48 h, and 72 h. All experiments were carried out in triplicate. The data are presented as mean ± SEM.

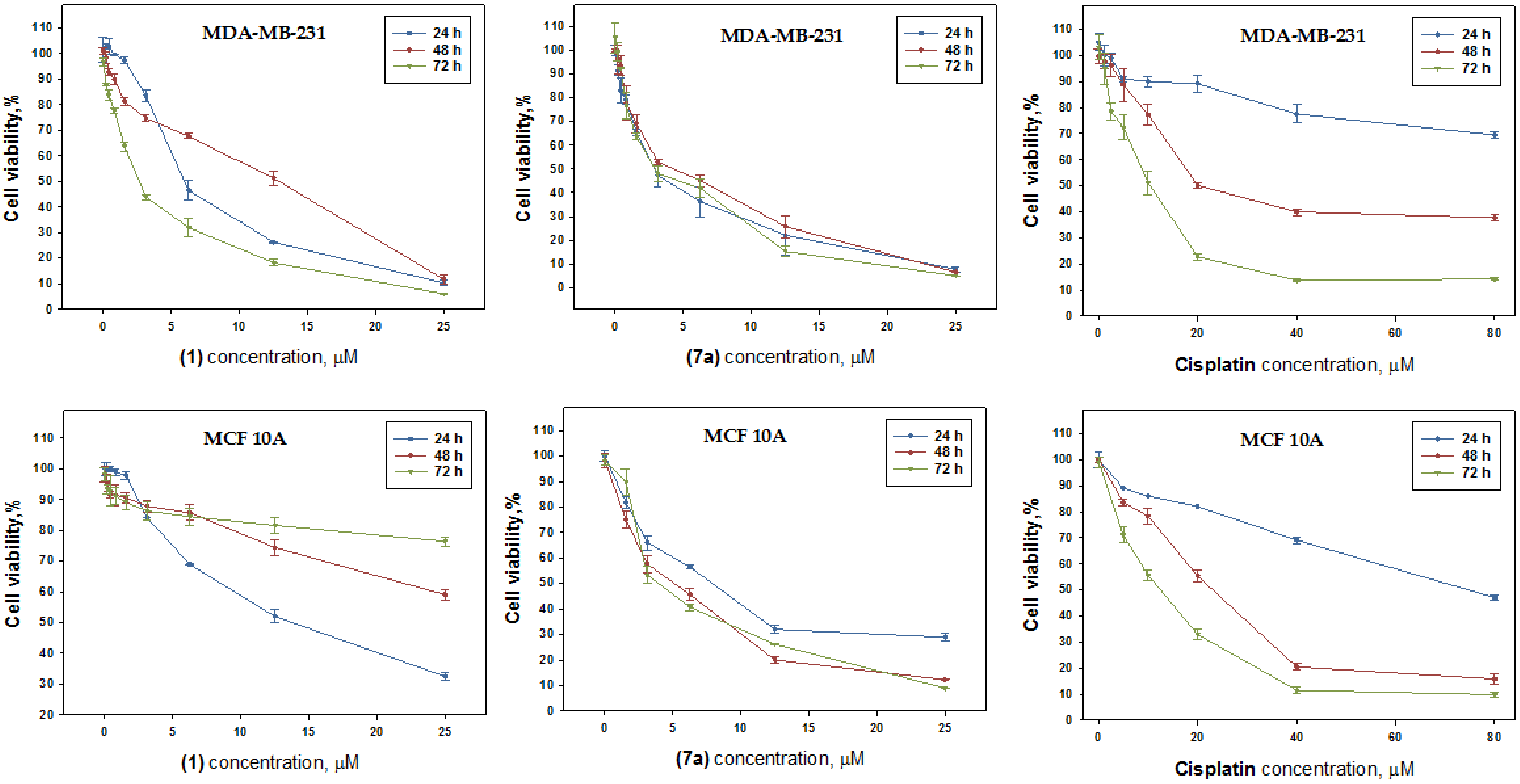

Figure S57. Cytotoxic effects of conicospermiumoside A$_3$-1 (**1**), DS-okhotoside A$_1$-1 (**7a**) and cisplatin against MDA-MB-231 and MCF 10A cells for 24 h, 48 h, and 72 h. All experiments were carried out in triplicate. The data are presented as mean ± SEM.

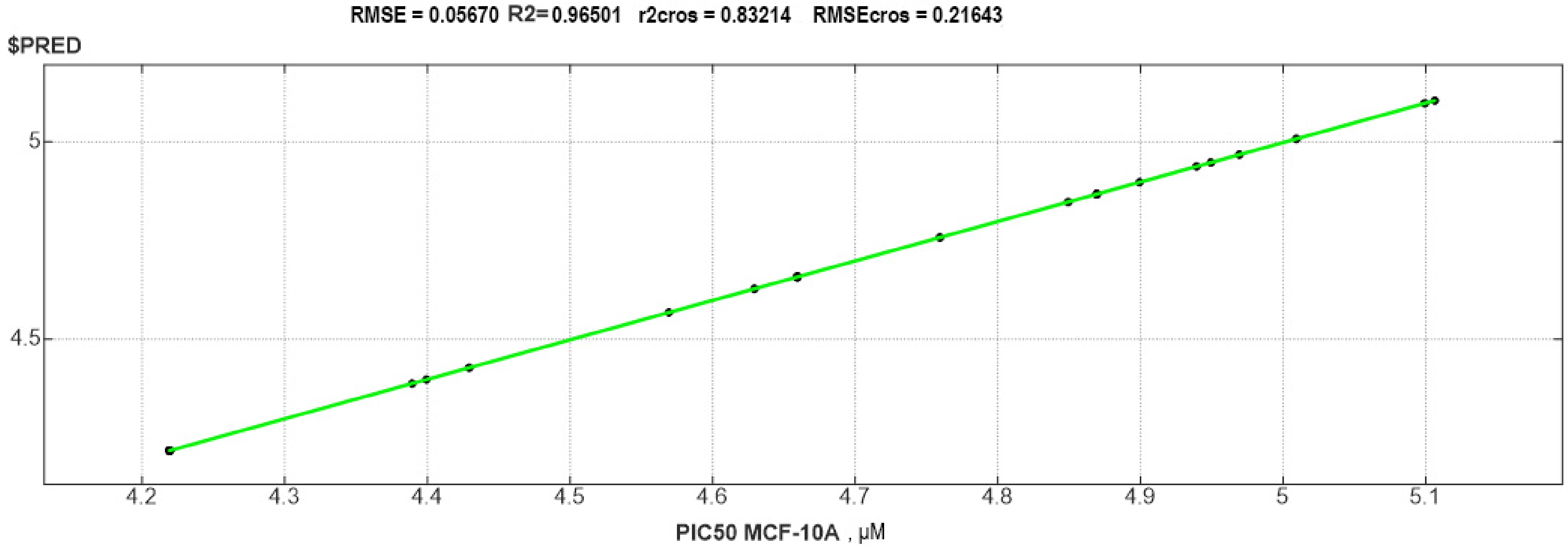

Figure S58. PLS QSAR model correlation plot reflecting the relationship of predicted and experimental cytotoxic activity against MCF-10A cells. The cytotoxic action expressed as pIC_{50}.

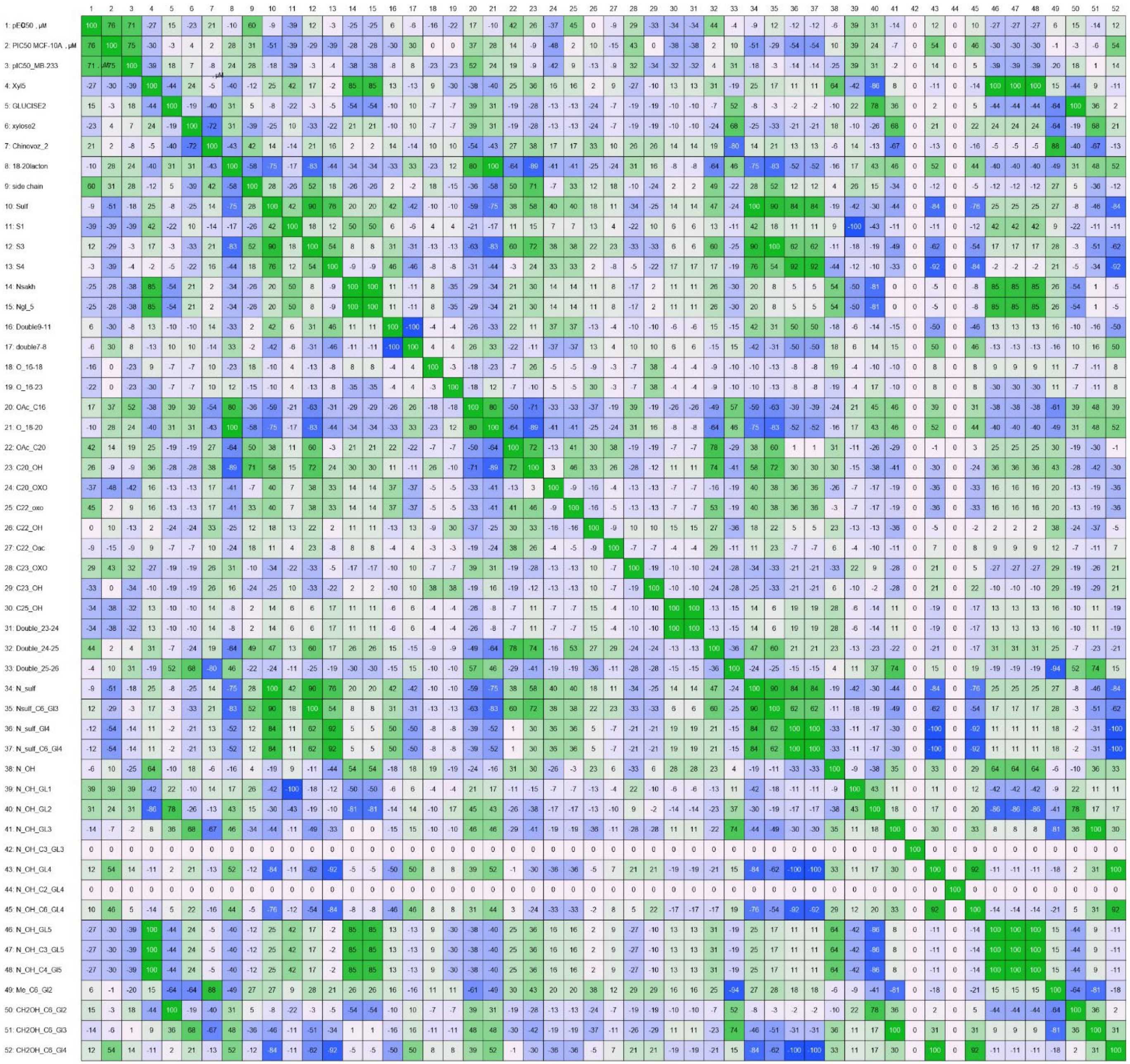

Figure S59. Correlational matrix of descriptors impacts.
